# Peretinoin, an acyclic retinoid, improves the hepatic gene signature of chronic hepatitis C following curative therapy of hepatocellular carcinoma

**DOI:** 10.1186/1471-2407-13-191

**Published:** 2013-04-15

**Authors:** Masao Honda, Taro Yamashita, Tatsuya Yamashita, Kuniaki Arai, Yoshio Sakai, Akito Sakai, Mikiko Nakamura, Eishiro Mizukoshi, Shuichi Kaneko

**Affiliations:** 1 Department of Gastroenterology, Graduate School of Medicine, Kanazawa University, 13-1Takara-machi, Kanazawa 920-0934, Japan; 2 Department of Advanced Medical Technology, Graduate School of Health Medicine, Kanazawa University, 13-1Takara-machi, Kanazawa 920-8641, Japan

**Keywords:** Acyclic retinoid, Gene expression, Hepatocellular carcinoma

## Abstract

**Background:**

The acyclic retinoid, peretinoin, has been shown to be effective for suppressing hepatocellular carcinoma (HCC) recurrence after definitive treatment in a small-scale randomized clinical trial. However, little has been documented about the mechanism by which peretinoin exerts its inhibitory effects against recurrent HCC in humans *in vivo*.

**Methods:**

Twelve hepatitis C virus-positive patients whose HCC had been eradicated through curative resection or ablation underwent liver biopsy at baseline and week 8 of treatment with either a daily dose of 300 or 600 mg peretinoin. RNA isolated from biopsy samples was subjected to gene expression profile analysis.

**Results:**

Peretinoin treatment elevated the expression levels of *IGFBP6*, *RBP1*, *PRB4*, *CEBPA*, *G0S2*, *TGM2*, *GPRC5A, CYP26B1*, and many other retinoid target genes. Elevated expression was also observed for interferon-, Wnt-, and tumor suppressor-related genes. By contrast, decreased expression levels were found for mTOR- and tumor progression-related genes. Interestingly, gene expression profiles for week 8 of peretinoin treatment could be classified into two groups of recurrence and non-recurrence with a prediction accuracy rate of 79.6% (*P*<0.05). In the liver of patients with non-recurrence, expression of *PDGFC* and other angiogenesis genes, cancer stem cell marker genes, and genes related to tumor progression was down-regulated, while expression of genes related to hepatocyte differentiation*,* tumor suppression genes, and other genes related to apoptosis induction was up-regulated.

**Conclusions:**

Gene expression profiling at week 8 of peretinoin treatment could successfully predict HCC recurrence within 2 years. This study is the first to show the effect of peretinoin in suppressing HCC recurrence *in vivo* based on gene expression profiles and provides a molecular basis for understanding the efficacy of peretinoin.

## Background

Hepatocellular carcinoma (HCC) is the sixth most common form of cancer worldwide, and it is estimated that there are more than 740,000 new cases each year [1]. Early-stage HCC is indicated for definitive treatment by surgical resection or local therapy [2-4]; however, the prognosis of HCC is typically poor, and around 50% of patients experience recurrence within 3 years of definitive therapy [5-7]. Indeed, some researchers estimate that the 3-year recurrence rate is higher than 70% for hepatitis C virus (HCV)-positive patients [8], and past clinical experience with interferon-based therapy, systemic chemotherapy, and other treatment modalities has shown the lack of effective standard therapy for suppressing tumor recurrence after definitive treatment for HCC [9-11].

Peretinoin (NIK-333) has only been reported to suppress HCC recurrence in a small-scale randomized controlled trial [12] in which patients who were disease-free after definitive treatment received oral administration of 600 mg peretinoin daily for one year. The results showed that peretinoin significantly reduced the incidence of recurrent or new HCC [12] and improved patient survival rates [13]. Based on the results of rat pharmacological studies [14,15] and a phase I clinical study of peretinoin [16], a phase II/III clinical study of peretinoin was conducted in which the doses were set at 300 and 600 mg daily. The study demonstrated that, in the Child-Pugh A subgroup, 600 mg/day peretinoin (n=100) reduced the risk of HCC recurrence or death by approximately 40% compared to placebo (n=106) [hazard ratio (HR)=0.60; 95% confidence interval (CI): 0.40–0.89)] [17]. On the other hand, 300 mg daily doses of peretinoin were insufficient for tumor control and showed no substantial difference from the placebo [17]. A large-scale clinical study including several countries is now planned to confirm the clinical efficacy of peretinoin.

Little is known about the mechanism by which peretinoin exerts its inhibitory effects against recurrent HCC in humans *in vivo*. In order to investigate this mechanism, we conducted here a comparative study recruiting HCV-positive patients who successfully completed definitive treatment for HCC (similar to the phase II/III clinical study mentioned above). Patients underwent liver biopsy before and after 8 weeks of treatment with repeated doses of peretinoin, and the collected samples were analyzed for gene expression profiling using the remnant liver after eradication of HCC. We found that changes in the gene expression signature observed in this study help us to understand the means by which peretinoin suppresses HCC, in particular its inhibition against *de novo* carcinogenesis.

## Methods

### Patients

We enrolled 12 HCV-positive patients who were cured of their primary and first recurrent HCC by surgical hepatectomy or radiofrequency ablation therapy and other non-surgical local treatments (Table 1). Complete tumor removal was confirmed by dynamic computed tomography (CT) scans. Inclusion criteria were as follows: positive presence of HCV-RNA in the serum; Child-Pugh class A or B liver function; platelet counts ≥50,000/μL; and age ≥20 years. Exclusion criteria included the following: positive for hepatitis B surface antigen; tumor infiltration into the portal vein; use of transarterial embolization or transarterial chemoembolization (TAE/TACE) for definitive therapy; postoperative use of investigational medicinal products, antitumor agents, interferon, or vitamin K2 formulations; blood pressure unmanageable even with medication (systolic pressure ≥160 mmHg or diastolic pressure ≥100 mmHg); complication with renal impairment, cardiovascular disease, diabetes mellitus, autoimmune disease, asthma, or other severe disease; presence of neoplasm; allergy to CT contrast media; allergy to retinoids; history of total gastrectomy; possible pregnancy during study; and lactating mothers.

**Table 1:** Patient characteristics and prognosis

**Patient**	**Dose**	**Age**	**Sex**	**P/R**	**Curative**	**MTD**	**Tumor no.**	**Tumor**	**Background liver**		**CP**	**ALT**	**PLT**	**Prognosis**	
**no.**					**treatment**			**histology**	**F**	**A**				**2 yrs**	**4.5 yrs**
1	300	70	F	P	RFA	15	2	m-p	4	2	A	112	7.9	Rec	Rec
2	600	72	F	R	RFA	20	2	w	4	2	A	40	7.9	Rec	Rec λ
3	300	58	M	P	resection	25	1	m-p	2	1	A	16	19.2	nonRec	nonRec
4	600	54	M	P	resection	25	1	m-p	3	2	A	57	16.4	nonRec	Rec
5	600	60	F	P	RFA	23	1	m-p	4	2	B	23	6.4	nonRec	nonRec
6	300	73	F	P	RFA	20	2	m-p	3	2	A	31	14.2	Rec	Rec λ
7	300	69	F	P	RFA	11	3	w-m	4	1	A	38	11.5	Rec	Rec λ
8	600	74	F	P	RFA	16	2	m-p	4	1	A	45	5.1	nonRec	Rec
9	600	65	M	R	RFA	10	1	m-p	2	1	A	29	16.5	nonRec	nonRec
10	600	59	M	P	resection	34	1	m-p	4	2	B	60	9.4	nonRec	nonRec
11	300	70	F	R	RFA	15	1	w-m	4	2	B	98	7	nonRec	nonRec
12	300	66	M	P	RFA	15	1	m-p	4	1	A	90	10.6	nonRec	nonRec

Dose (mg/day), ALT(U/L), PLT(×10^4^/μL), MTD (mm).

F; female, M; male, P; primary HCC, R; (first) recurrent HCC, MTD; maximum tumor diameter, w; well-differentiated, m; moderately differentiated, p; poorly differentiated, F; fibrosis stage, A; activity grade, CP; Child-Pugh classification, ALT; alanine aminotransferase, PLT; platelet.

Rec; recurrence, nonRec; non-recurrence, λ; death.

### Study design

**Figure 1:**
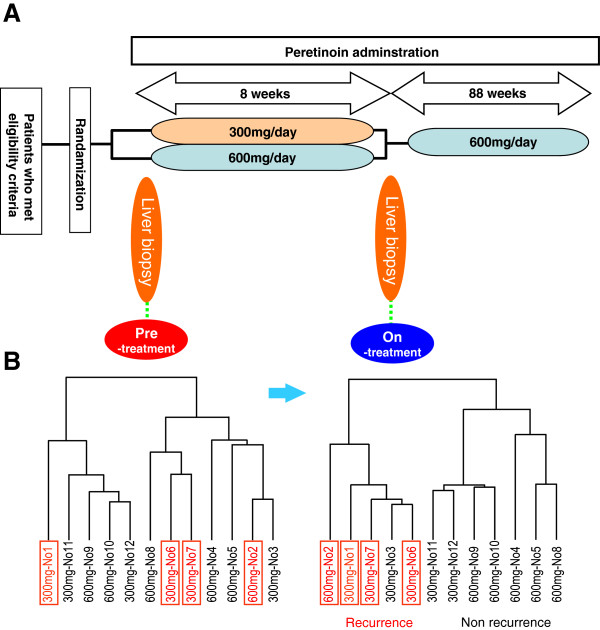
**Peretinoin pharmacokinetics study design and change of gene expression profiling. A**: Peretinoin pharmacokinetics study design. Twelve patients were enrolled in the study and two groups of 6 patients were randomly administered one of two doses of peretinoin (600 or 300 mg per day) for 8 weeks. A liver biopsy was obtained before the start of peretinoin administration and 8 weeks into the treatment. After the second liver biopsy, oral administration of peretinoin twice daily was resumed for 88 weeks. **B**: Hierarchical clustering analysis of patients using all expressed genes. Patient numbers (Table 1) and peretinoin dose are listed. Patients with HCC recurrence are shown in red and boxed. There was no significant association between hepatic gene expression and HCC recurrence before starting peretinoin treatment, while distinct clusters of patients were observed after week 8 depending on HCC recurrence.

This trial was a randomized, parallel-group, open-label study. Twelve eligible patients signed the informed consent form for registration. They were randomized to receive one of the two peretinoin doses: 600 or 300 mg per day. Each dose group consisted of 6 patients. After randomization, patients underwent liver biopsy before the start of peretinoin treatment, then orally received peretinoin twice daily for 8 weeks. At the end of the 8-week therapy, they underwent a second liver biopsy (Figure 1A). The collected biopsy samples were kept in RNAlater^®^ solution (Ambion Inc., Austin, TX) at 4°C overnight or longer. Within 3 days, the biopsy samples were removed from the RNAlater solution and partially subjected to RNA extraction and purification. The purified RNA samples were stored at -80°C until required for gene expression profiling. The remaining part of the biopsy samples was used to determine the intrahepatic peretinoin concentration. Samples were placed in polypropylene bottles containing 99.5% ethanol, and the air in the bottle was purged with argon. The bottles were tightly closed and stored at -80°C protected from light. Peripheral blood samples were also collected for the analysis of gene expression signatures and to determine plasma peretinoin levels.

After the second biopsy, patients were orally administered peretinoin twice daily for 88 weeks. During the treatment period, patients visited the hospital every 4 weeks for check-ups, drug compliance, and protocol-specified medical examinations. Drug compliance was assessed by pill counts. During the study, use of anticancer agents, interferon, vitamins K and A, and antiviral drugs (e.g., rivabirin) was prohibited. The study was registered at the Japan Pharmaceutical Information Center (JapicCTI-121757). This protocol was approved by the Institutional Review Board of Kanazawa University for clinical investigation following the provisions of Helsinki, Good Clinical Practice guidelines, local laws, and regulations. Written informed consent was obtained from all patients involved in this study. The detail protocol of this study is presented in Additional file 1: Study protocol.

### Plasma peretinoin concentration

A 5-mL blood sample was drawn into an EDTA-2Na tube, immediately mixed, and centrifuged to obtain a plasma sample. The air in the sample tubes was replaced with argon, and the tubes were stored at -80°C protected from light. The plasma concentrations of the unchanged form of peretinoin and its lipid-bound form were determined as follows: first, the peretinoin-containing fractions were extracted from the plasma samples, then subjected to derivatization of peretinoin, and the concentration of the derivative was measured by liquid chromatography-atmospheric pressure chemical ionization-tandem mass spectrometry.

### Liver peretinoin concentration

Collected liver tissue samples were immersed in 99.5% ethanol in containers, and the internal air was replaced with argon. The samples were stored at -80°C protected from light. The liver concentrations of the unchanged form of peretinoin and its lipid-bound form were determined as for the plasma concentrations above.

### Microarray analysis

For gene expression profiling of the liver, in-house cDNA microarrays containing a representative panel of 10,000 liver-specific genes (Kanazawa liver chip 10K ver. 2.0) were used. RNA isolation, amplification of antisense RNA, labeling, and hybridization were conducted as previously described [18].

To identify genetic variants, paired *t*-tests were performed using BRB-Array Tools software (http://linus.nci.nih.gov/BRB-ArrayTools.html) to define *P*-values <0.05 as gene variants. Hierarchical cluster analysis, exploration of significantly expressed genes, and class prediction were also performed using the BRB-Array Tools.

Hierarchical clustering was carried out using centered correlation and average linkage. The class comparison tool in the BRB-Array Tools was used to extract significantly expressed genes. Genes whose expression levels were significantly different between two groups were located by the *t*-test at the *P*<0.002 significance level. Univariate permutation tests were repeated 1,000–2,000 times to control for errors. Class prediction was performed using the above-mentioned significantly differentiated genes as discriminators, and the results were cross-validated using seven algorithms: compound-covariate predictor, diagonal linear discriminant analysis, 1-nearest neighbor, 3-nearest neighbors, nearest centroid, support vector machine, and Bayesian compound covariate. The mean value of the seven success rates for class prediction was defined as the prediction accuracy rate [18].

Pathway analysis was performed using MetaCore™ (Thomson Reuters, New York, NY) and functional ontology enrichment analysis was performed to find differentially expressed pathway using differentially expressed genes [18,19].

The microarray data have been submitted to the Gene Expression Omnibus (GEO) public database at NCBI (Accession No. GSE29302).

### Quantitative real-time detection polymerase chain reaction

Quantitative real-time detection polymerase chain reaction (RTD-PCR) was performed using the TaqMan Universal Master Mix (PE Applied Biosystems, Foster City, CA). Primer pairs and probes were purchased from the TaqMan assay reagents library. Standard curves were generated for each assay using RNA derived from normal human liver tissue. Expression data were normalized by GAPDH, and the results are shown as the relative fold expression to the normal liver.

### Statistical analysis

Results are expressed as means ± S.D. Significance was tested by one-way ANOVA with Bonferroni’s method, and differences were considered statistically significant at *P*<0.05.

## Results

### Safety

In this study, 88 adverse events were recorded in 12 patients (100%). Major adverse events included rhinopharyngitis (n=7), blood pressure elevation (n=5), peripheral edema (n=3), and enteritis (n=3). Most of these adverse events were mild or moderate, and were adequately controlled. Nine serious adverse events were documented in 5 patients, including hyperglycemia (n=2) and coronary stenosis (n=1). However, all reported serious adverse events were alleviated with appropriate treatment, and there was no substantial concern identified regarding the safety of peretinoin.

### Plasma peretinoin concentration

Plasma peretinoin concentrations were determined at week 8 of treatment. The mean (± SD) plasma concentrations of the unchanged form of peretinoin were 82.3 (± 90.0) and 201.2 (± 111.4) ng/mL at 4 h post-dose and 35.8 (± 49.2) and 29.0 (±17.9) ng/mL at 8 h post-dose for the 300 and 600 mg per day groups, respectively. The plasma concentrations of the unchanged peretinoin measured at 4 h post-dose (≈ t_max_) were dose-dependent. The mean (± SD) plasma concentrations of the lipid-bound form of peretinoin were 1478.8 (± 853.7) and 2789.8 (± 1630.0) ng/mL at 4 h post-dose and 1227.8 (± 942.7) and 2213.2 (± 1156.1) ng/mL at 8 h post-dose for the 300 and 600 mg per day groups, respectively. The plasma concentrations of the lipid-bound form of peretinoin were dose-dependent at 4 and 8 h post-dose.

### Liver peretinoin concentration

Liver peretinoin concentrations were determined at week 8 of treatment. The measurements of the liver concentration of the unchanged form of peretinoin were all below the lower limit of quantitation at 4 h post-dose for all 6 patients in the 300 mg per day group. For the 600 mg per day group, 2 patients yielded measurements of 0.052 and 0.059 μg/g, while the remaining 4 patients produced results under the lower limit of quantitation (0.050 μg/g). The mean (± SD) concentrations of the lipid-bound form of peretinoin were 13.7508 (± 11.1097) and 12.8345 (± 8.7048) μg/g for the 300 and 600 mg per day groups, respectively.

### Gene expression analysis

To analyze the gene expression signature of the liver tissue, we identified genes whose expression levels were significantly different before and after the start of the peretinoin treatment (Figure 1A). The identified genes were candidates for peretinoin-responsive genes. The phase II/III clinical study showed that a daily dose of 600 mg peretinoin reduced the risk of HCC recurrence, while a 300 mg dose was not significantly different from the placebo [17]. Therefore, gene expression patterns were compared before and after the start of the 600 mg peretinoin therapy (n=6). Consequently, 424 hepatic genes showed significantly different expression levels from baseline at week 8 (enhancement and suppression seen for 190 and 234 genes, respectively). Typical examples of these genes are represented in Table 2 where fold changes of gene expression for the 300 mg and 600 mg doses are shown respectively. In addition to the retinoid-induced genes, genes related to interferon, tumor suppressors, negative regulators of Wnt signaling, insulin-like growth factor (IGF) signaling, and hepatocyte differentiation were significantly up-regulated by peretinoin. By contrast, genes related to the mammalian target of rapamycin (mTOR), tumor progression, cell cycle, and metastasis/angiogenesis were down-regulated. Serial changes in peretinoin-responsive gene expression are shown in Additional file 2: Figure S1. Significant changes in expression were observed in response to 600 mg of peretinoin, while changes in expression were minimal with 300 mg of peretinoin.

**Table 2:** Representative genes significantly up-regulated or down-regulated in response to peretinoin treatment

**Parametric p-value**	**Ratios (Under/Pre)**		**Description**	**Symbol**	**GB acc**
**600 mg**	**600 mg**	**300 mg**			
**Up-regulated genes in response to peretinoin treatment**					
**Retinoid target genes**					
0.0002	1.85	1.25	Cytochrome P450, family 26, subfamily B, polypeptide 1	CYP26B1	NM_019885
0.004	1.75	1.33	Insulin-like growth factor binding protein 6	IGFBP6	NM_002178
0.005	1.42	1.16	Regulatory factor X-associated ankyrin-containing protein	RFXANK	NM_134440
0.006	1.33	1.30	Putative lymphocyte G0/G1 switch gene	G0S2	NM_015714
0.013	1.54	0.90	Retinol binding protein 1	RBP1	NM_002899
0.014	1.56	0.87	Retinol binding protein 4	RBP4	NM_006744
0.034	1.27	1.07	Retinoic acid induced 3	GPRC5A	AI923823
0.040	1.22	1.19	Transglutaminase 2	TGM2	AI962033
0.044	1.23	1.14	CCAAT/enhancer binding protein (C/EBP), alpha	CEBPA	NM_004364
**Interferon-related genes**					
0.029	1.45	0.93	Guanylate binding protein 1, interferon-inducible, 67kDa	GBP1	NM_002053
0.047	1.39	0.94	Interferon-induced protein 44	IFI44	NM_006417
0.048	1.28	1.05	Chemokine (C-X-C motif) ligand 9	CXCL9	NM_002416
**Negative regulator of Wnt and TGF-β signaling**					
0.004	1.54	1.06	BMP and activin membrane-bound inhibitor homolog	BAMBI	NM_012342
0.008	1.45	1.11	Secreted frizzled-related protein 5	SFRP5	NM_003015
**Anti-angiogenesis**					
0.021	1.37	0.98	Thrombomodulin	THBD	NM_000361
0.038	1.28	0.99	Protein C receptor, endothelial (EPCR)	PROCR	NM_006404
**Tumor suppressor related**					
0.029	1.35	0.96	Jumonji domain containing 3	JMJD3	XM_043272
0.029	1.39	0.91	Jumping translocation breakpoint	JTB	NM_006694
0.034	1.39	1.32	Protein kinase, AMP-activated, alpha 2 catalytic subunit	PRKAA2	NM_006252
**Down-regurated genes in response to peretinoin treatment**					
**mTOR-related-gene**					
0.045	0.78	0.94	FK506 binding protein 12-rapamycin associated protein 1	FRAP1	NM_004958
**Cytokine and growth factor**					
0.019	0.77	1.25	Interleukin 13	IL13	NM_002188
0.031	0.74	1.00	Hepatocyte growth factor	HGF	NM_000601
**Tumor progression related**					
0.011	0.73	0.94	Junctional adhesion molecule 3	JAM3	NM_032801
0.013	0.70	1.00	V-myc myelocytomatosis viral oncogene homolog	Myc	NM_002467
0.017	0.73	1.12	Src-like-adaptor	SLA	NM_006748
0.028	0.78	1.10	Cell division cycle 2, G1 to S and G2 to M	CDC2	NM_001786
0.030	0.66	0.95	BCL2-associated athanogene	BAG1	NM_004323
0.039	0.64	0.93	Chemokine (C-C motif) receptor 9	CCR9	NM_031200
0.043	0.76	1.13	Pre-B-cell leukemia transcription factor 1	PBX1	H08835

The peretinoin-response genes were identified by comparing hepatic gene expression in the pre and under treatment of 6 patients who were treated with 600 mg dose of peretinoin. The fold changes of gene expression are shown in 300 mg and 600 mg dosage respectively.

Hierarchical clustering of patients using hepatic gene expression prior to administering peretinoin revealed no significant association with clinical outcome, but a significant association became clearly apparent 8 weeks after peretinoin treatment (Figure 1B). The patients were clustered into two groups: one containing patients with HCC recurrence (4 of 5 patients had recurrence) and the other containing those without recurrence (all 6 patients were recurrence free) within 2 years. Supervised learning methods using seven different algorithms showed that the patients receiving treatment could be differentiated into two groups with or without recurrence by 224 gene predictors (*P*<0.002) at 79.6% accuracy (*P*<0.05) (Table 3). Interestingly, 44 of 224 (20%) genes were peretinoin induced.

**Table 3:** Supervised learning methods

**Class**		**No. of predictors**	**Prediction**	**p-value**
		**(p<0.002)**	**(%)**	
Pre-treatment	Recurrence vs non-recurrence	6	47.1	N.S.
On-treatment	Recurrence vs non-recurrence	224	79.6	< 0.05
On-treatment	300 mg vs 600 mg	38	72.7	N.S.

Seven algorithms of Compound-Covariate Predictor, Diagonal Linear Discriminant Analysis 1-Nearest Neighbor, 3-Nearest Neighbors, Nearest Centroid, Support Vector Machine, and Bayesian Compound Covariate were used for class prediction. Prediction % was calculated as the average of these seven algorithms.

Although peretinoin-responsive genes were more induced in patients treated with the 600 mg dosage, gene expression profiling 8 weeks after peretinoin treatment could not be classified according to the dosage (Table 3). This might be because two patients treated with the 300 mg dosage (No. 11 and No. 12) had already expressed high levels of peretinoin-response genes before starting peretinoin treatment (Additional file 2: Figure S1). Interestingly, patients with high levels of peretinoin-response genes before treatment (No. 9–12) did not show HCC recurrence during the entire observation period (4.5 years; Table 1).

**Figure 2:**
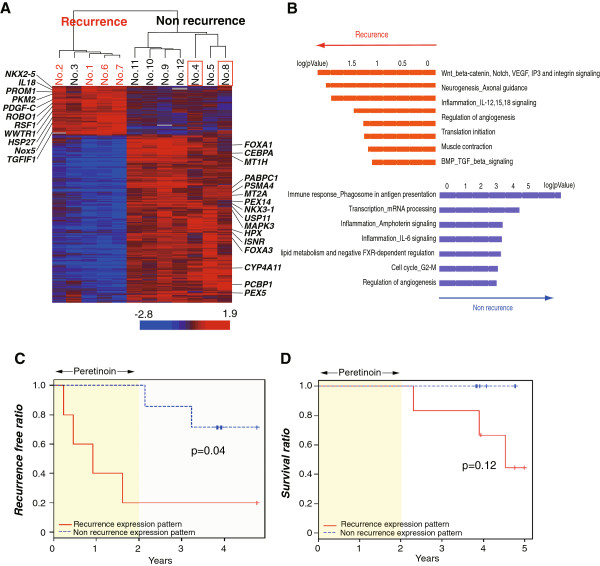
**Expression profiling of 224 gene predictors and the prognosis of patients. A**: Hierarchical clustering using 224 gene predictors of patients with or without HCC recurrence. Patients with HCC recurrence within 2 years are shown in red and patients with HCC recurrence after the cessation of peretinoin are boxed in red. **B**: Pathway analysis of differentially expressed genes using MetaCore (GeneGo). Functional ontology enrichment analysis was performed to find differentially expressed pathway maps or map folders using 224 differentially expressed genes (*p*<0.002) between patients with and without HCC recurrence. **C**, **D**: Kaplan-Meier estimation of recurrence-free ratio (**C**) and survival ratio (**D**) of patients with recurrence expression patterns (red) and those with non-recurrence expression (blue).

Hierarchical clustering of all 12 patients using 224 gene predictors is shown in Figure 2A. Clear gene clusters were observed according to patients with recurrence and those without, with the exception of one patient (No. 3, Table 1). Interestingly, in the liver of patients with non-recurrence, genes related to angiogenesis, cancer stem cells, Wnt signaling, and tumor progression were repressed, while genes inducing differentiation, tumor suppression, and apoptosis were up-regulated (Figure 2B, Table 4). Interestingly, PDGF-C was the most significant predictor to differentiate patients who will experience recurrence within 2 years (Table 4).

**Table 4:** Representative genes differentially expressed between HCC recurrence and non-recurrence groups

**Parametric p-value**	**t-values**	**Description**	**Symbol**	**GB acc**
**Up-regulated genes in the recurrence group**				
** Angiogenesis related**				
0.0001	-5.19	Platelet derived growth factor C	PDGFC	AI446155
0.0006	-4.37	Sperm equatorial segment protein 1	NOX5	NM_145658
0.0010	-4.13	Interleukin 18	IL18	AI800476
** Cancer stem cell related**				
0.0004	-4.63	Prominin 1	PROM1	NM_006017
0.0018	-3.83	Pyruvate kinase, muscle	PKM2	NM_002654
** Positive regulator of Wnt**				
0.0018	-3.84	TGFB-induced factor (TALE family homeobox)	TGIF1	AI866302
0.0018	-3.84	NK2 transcription factor related, locus 5	NKX2-5	NM_004387
** Tumor progression related**				
0.0005	-4.47	Transcriptional co-activator with PDZ-binding motif	WWTR1	AK025216
0.0017	-3.87	Roundabout, axon guidance receptor, homolog 1	ROBO1	NM_133631
0.0018	-3.84	Hepatitis B virus x associated protein	RSF1	NM_016578
0.0019	-3.79	Heat shock 27kDa protein 2	HSPB2	NM_001541
**Up-regulated genes in the non-recurrence group**				
**Liver function and hepatocytes differenti related**				
0.0002	4.88	Metallothionein 2A	MT2A	NM_005953
0.0002	4.08	CCAAT/enhancer binding protein (C/EBP), alpha	CEBPA	NM_004364
0.0003	4.72	Forkhead box A3	FOXA3	NM_004497
0.0006	4.42	Hemopexin	HPX	NM_000613
0.0006	4.35	Metallothionein 1H	MT1H	NM_005951
0.0013	4.01	Forkhead box A1	FOXA1	NM_004496
0.0014	3.97	FK506 binding protein 8, 38kDa	FKBP8	NM_012181
**Tumor suppressor related**				
0.0005	4.51	Deleted in colorectal carcinoma	DCC	X76132
0.0018	3.84	NK3 transcription factor related, locus 1	NKX3-1	NM_006167
**Apoptosis inducing**				
0.0015	3.93	BH3 interacting domain death agonist	BID	NM_197967
0.0019	3.82	Programmed cell death 8	AIFM1	NM_145813

**Figure 3:**
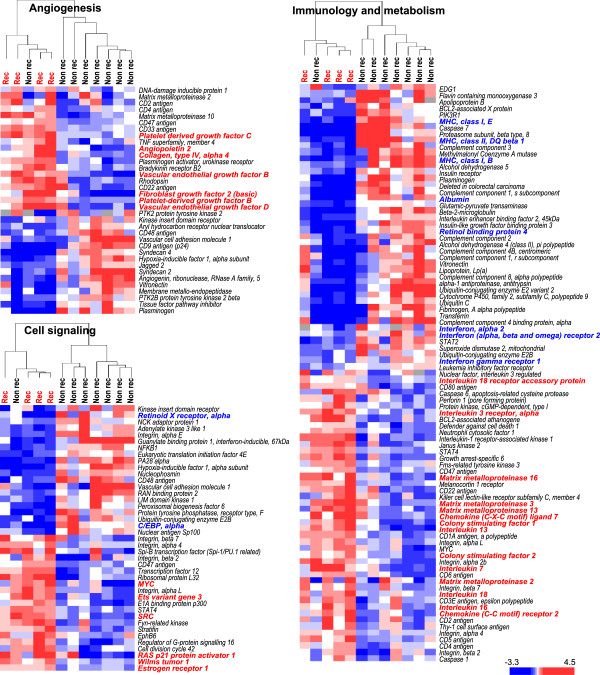
**Hierarchical clustering using pre-defined curated gene sets based on NCBI’s Cancer Genome Anatomy Project. **Presented genes were differentially expressed at *P*-values <0.05 between patients with and without HCC recurrence.

Consistent with these results, hierarchical clustering using pre-defined curated gene sets based on the NCBI’s Cancer Genome Anatomy Project similarly differentiated patients into two groups with or without HCC recurrence (Figure 3). Among angiogenesis-related genes, PDGF-C, PDGF-B, vascular endothelial growth factor (VEGF)-B, VEGF-D, and fibroblast growth factor-basic (FGF-2) were repressed in patients without recurrence. As for cell signaling-related genes, MYC, SRC, and RAS-related genes were also repressed; retinoid X receptor alpha (RXRA) and CCAAT/enhancer binding protein (C/EBP), alpha were up-regulated in patients without recurrence. Some cytokines (IL-7, IL-13, and IL-18) and chemokines (e.g. CXCL7) were repressed, while major histocompatibility complex molecules and interferon-related molecules (e.g. IFNAR2) were up-regulated in patients without recurrence (Figure 3).

**Figure 4:**
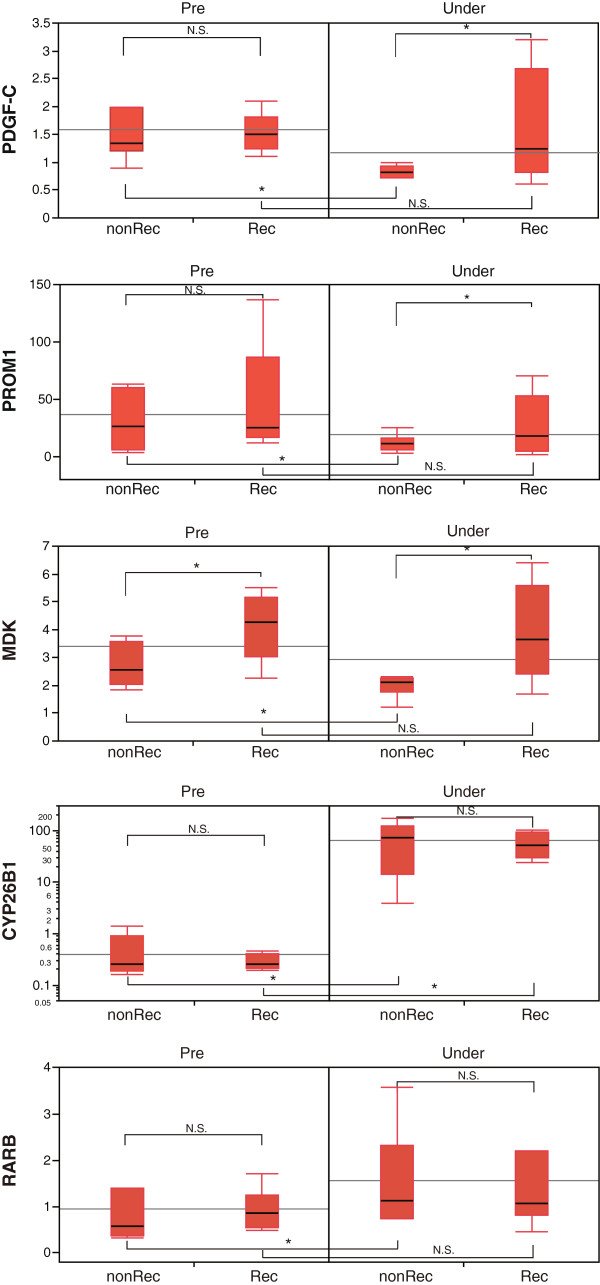
RTD-PCR evaluation of PDGF-C, PROM1, MDK, CYP26B1, and RAR β in the liver of patients with or without HCC recurrence.

cDNA microarray analysis revealed that among these predictors, the mRNA level of PDGF-C was the most significant predictor for differentiating patients who will experience recurrence within 2 years (Table 4). This observation was also assessed by RTD-PCR (Figure 4). The expression of the catalytic enzyme of retinoic acid, CYP26B1, was significantly up-regulated at around 200 fold by peretinoin treatment, but its expression was equally induced in patients with or without recurrence. However, the expression of RAR*-*β, a retinoid receptor, was significantly up-regulated by peretinoin in patients without HCC recurrence (Figure 4).

Patients were followed up for a further 3 years (mean: 2.5 ± 0.5 years) after the cessation of peretinoin treatment. Other two patients experienced recurrence during further follow up period (No. 4 and No. 8 in Figure 2A, Table 1). Three patients with recurrence died at 0.3, 1.9, and 2.5 years after the cessation of peretinoin treatment. The Kaplan-Meier estimation of the recurrence-free ratio deduced from 224 gene predictors showed significant differences in HCC recurrence between patients with the recurrence expression pattern and those with non-recurrence expression (*P*=0.04). Moreover, Kaplan-Meier estimation of the survival ratio deduced from the same gene predictors showed a trend for improved survival of patients with non-recurrence expression patterns compared with those with the recurrence expression pattern (*P*=0.12) (Figure 2C, D).

With the exception of the number of tumors at the time of curative therapy, none of the other clinical parameters (e.g. peretinoin dose, tumor, background liver histology, or background liver function) were associated with the recurrence-free or survival ratio. Thus, the peretinoin response during the early period of administration deduced from the hepatic gene expression pattern can successfully predict HCC recurrence and, potentially, patient survival.

## Discussion

Peretinoin [(2E,4E,6E,10E)-3,7,11,15-tetramethylhexadeca-2,4,6,10,14-pentaenoic acid] is expected to be a powerful agent against HCC recurrence. This synthetic retinoid induces the transcriptional activation of the retinoic acid receptor (RAR) and retinoid X receptor (RXR), which are both members of the retinoid receptor family. One primary pathway of HCC development involves sustained hepatitis virus infection, which causes repeated cycles of hepatocellular necrosis and proliferation. During increased cell proliferation, mutations occur that lead to the development of HCC unless the dedifferentiated tumor cells are eliminated by apoptosis. The anti-HCC mechanism of action of peretinoin has previously been suggested to be a result of induction of cell apoptosis [20,21], enhancement of cell differentiation [21,22], suppression of cell proliferation by elevation of P21 protein expression and suppression of cyclin D1 expression [23,24]. The first route of action is independent of retinoid receptors, while the others are retinoid receptor-dependent, although all mechanisms remain largely speculative.

Peretinoin was previously shown to suppress *in vivo* hepatocarcinogenesis in 3^′^-methyl-4-dimethylaminoazobenzene- and *N*-diethylnitrosamine-induced rats [14,15,25], and in hepatoma-bearing mice and transgenic mice expressing a dominant-negative retinoic acid receptor [25,26]. Recently, we revealed that peretinoin effectively inhibits hepatic fibrosis and HCC development in *Pdgf-c Tg* mice. This demonstrated that PDGF signaling is a target of peretinoin in preventing the development of hepatic fibrosis and HCC [27]. The purpose of this study was to investigate how peretinoin exerts its therapeutic potential by analyzing its effects on the gene expression patterns in clinical samples.

Gene expression profiling in patients without HCC recurrence demonstrated the promotion of *RAR-β* expression, the most common retinoid target gene identified by basic research. Moreover, the expression of other retinoid target genes such as *C/EBP-α*, *IGFBP6*, *TGM2*, *G0S2*, *RBP1*, *RBP4*, and *GPRC5A* was also enhanced. Of these, *C/EBP-α*, *IGFBP6*, and *TGM2* have been shown to inhibit HCC proliferation when co-expressed with *RAR-β* by all-trans-retinoic acid [28,29]. In addition, the RXR-selective agonist (rexinoid)-induced expression of *IGFBP6*, which occurs following *RAR-β*-mediated transcriptional activation of *RAR/RXR*, has been shown to suppress tumor growth [30]. Moreover, *G0S2* and *GPRC5A* have been reported to possess tumor suppressive or apoptosis-inducing effects [31,32]. These primary response retinoid target genes are presumably retinoid-responsive genes. In addition to enhancing retinoid target gene expression, peretinoin induced changes in the expression levels of a variety of genes involved in hepatocarcinogenesis, such as those related to Wnt signaling, IGF signaling, interferon, mTOR, and cell cycle regulation. These results suggest that peretinoin modulates multiple signaling cascades involved in carcinogenesis, either directly or indirectly. Abnormalities in the genes regulating Wnt signaling, IGF signaling, interferon, mTOR, and the cell cycle have been indicated to play a crucial role in the development of HCC [33,34]. We argue that peretinoin suppresses HCC cell proliferation by improving the expression of these genes, thereby preventing HCC recurrence.

The cluster analysis performed in this study successfully differentiated patients with recurrence within 2 years and those without it. Supervised learning methods identified 224 genes as predictors for HCC recurrence (*p*<0.002). Importantly, 44 (20%) of these were peretinoin-responsive genes, suggesting that recurrence-related genes might be regulated by peretinoin-responsive genes.

A comparison of these groups of patients revealed that the non-recurrence group was associated with the enhanced expression of genes related to hepatocellular differentiation and tumor suppression. The non-recurrence group also showed reduced expression of the genes promoting liver fibrosis and steatosis and the liver cancer stem cell marker genes. The genes related to hepatocellular differentiation, *MT1H*, *MT2A*, *FOXA1* (*HNF3α*), and *FOXA3* (*HNF3γ*), may be secondary response genes regulated by *C/EBP-α*[35,36]. Indeed, *C/EBP-α* manifested a significant shift in expression level before and during treatment with peretinoin, and could also differentiate between recurrence and non-recurrence within 2 years. Even after the cessation of peretinoin treatment, the expression of these genes was still significantly related to HCC recurrence (Figure 2C, D). Thus, we speculate that the differences in expression levels of peretinoin-response genes would determine the expression of recurrence-related genes (Additional file 3: Figure S2).

Interestingly, PDGF-C was the most significant predictor to differentiate those patients who will experience recurrence. Using a mouse model of PDGF-C over-expression resulting in hepatic fibrosis, steatosis, and eventually HCC development, peretinoin was previously shown to significantly repress the development of hepatic fibrosis and tumors [27].

Although gene expression profiling analysis was conducted using the remnant liver after definitive treatment in the present study, past similar research has demonstrated the possibility of predicting recurrent metachronous and multicentric HCC [37,38]. The exact mechanisms of how the expression profile of non-tumor tissues might determine the recurrence risk are not known. However, the degree of differentiation of hepatocytes and microenvironments such as angiogenesis and fibrogenesis in non-tumor lesions of the liver is likely to be closely associated with hepatocarcinogenesis. Interestingly, patients with pre-activated peretinoin-response genes were resistant to HCC recurrence for the entire observation period (4.5 years).

This study demonstrated that the patient response to peretinoin during the early period of administration could predict HCC recurrence and, potentially, patient survival. However, it should be noted that the current study protocol consisted of 600 mg peretinoin as the subsequent maintenance treatment for all patients after the 8-week start phase (Figure 1A). In addition, we did not conduct a placebo control to observe serial changes of hepatic gene expression without peretinoin administration. Therefore, there might be some limitations in drawing concrete conclusions from this study.

Although we attempted to analyze the liver peretinoin concentration in the present study to investigate its possible relationship with gene expression, peretinoin levels were too low to yield a meaningful result. However, considering that gene expression profiling identified significant changes in the expression levels of retinoid-related and other genes before and during peretinoin treatment, we believe that sufficient levels of peretinoin reached the liver.

The previous peretinoin phase II/III clinical study demonstrated that daily doses of 600 mg peretinoin significantly reduced the incidence of HCC recurrence in HCV-positive patients who underwent definitive treatment. The findings of the present study are complementary to this as we successfully identified candidates for the peretinoin-responsive and recurrence-related genes. These genes are probably involved in the inhibition of HCC recurrence and could be beneficial as future candidate biomarkers of the effectiveness of peretinoin.

## Conclusions

In this study, patients underwent liver biopsy before and after 8 weeks of treatment with repeated doses of peretinoin. Gene expression profiling at week 8 of peretinoin treatment could successfully predict HCC recurrence within 2 years. This study is the first to show the effect of peretinoin in suppressing HCC recurrence *in vivo* based on gene expression profiles and provides a molecular basis for understanding the efficacy of peretinoin.

## Abbreviations

ACR: Acyclic Retinoid; CH-C: Chronic Hepatitis C; HCC: Hepatocellular Carcinoma; HCV: Hepatitis C Virus

## Competing interests

The authors declare that they have no competing interests.

## Authors’ contributions

MH: study concept and design, manuscript preparation. TY: gene expression analysis. TY: acquisition of data of clinical data. KA: acquisition of data of clinical data. YS: gene expression analysis. AS: acquisition of data of clinical data. MN: gene expression analysis. EM: acquisition of data of clinical. SK: study concept and design. All authors read and approved the final manuscript.

## Pre-publication history

The pre-publication history for this paper can be accessed here:

http://www.biomedcentral.com/1471-2407/13/191/prepub

## Supplementary Material

Additional file 1 Study protocol. Click here for file

Additional file 2: Figure S1 One-way hierarchical clustering of up-regulated or down-regulated genes in the liver by the administration of peretinoin (300 mg and 600 mg). Changes in gene expression in the liver before the start of peretinoin administration and 8 weeks into the treatment are shown. Patients with HCC recurrence within 2 years are shown in red and those with HCC recurrence after the cessation of peretinoin are boxed in red. Click here for file

Additional file 3: Figure S2 Schematic representation of peretinoin action in the liver. Click here for file
