# Supplementary material for: Peretinoin, an acyclic retinoid, improves the hepatic gene signature of chronic hepatitis C following curative therapy of hepatocellular carcinoma
Source: BMC Cancer. 2013 Apr 15;13:191. doi: 10.1186/1471-2407-13-191 (PMC3660229; doi:10.1186/1471-2407-13-191)
Supplement: Additional file 1 — Study protocol. [file 1471-2407-13-191-S1.doc]

**Clinical Pharmacology Study of NIK-333**

— Search for drug-response gene and examination of safety in patients who were completely cured of hepatitis C virus-positive hepatocellular carcinoma —

Study Protocol

Study protocol No. : NIK-333-02

Date : July 7, 2005

Version 2 : August 12, 2005

Version 3 : October 20, 2005

Version 4 : April 20, 2006

Version 5 : October 2, 2006

Version 6 : November 6, 2006

Version 7 : November 21, 2006

Version 8 : July 1, 2007

Version 9 : November 12, 2007

Version 10 : April 25, 2008

Version 11 : October 7, 2008

Version 12 : May 11, 2009

Version 13 : September 29, 2009

Kowa Company, Ltd.

Protection of confidentiality

This document contains information provided only to those who are directly involved in this clinical study. Prior agreement of Kowa Company Ltd. must be obtained before the content of this document is published or disclosed to a third party.

Definition of terms

Unless otherwise defined, the following definitions shall apply to the terms used in the “Contents of the Good Clinical Practice (GCP) (Report of the Central Pharmaceutical Affairs Council)” dated March 13, 1997.

List of abbreviations

| Abbreviation | English | Comments |
| --- | --- | --- |
| HCC | hepatocellular carcinoma | hepatocellular carcinoma |
| HCV | hepatitis C virus | hepatitis C virus |
| HBs antigen | hepatitis B surface antigen | hepatitis B surface antigen |
| AFP | α-fetoprotein | α-fetoprotein |
| AFP-L_3_ | LCA-reactive α-fetoprotein isoform | α-fetoprotein lectin fraction |
| all-*trans*-RA | all-*trans*-retinoic acid | all-*trans*-retinoic acid |
| 9-*cis*-RA | 9-*cis*-retinoic acid | 9-*cis*-retinoic acid |
| BNP | Brain Natriuretic Peptide | brain natriuretic peptide |
| CT | computed tomography | computed tomography |
| dynamic CT | dynamic computed tomography | A technique by which contrast media are intravenously injected rapidly and CT images are obtained over time |
| CTA/CTAP | CT arteriography / CT during arterial  Portography | CT arteriography/CT during arterial Portography |
| DEN | Diethylnitrosamine | Diethylnitrosamine |
| DXA | Dual Energy X-Ray Absorptiometry | Dual Energy X-Ray Absorptiometry |
| DXR | doxorubicin hydrochloride | doxorubicin hydrochloride (Adriamycin) |
| ELISA | Enzyme-Linked Immunosorbent Assay | enzyme-linked immunosorbent assay |
| FT_３_ | free triiodothyronine | free triiodothyronine |
| FT_４_ | free thyroxine | free thyroxine |
| 5FU | 5-fluorouracil | 5-fluorouracil |
| HCG | human chorionic gonadotropin | human chorionic gonadotropin |
| hANP | human atrial natriuretic peptide | human atrial natriuretic peptide |
| MDCT | multidetector row CT | multidetector-row CT |
| 3'-MeDAB | 3'-Methyl-4-dimethylaminoazobenzene | 3'-Methyl-4-dimethylaminoazobenzene |
| MMC | mitomycin C | mitomycin C |
| MRI | magnetic resonance imaging | Magnetic resonance imaging |
| PEI | percutaneous ethanol injection | percutaneous ethanol injection |
| PIVKA-II | protein induced by vitamin K absence  or antagonist-II | Abnormal protein generated as a result of failure in biosynthesis of prothrombin, a blood coagulation factor II, in the liver |
| PMC | percutaneous microwave coagulation  therapy | percutaneous microwave coagulation therapy |
| PWV/ABI | Pulse Wave Velocity/  Ankle Brachial Index | pulse wave velocity ankle brachial index |
| Real-Time RT-PCR | Real-time reverse transcription-  polymerase chain reaction | real-time reverse transcription-  polymerase chain reaction |
| RFA | radio frequency ablation | radio frequency ablation |
| RNA | ribonucleic acid | ribonucleic acid |
| TAE/TACE | transarterial embolization/transarterial chemoembolization | transarterial embolization/transarterial chemoembolization |
| TGF-α | transforming growth factor-α | transforming growth factor-α |
| TSH | thyroid stimulating hormone | thyroid stimulating hormone |
| US | Ultrasonography | Ultrasonography |

Table of Contents

Summary of Study Plan 1-vii

[1. Study administrative structure 3](#__RefHeading___Toc255913024)

[1.1 Sponsor 3](#__RefHeading___Toc255913025)

[1.2 Study centers and investigators 3](#__RefHeading___Toc255913026)

[1.3 Facilities undertaking determination and analysis of gene expression profile 3](#__RefHeading___Toc255913027)

[1.4 Facility undertaking laboratory tests 3](#__RefHeading___Toc255913028)

[1.5 Emergency safety center (Emergency Contact for Nighttime and Public Holiday) 3](#__RefHeading___Toc255913029)

[2. Background information 3](#__RefHeading___Toc255913030)

[2.1 Study drug 3](#__RefHeading___Toc255913031)

[2.2 Development history 3](#__RefHeading___Toc255913032)

[2.3 Summary of clinically important findings 3](#__RefHeading___Toc255913033)

[2.4 Summary of known and potential risks and benefits for subjects 3](#__RefHeading___Toc255913034)

[2.5 Compliance with various standards 3](#__RefHeading___Toc255913035)

[3. Study objectives 3](#__RefHeading___Toc255913036)

[4. Study design 3](#__RefHeading___Toc255913037)

[4.1 Study design 3](#__RefHeading___Toc255913038)

[4.2 Evaluation parameters 3](#__RefHeading___Toc255913039)

[4.3 Blinding procedures and maintenance of blindness 3](#__RefHeading___Toc255913040)

[4.4 Administration method, doses and administration period 3](#__RefHeading___Toc255913041)

[4.5 Follow-up examination 3](#__RefHeading___Toc255913042)

[4.6 Expected period of subjects’ participation 3](#__RefHeading___Toc255913043)

[4.7 Amount of liver tissue samples collected and frequency of sample collection 3](#__RefHeading___Toc255913044)

[4.8 Amount of blood collected and the number of days of blood collection 3](#__RefHeading___Toc255913045)

[4.9 Amount of urine collected and frequency of urine sample collection 3](#__RefHeading___Toc255913046)

[5. Criteria for inclusion and exclusion of subjects 3](#__RefHeading___Toc255913047)

[5.1 Study subject population 3](#__RefHeading___Toc255913048)

[5.2 Inclusion criteria 3](#__RefHeading___Toc255913049)

[5.3 Exclusion criteria 3](#__RefHeading___Toc255913050)

[6. Study drug 3](#__RefHeading___Toc255913051)

[6.1 Name of study drug, components, contents, dosage form, etc. 3](#__RefHeading___Toc255913052)

[6.2 Packaging and labeling of the study drug 3](#__RefHeading___Toc255913053)

[6.3 Management of study drugs 3](#__RefHeading___Toc255913054)

[7. Examination and observation parameters 3](#__RefHeading___Toc255913055)

[7.1 Procedures of subject registration 3](#__RefHeading___Toc255913056)

[7.2 Preparation of subject screening name list, etc. 3](#__RefHeading___Toc255913057)

[7.3 Subject backgrounds 3](#__RefHeading___Toc255913058)

[7.4 Gene expression profile 3](#__RefHeading___Toc255913059)

[7.5 Safety 3](#__RefHeading___Toc255913060)

[7.6 Pharmacokinetics 3](#__RefHeading___Toc255913061)

[7.7 Exploratory biomarkers (TGF-α concentration in plasma) 3](#__RefHeading___Toc255913062)

[7.8 Acceptable range of deviations from the dates of examination, observation and evaluation 3](#__RefHeading___Toc255913063)

[8. Subjects’ treatment compliance and subject management 3](#__RefHeading___Toc255913064)

[8.1 Subjects’ treatment compliance 3](#__RefHeading___Toc255913065)

[8.2 Subject management 3](#__RefHeading___Toc255913066)

[8.3 Status of completion of the study 3](#__RefHeading___Toc255913067)

[9. Measures taken to cope with adverse events and reporting procedures 3](#__RefHeading___Toc255913068)

[9.1 Securing subjects’ safety 3](#__RefHeading___Toc255913069)

[9.2 Follow-up examination 3](#__RefHeading___Toc255913070)

[9.3 Reporting of adverse events 3](#__RefHeading___Toc255913071)

[9.4 Reporting to the regulatory authority 3](#__RefHeading___Toc255913072)

[10. Discontinuation criteria and procedures of discontinuation 3](#__RefHeading___Toc255913073)

[10.1 Discontinuation criteria 3](#__RefHeading___Toc255913074)

[10.2 Procedures of discontinuation 3](#__RefHeading___Toc255913075)

[11. Treatment for subjects 3](#__RefHeading___Toc255913076)

[12. Statistic analysis 3](#__RefHeading___Toc255913077)

[12.1 Tabulation of subject background 3](#__RefHeading___Toc255913078)

[12.2 Analysis of gene expression profile 3](#__RefHeading___Toc255913079)

[12.3 Safety analysis 3](#__RefHeading___Toc255913080)

[12.4 Pharmacokinetics analysis 3](#__RefHeading___Toc255913081)

[12.5 Analysis of TGF-α 3](#__RefHeading___Toc255913082)

[12.6 The number of subjects to be registered according to the plan 3](#__RefHeading___Toc255913083)

[12.7 Selection of the subjects to be analyzed and handling of missing data, excluded data and abnormal data 3](#__RefHeading___Toc255913084)

[12.8 Deviations from the initial statistic analysis plan 3](#__RefHeading___Toc255913085)

[13. Direct access to the source documents, etc. 3](#__RefHeading___Toc255913086)

[13.1 Identification of source documents 3](#__RefHeading___Toc255913087)

[13.2 Information directly recorded on case report forms to be regarded as source data 3](#__RefHeading___Toc255913088)

[13.3 Direct access to source documents 3](#__RefHeading___Toc255913089)

[14. Quality control and quality assurance of the study 3](#__RefHeading___Toc255913090)

[14.1 Quality control of the study 3](#__RefHeading___Toc255913091)

[14.2 Quality assurance of the study 3](#__RefHeading___Toc255913092)

[15. Ethics 3](#__RefHeading___Toc255913093)

[15.1 Approval of the Institutional Review Board 3](#__RefHeading___Toc255913094)

[15.2 Subjects’ informed consent 3](#__RefHeading___Toc255913095)

[15.3 Protection of subjects’ privacy 3](#__RefHeading___Toc255913096)

[16. Handling of data and storage of records 3](#__RefHeading___Toc255913097)

[16.1 Essential documents to be stored 3](#__RefHeading___Toc255913098)

[16.2 Responsibility for storing and storing period 3](#__RefHeading___Toc255913099)

[17. Payment and compensation 3](#__RefHeading___Toc255913100)

[17.1 Payment 3](#__RefHeading___Toc255913101)

[17.2 Compensation 3](#__RefHeading___Toc255913102)

[18. Arrangement regarding publication 3](#__RefHeading___Toc255913103)

[19. Study period 3](#__RefHeading___Toc255913104)

[20. Protocol agreement/compliance, deviations/changes and revisions 3](#__RefHeading___Toc255913105)

[20.1 Protocol agreement and compliance 3](#__RefHeading___Toc255913106)

[20.2 Protocol deviations, changes and revisions 3](#__RefHeading___Toc255913107)

[21. Procedures of completion and correction of case report forms 3](#__RefHeading___Toc255913108)

[21.1 Case report forms 3](#__RefHeading___Toc255913109)

[21.2 Precautions for completion of case report forms 3](#__RefHeading___Toc255913110)

[21.3 Procedures for correction of case report forms 3](#__RefHeading___Toc255913111)

[22. References 3](#__RefHeading___Toc255913112)

Table 1 88

Table 2 88

Table 3 89

Summary of Study Plan

**I Title**

Clinical pharmacology study of NIK-333

− Search for drug-response gene and examination of safety in patients who were completely cured of hepatitis C virus-positive hepatocellular carcinoma −

**II Study objectives**

This 8-week repeated administration study compares the NIK-333 doses of 300 mg/day and 600 mg/day in patients who were completely cured of hepatitis C virus (HCV)-positive hepatocellular carcinoma (HCC) by examining the changes in liver and peripheral blood gene expression profiles before and after administration of each dose and the drug concentrations in the liver and plasma. After the above test (liver biopsy), NIK-333 of 600 mg/day is administered to all subjects until Week 96 (88 weeks) to investigate safety, with a focus on the cardiovascular system, and the changes in plasma drug concentrations during long-term administration.

**III Study design**

**(1) Type of study**

Step I: Open-label, randomized, parallel-group, comparison study in which NIK-333 is administered repeatedly at the doses of 300 mg/day and 600 mg/day (Week 0 to Week 8)

Step II: Open-label study in which NIK-333 is administered at 600 mg/day alone (Week 8 to Week 96)

**(2) Evaluation parameters**

[1] Gene expression profile

Liver and peripheral blood gene expression profiles determined by the microarray method

[2] Safety

Adverse events, physiological tests, laboratory tests (hematology tests, blood biochemistry tests, urinalyses, blood pressure parameters), abdominal imaging diagnosis, endoscopy (esophagus/stomach), bone mineral analysis (DXA method), ECG, echocardiography, pulse wave examination (PWV/ABI), fundus examination

[3] Pharmacokinetics

Drug concentrations in the liver (unchanged NIK-333 and NIK-333 lipids form)

Drug concentrations in plasma (unchanged NIK-333, NIK-333 lipids form and NIK-333 metabolite)

Drug concentration in urine (unchanged NIK-333)

[4] Exploratory biomarker

TGF-α concentration in plasma (biomarker related to gene expression)

**IV Subjects, inclusion criteria and exclusion criteria**

**(1) Subjects**

Subjects in the study are patients who underwent a topical medical therapy or surgical resection after diagnosis of HCV-positive HCC and who subsequently were confirmed by CT of showing a complete cure of HCC.

**(2) Inclusion criteria**

Patients who meet the following criteria will be selected as the subjects.

[1] Patients with HCV-positive HCC who meet the following conditions before radical treatment

1) Patients diagnosed as having typical HCC on dynamic CT (MD) or CTA/CTAP

2) Patients with primary cancer or the first recurrence of primary HCC (recurrence after an interval of at least one year after treatment of the first primary HCC)

[2] Patients who received one of the following treatments

1) Topical medical therapy

Patients who have undergone local radical therapy of HCC mainly by radiofrequency ablation (RFA) (However, percutaneous ethanol injection (PEI) or percutaneous microwave coagulation therapy (PMC) in combination with RFA are allowed.)

2) Surgical resection

Patients who have undergone liver resection

[3] Patients in whom complete cure has been confirmed by the following methods

1) Topical medical therapy

The dynamic CT images taken from 8 weeks (56 days) to 12 weeks (84 days) after topical medical therapy show no findings suggestive of recurrence at the treatment site and other sites, and the investigator or the subinvestigator has confirmed complete cure.

2) Surgical resection

The dynamic CT images taken from 8 weeks (56 days) to 12 weeks (84 days) after liver resection show no findings suggestive of recurrence or residue, and the investigator or the subinvestigator has confirmed complete cure.

[4] Patients who are able to begin treatment with the study drug within 8 weeks (56 days) after the dynamic CT to confirm complete cure

[5] Patients confirmed of satisfying the following conditions based on the screening performed at subject registration

1） HCV-RNA is present in serum.

2） Grade A or B on Child-Pugh classification

| Score | | 1 | 2 | 3 |
| --- | --- | --- | --- | --- |
| Liver encephalopathy | | None | Mild | Occasional coma |
| Ascites | | None | Small amount | Moderate amount |
| Serum bilirubin (mg/dL) | Patients other than those mentioned below | <2 | 2-3 | 3< |
|  | Patients with biliary cirrhosis primary | <4 | 4-10 | 10< |
| Serum albumin (g/dL) | | 3.5< | 2.8-3.5 | <2.8 |
| Prothrombin (s: seconds), (%: activity value) | | <4，70%< | 4-6，40-70% | 6<，<40% |

Grade A:5-6 points Grade B: 7-9 points Grade C: 10-15 points

3) Platelet count of 50,000/µL or higher

[6] Patients of the age of 20 years or older at the time of informed consent

**(3) Exclusion criteria**

Patients who fall under any of the following criteria will be excluded.

[1] Patients positive for HBs antigen

[2] Patients showing portal infiltration of HCC on CT images

[3] Patients who have also undergone transcatheter arterial embolization therapy (TAE/TACE) in combination with the radical therapy

[4] Patients who have received other study drugs, anticancer drugs, interferons, or vitamin K_2_ (oral preparations) after radical therapy

[5] Patients who have hypertension as a complication, and whose blood pressure cannot be controlled by drug therapy (systolic blood pressure of 160 mmHg or higher or diastolic blood pressure of 100 mmHg or higher, as determined at subject registration)

[6] Patients who have a history of allergy to CT contrast media, and whose participation in this study is judged to be inappropriate by the investigator or the subinvestigator

[7] Patients whose hemoglobin content is less than 8.0 g/dL and whose prothrombin time (%: activity value) is less than 40%, patients who are taking drugs with a potential of inducing blood coagulation abnormality, and patients who are judged to be ineligible for liver biopsy by the investigator or the subinvestigator

[8] Patients with a history of total gastrectomy

[9] Patients with serious complications (serious renal disorder, cardiovascular disease, diabetes mellitus, autoimmune disease, asthma, etc.)

[10] Patients confirmed of having another malignant neoplasm or who had undergone a radical therapy within the last 5 years to treat another malignant neoplasm (however, this does not apply to endoscopic resection and resection of intraepithelial carcinoma)

[11] Patients who are pregnant, who have a possibility of being pregnant or who have a desire to become pregnant during the study period

[12] Lactating women

[13] Patients who have a history of allergy to retinoid-related substances (vitamin A, etc.) in the past

[14] Other patients deemed ineligible to participate in the study by the investigator or the subinvestigator

**V Study drug**

**(1) Chemical name**

(2*E*,4*E*,6*E*,10*E*)-3,7,11,15-Tetramethylhexadeca-2,4,6,10,14-pentaenoic acid

**(2) Drug product**

NIK-333 soft capsule (test drug)

Yellow-brown soft capsules containing 75 mg of NIK-333 in one capsule

**VI Dose, regimen and duration of treatment**

**(1) Doses and administration period**

**NIK-333 of 300 mg (2 capsules x twice/day) or 600 mg (4 capsules x twice/day)**

| Administration period | Step I (Week 0 to Week 8) | | Step II (Week 8 to Week 96) |
| --- | --- | --- | --- |
| Dose of NIK-333 | 300 mg group | 300mg  (2 capsules × twice/day) | 600 mg  (4 capsules × twice/day) |
|  | 600 mg group | 600 mg  (4 capsules × twice/day) |  |

(One capsule contains 75 mg of NIK-333.)

**(2) Regimen**

Orally administer the designated dose twice daily, after breakfast and after supper.

**VII Determination and analysis of gene expression profile**

RNA will be extracted from the liver tissues and peripheral blood samples obtained from liver biopsy performed before and at Week 8 after the start of administration of NIK-333 for determination and analysis of gene expression profiles using KANAZAWA Liver Chip 10K (cDNA microarray) and AceGene®Premium (oligonucleotide microarray).

The gene suspected to be a drug response gene based on the above-mentioned analysis will be subjected to additional analysis by the Real-Time RT- PCR method.

**VIII Concomitant therapy**

**(1)** Prohibited concomitant drugs and prohibited concomitant food

|  | Drug classification | Generic name | Brand name | |
| --- | --- | --- | --- | --- |
| 1 | Other study drugs | − | − | |
| 2 | Anticancer drugs | All anticancer drugs including 5FU, MMC, DXR and tretinoin | | |
| 3 | Natural interferons | Interferon α | OIF, Sumiferon | |
|  |  | Interferon β | IFN β, Feron | |
| 4 | Genetically-modified interferons | Peginterferon-α-2a | | Pegasys |
|  |  | Interferon α-2b | Intron A | |
|  |  | Peginterferon-α-2b | Pegintron | |
|  |  | Interferon alfacon-1 | Advaferon | |
|  |  | Interferon β-1a | Avonex | |
|  |  | Interferon β-1b | Betaferon | |
| 5 | Vitamin K_2_ | Menatetrenone | Glakay, Kaytwo | |
| 6 | Retinoid | Etretinate | Tigason | |
| 7 | Vitamin A | Cod-liver oil | Cod-liver oil | |
|  |  | Retinol palmitate | Chocola A | |
| 8 | Antiviral drugs | Ribavirin | Rebetol, Copegus | |
| 9 | Supplements containing vitamin A (Lyc-O-Mato Power E, etc.) (health food) | | | |
| 10 | Supplements containing vitamin K (health food) | | | |

(2) Caution-required concomitant drugs

|  | Drug classification | Generic name | Brand name |
| --- | --- | --- | --- |
| 1 | Ursodeoxycholic acid | Ursodeoxycholic acid | Ubiron, Ursamic, Urso, Urdeston, Urdex, Urdenacin, Gokumisin, Shikichol, Braue, Precoat, Reptor |
| 2 | Phenytoin | Phenytoin-sodium | Aleviatin, Hydantol, Phenytoin |
| 3 | Tetracyclines antibiotics | Tetracycline hydrochloride | Achromycin, Achromycin V |
|  |  | Demethylchlortetracycline hydrochloride | Ledermycin |
|  |  | Doxycycline hydrochloride | Paldomycin, Vibramycin, Piperamycin, Rasenamycin |
|  |  | Minocycline hydrochloride | Minocyclin hydrochloride, Coupelacin, Namimycin, Pardoclin, Minotowa, Minopen, Minomycin |
| 4 | Drugs interfering with glucuronate conjugation | Sodium valproate | Epirenat, Sanoten, Cebotval, Selenica R, Cereb, Depakene, Depakene R, Hyserenin, Baldeken R, Sodium Valproate, Valpram R, Valerin |
|  |  | Lorazepam | Azrogen, U-Pan, Rocosgen, Lorazepam, Wypax |

**IX Expected sample size**

12 subjects

Subjects will be divided into the following two groups in Step I (Week 0 to Week 8).

NIK-333 300 mg group: 6 subjects

NIK-333 600 mg group: 6 subjects

All subjects will received 600 mg/day in Step II (Week 8 to Week 96).

**X Study period**

September 2005 to December 2009

XI Schedule of main examinations, observations and evaluations

| Timing (week)  Parameter | | Registration (within 56 days before) | During study drug administration period | | | | | | | At the time of follow-up examination (Week 12 after completion of study drug administration) | At the time of study discontinuation |
| --- | --- | --- | --- | --- | --- | --- | --- | --- | --- | --- | --- |
|  |  |  | Start of administration (within 14 days before) | Week 4, 28, 52, 76 | Week 8, 32, 56, 80 | Week 12, 36, 60, 84 | Week 16, 40, 64, 88 | Week 20, 44, 68, 92 | Week 24, 48, 72, 96 |  |  |
| Study drug administration | | − |  | | | | | | | − | − |
| Examination of compliance status | | − | − | ● | ● | ● | ● | ● | ● | − | − |
| Gene expression profile | | − | ⦿ | − | ⦿ | − | − | − | − | − | − |
| TGF-α | | − | ⦿ | − | ⦿ | ● | − | − | ● | − | ✰ |
| Drug concentration in the liver | | − | ⦿ | − | ⦿ | − | − | − | − | − | − |
| Drug concentration in plasma | | − | ⦿ | − | ⦿ | − | − | − | ● | ● | − |
| Drug concentration in urine | | − | ⦿ | − | ⦿ | − | − | − | − | − | − |
| Abdominal imaging diagnosis | | ● | − | − | − | ● | − | − | ● | ● | ✰ |
| Medical examination | | ● | ● | ● | ● | ● | ● | ● | ● | ● | ✰ |
| Vital signs, etc. | | ● | ● | ● | ● | ● | ● | ● | ● | ● | ✰ |
| Child-Pugh classification | Encephalopathy / ascites | ● | − | − | − | − | − | − | − | − | − |
|  | T-Bil,ALB,PT | ● | − | − | − | − | − | − | − | − | − |
| HBs antigen | | ● | − | − | − | − | − | − | − | − | − |
| HCG tests (premenopausal women only) | | ● | − | − | − | − | − | − | ● | − | − |
| Hematology tests  (platelet count, hemoglobin level) | | ● | − | − | − | − | − | − | − | − | − |
| Hematology tests | | − | ● | ● | ● | ● | ● | ● | ● | ● | ✰ |
| Blood biochemistry tests | | − | ● | ● | ● | ● | ● | ● | ● | ● | ✰ |
| Urinary tests (fresh urine) | | − | ● | ● | ● | ● | ● | ● | ● | ● | ✰ |
| Blood pressure parameters | | − | ● | − | − | − | − | − | ★ | − | ✰ |
| HCV-RNA (quantitative) | | ● | − | − | − | − | − | − | ★ | − | ✰ |
| HCV-RNA (genotype analysis) | | − | ● | − | − | − | − | − | − | − | − |
| Tumor markers | | − | ● | − | − | ● | − | − | ● | ● | ✰ |
| Fibrosis marker | | − | ● | − | − | − | − | − | ★ | − | ✰ |
| Bone densitometry (DXA，lumbar vertebrae) | | − | ○ | − | − | − | − | − | ★ | − | ✰ |
| Endoscopy | | − | ○ | − | − | − | − | − | ★ | − | ✰ |
| ECG | | − | ○ | − | − | − | − | − | ● | − | ✰ |
| Echocardiography | | − | ○ | − | − | − | − | − | ★ | − | ✰ |
| Pulse wave examination | | − | ○ | − | − | − | − | − | ★ | − | ✰ |
| Fundus examination | | − | ○ | − | − | − | − | − | ★ | − | ✰ |
| Adverse events | | − |  | | | | | | | |  |

⦿:The day before the start of administration and the day of evaluation at Week 8

○: Examination will be conducted during the period between the end of radical therapy and the start of study drug administration.

★: Examination will be conducted at 48-week intervals.

✰: Examination will be conducted upon discontinuation of the study if possible.

Note 1: The acceptable range of deviation from the scheduled days of examination, observation and evaluation during the study period is +28 days for gene expression profile (Week 8), TGF-α (Week 8), and liver, plasma, and urinary pharmacokinetics (Week 8, respectively); ±35 days for abdominal imaging diagnosis, HCV-RNA, HCG tests, tumor markers, fibrosis markers, bone densitometry, endoscopy, ECG, echocardiography, pulse wave examination, and fundus examination; and ±7 days for other parameters.

Note 2: Subjects need to be hospitalized on the day of the start of administration and on the day of evaluation at Week 8. Liver biopsy will be performed and blood samples and accumulated urine samples will be collected on the day before the start of administration, with administration of the study drug to start 24 hours after the operation. On the day of evaluation at Week 8, subjects will visit the hospital without taking the study drug, and undergo liver biopsy 4 hours after administration. Blood samples will be collected 4 and 8 hours after administration. Urine samples will be accumulated for 24 hours after administration.

Note 3: Blood pressure parameters will be assessed on the day of the start of administration and at Week 48 and Week 96 of administration. If the systolic blood pressure exceeds 140 mmHg or the diastolic blood pressure exceeds 90 mmHg in two or more consecutive tests, the parameters will be determined on the second or subsequent tests. Determination of creatinine clearance, one of the blood pressure parameters, under increased blood pressure will be conducted to the extent possible. Creatinine clearance will be determined using urine samples collected at home at the timings other than the day of the start of administration (i.e., Week 48 and Week 96 of administration, and examination of blood pressure parameters in the presence of increased blood pressure).

Note 4: See Table 1, Table 2 and Table 3 at the end of this document for details about the contents of the tests.

# 1. Study administrative structure

## 1.1 Sponsor

(1) Sponsor

Kowa Company, Ltd.

3-6-29, Nishiki, Naka Ward, Nagoya City, Aichi Prefecture, 460-8625

(2) Sponsor’s study manager

Kazuhide Shimada, Director, Clinical Development Dept. II, Pharmaceutical Division Kowa Company, Ltd.

3-4-14, Nihonbashi-honcho, Chuo Ward, Tokyo, 103-8433

[Main responsibilities]

Responsible for the entire study process, authorized to sign the protocol and study report as a representative of the sponsor, and responsible for global supervision of the study.

(3) Medical experts

Kiwamu Okita

Director, Shimonoseki Kosei Hospital

3-3-8, Kamishinchicho, Shomonoseki City, Yamaguchi Prefecture, 750-0061

Tel. 0832-31-5811 (Main Switchboard)

[Main responsibilities]

[1] To give medical advice on preparation or revision of the study protocol, case report forms, informed consent forms, written information and investigator’s brochure

[2] To give medical advice on adverse events encountered

[3] To give medical advice on discontinuation or suspension of the clinical study or discontinuation of development of the study drug

[4] To give medical advice on case handling, results of analysis obtained after termination of the study, and evaluation of these results

[5] To give medical advice on preparation of the study report and to affix his/her signature and seal on the study report

[6] To give advice on other medical affairs related to the clinical study

(4) Monitors

Responsible person: Tetsuro Sano

Manager, Clinical Development 2, Clinical Development Dept. II, Pharmaceutical Division, Kowa Company, Ltd.

Persons in charge: Ken Uesugi, Masashi Yano, Takanori Meikari, Yuko Saito, Toru Akiba, Kozo Nakamura, Asako Komatsu, Noriaki Kitajima

Clinical Development Dept. II, Pharmaceutical Division, Kowa Company, Ltd.

3-4-14, Nihonbashi-honcho, Chuo Ward, Tokyo, 103-8433

Tel. 03-3279-7856 Fax. 03-3279-7869

(5) Statistic Analysis Department and Data Management Department

1) Statistic Analysis Department

Responsible person: Hideki Suganami

Manager, Biostatistics, Clinical Data Science Dept., Pharmaceutical Division, Kowa Co, Ltd.

3-4-14, Nihonbashi-honcho, Chuo Ward, Tokyo, 103-8433

Tel. 03-3279-7463 Fax. 03-3279-7869

2) Data Management Department

Responsible person: Satoru Kokubun

Manager, Clinical Data Management, Clinical Sciense Dept., Pharmaceutical Division, Kowa Co, Ltd.

3-4-14, Nihonbashi-honcho, Chuo Ward, Tokyo, 103-8433

Tel. 03-3279-7859 Fax. 03-3279-7869

(6) Auditors

Responsible person: Yoshimasa Nagasaka

Manager, GCP / GLP Audit Dept, Pharmaceutical Division, Kowa Co, Ltd.

Person in Charge: Akira Kioue, Kazumi Sakai, Junya Ikuta, Mihoko Shirota, Yoshikazu Matsumura

Compliance & Quality Assurance, Pharmaceutical Division, Kowa Co, Ltd.

3-4-14, Nihonbashi Honcho, Chuo Ward, Tokyo, 103-8433

Tel. 03-3279-7367 Fax. 03-3279-7414

## 1.2 Study centers and investigators

Kanazawa University Hospital, Department of Gastroenterological Medicine

Investigator: Tatsuya Yamashita

13-1, Takara Machi, Kanazawa City, Ishikawa Prefecture, 920-8641

Tel. 076-265-2235 Fax. 076-265-4250

[Main responsibilities]

[1] To examine the study protocol (draft) and the case report forms (draft)

[2] To agree to and comply with the contents of the study protocol and the case report forms

[3] To prepare and revise informed consent forms and written information

[4] To select subjects

[5] To obtain informed consent from subjects

[6] To provide appropriate medical care to the subjects

[7] To submit the documents that require review by the Institutional Review Board

[8] To start, conduct and discontinue the clinical study according to the directions and decision of the head of the study center

[9] To make sure that the study drug is properly used according to the study protocol

[10] To report any protocol deviations to the head of the study center and the sponsor

[11] To complete case report forms and submit them to the sponsor

[12] To report any serious adverse events and the likes that emerged during the study to the sponsor and the head of the study center

[13] To notify the subjects of any discontinuation or suspension of the study and to provide appropriate treatment

[14] To report completion of the study and a summary of study results to the head of the study center

[15] To store essential documents related to the conduct of the study, which are to be kept by the investigator

## 1.3 Facilities undertaking determination and analysis of gene expression profile

(1) Kanazawa University, Graduate School of Medical Science, Division of Disease Control and Homeostasis

Study Investigator: Masao Honda

13-1, Takara Machi, Kanazawa City, Ishikawa Prefecture, 920-8641

Tel. 076-265-2243 Fax. 076-265-4250

[Main responsibilities]

[1] To determine gene expression profile in liver biopsy tissue samples

[2] To send reports of the determination results to the sponsor

[3] To analyze the results of profile determination in the liver biopsy and peripheral blood samples (search for drug response gene)

[4] To prepare the plan and report of analysis and send them to the sponsor

[5] To store records and samples, including source data

(2) DNA Chip Research Inc.

Responsible Contractor: Kenichi Matsubara

1-1-43, Suehiro Cho, Tsurumi Ward, Yokohama City, Kanagawa Prefecture, 230-0045

Tel. 045-500-5225 Fax. 045-500-5229

[Main responsibilities]

[1] To determine gene expression profile in peripheral blood samples

[2] To send reports of the determination results to the sponsor

[3] To store records and samples, including source data

## 1.4 Facility undertaking laboratory tests

(1) Facility undertaking determination of drug concentrations and TGF-α

Sumika Chemical Analysis Service, Ltd., Pharmaceutica Business Division, Pharmaceutical Analysis Laboratory

Responsible person: Masayuki Izutsu

3-1-135, Kasugade-Naka, Konohana Ward, Osaka City, Osaka, 554-0022

Tel. 06-6466-5245 Fax. 06-6466-5479

[Main responsibilities]

[1] To determine drug concentrations in the liver, plasma and urine

[2] To determine the concentration of TGF-α in plasma

[3] To send reports of the determination results to the sponsor

[4] To store records and specimens, including source data

(2) Facilities undertaking laboratory tests

1) Facility undertaking works

SRL Medisearch, Inc.

Person responsible for undertaking of the works: Masayuki Nagasaki

1-17-1, Nishi Shinjuku, Shinjuku Ward, Tokyo, 160-0023

Tel. 03-5324-2601 Fax. 03-5324-3507

2) Facility undertaking laboratory tests

SRL, Inc.

Person responsible for undertaking of the works: Yoji Hirabayashi

51, Komiya Machi, Hachijoji City, Tokyo, 192-8535

Tel. 042-648-4010 Fax. 042-648-4058

[Main responsibilities]

[1] To collect blood specimens from each study center

[2] To carry out measurements of laboratory test parameters

[3] To send repots of measurement results to the sponsor and the study centers

[4] To store records and specimens, including source data

## 1.5 Emergency safety center (Emergency Contact for Nighttime and Public Holiday)

Bellsystem 24, Inc.

Operation Division, Medical Drug associated Department, Head Office of Medical Drug associated Service

Person in Charge: Ryo Watabe, Head of Division

2-16-8, Minami Ikebukuro, Toshima Ward, Tokyo, 171-0022

Tel. 0120-490-773 Fax. 0120-490-774

Tel. 03-5954-0024 Fax. 03-5951-9407

[Main responsibilities]

To notify the sponsor upon receiving a report of the occurrence of any adverse events from the study center

# 2. Background information

## 2.1 Study drug

In this study, yellow-brown soft capsules NIK-333 containing 75 mg of (2*E*,4*E*,6*E*,10*E*)-3,7,11,15-Tetramethylhexadeca-2,4,6,10,14-pentaenoic acid will be used.

## 2.2 Development history

(1) Background of target disease

The death toll from malignant neoplasms of the liver and intrahepatic bile duct was 34,637 in 2002 in Japan, and malignant neoplasms of the liver and intrahepatic bile duct are ranked third among various types of malignant neoplasms in terms of the number of deaths (third place in men and fourth place in women).^1)^ Moreover, more than 90% of the cases of primary HCC in Japan are believed to be mainly attributable to continuous infection with hepatitis B virus (HBV) or hepatitis C virus (HCV), and the incidence of and the number of deaths from HCC is on the rise every year.^2)^

In Japan, 15.5% of the patient with HCC are reported to be positive for HBs antigen, and 71.8% are reported to be positive for HCV antibody.^3)^ In particular, HCV-positive HCC is a disease with much poorer prognosis than HBV-positive HCC, with recurrence observed in 24%, 76% and 92% of the patients within, respectively, 1 year, 3 years and 5 years after treatment of the initial onset of HCC.^4)^ Therefore, in addition to early detection and early treatment of HCV-positive HCC, prevention of recurrence becomes an important issue for the future. In the meantime, antiviral therapy using an agent such as interferons have been reported to be effective in some cases as a means to prevent recurrent and new HCC caused by hepatitis virus infection,^5-9)^ but no treatment method targeting prevention of recurrent HCC has been established to date.

(2) Information regarding the study drug

[1] Development history

Under such circumstances, the concept of chemoprevention^10)^ using retinoid has been proposed as a means to delay or prevent recurrence after treatment of HCC. Muto et al. in 1980 discovered (2E, 4E, 6E, 10E) - 3, 7, 11, 15 - Tetramethylhexadeca - 2, 4, 6, 10, 14 - pentaenoic acid, a synthetic polyprenoic acid exhibiting an affinity to intracellular retinoic acid-binding protein and a retinoid-like action.^11)^ Originally, it was developed under the name of E-5166 by Eisai as a drug for treating skin diseases.^12)^ Subsequently, Muto et al. orally administered this compound (600 mg/day, b.i.d.) for one year to patients who had received treatment for HCC, and reported that this compound showed good safety, inhibited recurrent HCC,^13)^ and improved the survival rate of the patients.^14)^ On the basis of these results, Nikken Chemicals Co., Ltd. (present Kowa Pharmaceutical Co., Ltd.) started development of this compound with the code number of NIK-333 in 1997. Kowa Company Ltd., which has taken over the research and development projects of Kowa Pharmaceutical Co., Ltd. as a result of corporate division, began working on the development of this compound in July 2007.

[2] Non-clinical studies

1) Pharmacological actions

NIK-333 exhibits a transcriptional action via the retinoic acid receptor (RAR) and retinoid X receptor (RXR), a differentiation-inducing action against HL-60 cells (human acute promyelocytic leukemia-derived cells), and retinoid-like actions. Moreover, NIK-333 induced apoptosis in well-differentiated human hepatoma-derived cells, HuH-7, although such an action was not seen for all-trans-RA and 9-cis-RA. It also significantly inhibited rat hepatocarcinogenesis by 3'-MeDAB and DEN^15)^. The mechanisms of hepatocarcinogenesis hypothesized to date include a possibility that, when hepatitis persists and necrosis and growth of hepatocytes are repeated, mutation is induced and the cells grow without being eliminated by apoptosis, thereby leading to carcinogenesis.^16)^ In contrast, NIK-333 is presumed to inhibit hepatocarcinogenesis on the basis of the following mechanisms: (1) elimination of preneoplastic cells or unapparent cancer cells in the liver by the apoptosis-inducing action, and (2) the antitumor effect and the carcinogenesis-inhibiting effect brought about by the differentiation-inducing action. Moreover, NIK-333 has recently been found to have an action to inhibit genetic transformation and an action to inhibit growth,^15）^ and these actions are considered to be involved in the cancer-inhibiting effect and the anti-promotion effect.

2) Information regarding gene expression

Reported effects of NIK-333 on gene expression include promotion of cell-cycle controlling protein p21 through RAR-β activation in human hepatoma-derived cells, inhibition of cell growth by inhibition of cyclin D,^17)^ and induction of apoptosis via the inhibition of TGF-α^18)^.

Moreover, the inhibitions of TGF-α expression and hepatocarcinogenesis have been reported in the rat liver cancer model.^15)^ In evaluating the usefulness of NIK-333 in the future, it is important to investigate whether the changes of gene expression observed in these basic studies are also seen in clinical settings.

[3] Clinical studies

1） Safety

To examine the safety, tolerability and pharmacokinetics of NIK-333, phase I studies (study of the effect of food, single administration study and repeated administration study) were conducted in patients who received treatment for HCC, for whom NIK-333 is expected to be indicated. Concerning tolerability in the 48-week repeated administration study, increased blood pressure was seen as an adverse drug reaction at 900 mg/day, but no serious or significant adverse drug reactions were observed at 300 and 600 mg/day. However, since increased blood pressure was observed in the group treated with 900 mg, a possibility cannot be ruled out that the same event may occur during administration of 600 mg for more than 48 weeks, the period which could be examined in this study. Furthermore, when abnormal variations in protein urine and urinary occult blood are also taken into consideration, it is deemed necessary to carefully cope with the occurrence of adverse drug reactions, mainly in the cardiovascular and urinary systems, during the administration period of any future long-term studies at daily doses of 300 mg and 600 mg.

2） Pharmacokinetics

In the phase I studies, the pharmacokinetic parameters (AUC_0-24hr_ and Cmax) of unchanged NIK-333 in plasma were confirmed to exhibit linearity in single and 48-week repeated administrations of 300 mg, 600 mg and 900 mg. Since almost no unchanged NIK-333 and NIK-333 lipids form were seen in plasma at Week 48 of administration and Week 12 and Week 24 after the end of administration, the drug was not considered to be accumulated in the body during repeated administration.

Excretion of unchanged NIK-333 in urine was not seen after single administration.

(3) Conclusions

On the basis of such backgrounds, the phase II/III studies of NIK-333 were started in February 2005 to examine the effect of NIK-333 to inhibit recurrence of HCC. The present clinical pharmacology study has been planned to investigate the gene expression profile in the liver and peripheral blood before and after 8-week administration of 300 mg/day or 600 mg/day of NIK-333 and to examine its relationship with the drug concentrations in the liver and plasma in patients who were completely cured of HCC, for whom the drug is expected to be indicated in the future, with the goal of identifying the gene or the gene cluster whose response can explain the above-described pharmacological actions of NIK-333. After this, all subjects will continue to receive NIK-333 at 600 mg/day only for up to 96 weeks, and the safety and plasma drug concentrations will be examined.

## 2.3 Summary of clinically important findings

(1) Non-clinical studies

[1] Pharmacological actions

1) Data on effectiveness

NIK-333 exhibited a transcriptional action via the retinoid receptor in *in vitro* studies, and the EC_30_ was RAR α: >10 µmol/L, β: 1.07 µmol/L, γ: >10 µmol/L, RXRα: 1.18µmol/L, β: 0.484 µmol/L, and γ: 2.43 µmol/L. NIK-333 also showed a differentiation-inducing action against HL-60 cells (human acute promyelocytic leukemia-derived cells) with an EC_50_ of 4.29 µmol/L. NIK-333 decreased the viable cell count of HuH-7 cells (human hepatoma-derived cells) in a concentration-dependent manner at 1 µmol/L or higher concentrations with an IC_50_ of 1.91 µmol/L. Moreover, determination of the DNA-histone complex level in cytoplasms and morphological evaluation of cells showed that NIK-333 at the concentration of 5 µmol/L increased the DNA-histone complex level in cytoplasms and induced both apoptosis body formation and intranuclear chromatin condensation. When cytotoxicity was tested using human hepatoma-derived cell cells and human normal liver-derived cells, NIK-333 showed IC_50_s of 0.220 µmol/L against HuH-7 cells and 24.6 µmol/L against Hc cells (human normal liver-derived cells), indicating that it is about 100 times more sensitive against HuH-7 cells. Furthermore, NIK-333 significantly inhibited 3'-MeDAB-induced or DEN-induced^15)^ rat hepatocarcinogenesis after oral administration at 40 mg/kg or higher doses. After a dose of 40 mg/kg, which exhibited a carcinogenesis-inhibiting action in both models, the NIK-333 concentration peaked at 0.3 to 0.4 µmol/L (93 to 134 ng/mL) in the plasma and at 90 to 548 ng/g in the liver. Therefore, after administration of 40 mg/kg, the NIK-333 concentration in liver tissues is believed to have reached both the IC_50_ for the toxic action against the human hepatoma cell line HuH-7 confirmed in an *in vitro* study (0.220 µmol/L, 66.5 ng/mL) and the IC_50_ for the apoptosis-inducing action (1.91 µmol/L, 578 ng/mL). These results suggested that NIK-333 has a retinoid-like action, an apoptosis-inducing action against liver cancer cells, and a hepatocarcinogenesis-inhibiting action.

2) Data on general pharmacology

NIK-333 at oral doses of 300 mg/kg or more increased the water content in the colon and induced diarrhea in mice. In a test of the spontaneous movement of extracted rabbit ileum, NIK-333 increased the degree of contraction only at the concentration of 100 µmol/L.

3) Data on safety pharmacology

In a dog 13-week repeated-dose study, NIK-333 at oral doses of 25 and 100 mg/kg had no effects on blood pressures (systolic blood pressure, diastolic blood pressure, and mean blood pressure), heart rate and ECG (PQ interval, QRS duration, QT interval, and QTc) in dogs.

[2] Toxicity

1) Single dose toxicity

The minimal lethal dose after single oral administration was higher than 2000 mg/kg in both rats (male and female) and dogs (male). Signs of toxicity observed include transient inhibition of weight gain and decreased food consumption in rats, and abnormal stools (soft stools, watery stools, mucous stools, and mucous stools containing blood) and vomiting in dogs.

2) Repeated dose toxicity

The no-observed-adverse-effect level for repeated oral administration was 30 and 10 mg/kg/day, respectively, after 4 weeks and 26 weeks of administration in rats, and 25, 30 and 45 mg/kg/day, respectively, after 4, 13 and 52 weeks of administration in dogs. Major toxicological changes in rats included anaemia, decreased albumin, increased alkaline phosphatase, cholesterol and phospholipid, erosion, edema and squamous epithelial growth in the anterior stomach, osteoporosis, and bone fractures. Major toxicological changes in dogs included abnormal stools (soft stools, watery stools, mucous stools, and contamination of stools with blood), redness and erosion of the skin and mucosa, and decreased albumin.

In toxicokinetic studies, the Cmax and AUC_0-24 hr_ of unchanged NIK-333 and a metabolite of NIK-333 lipids form increased in a near dose-dependent manner in rats and dogs, and all of these parameters except the Cmax of unchanged NIK-333 showed near dose-dependent increases in rabbits. Moreover, a near-steady state was reached within 4 weeks after the start of administration at the doses of 100 mg/kg/day or less in rabbits and dogs. In rabbits, unchanged NIK-333 decreased during a 2-week repeated administration, but there was no effect on NIK-333 lipids form. In all animal species studied, the plasma concentration of NIK-333 lipids form was higher than unchanged NIK-333. The plasma concentrations of unchanged NIK-333 were higher in dogs and rabbits than in rats, while the plasma concentrations of NIK-333 lipids form were comparable in rats, dogs and rabbits.

In a 13-week repeated dose study in mice conducted as a follow-up toxicokinetic study after the carcinogenicity study, the systemic exposure of unchanged NIK-333 in plasma at 40 mg/kg or higher doses, at which angiosarcoma was observed, was estimated to be 0.068 µg/mL or more (Cmax) and 0.46 µg·hr/mL or more (AUC_0-24hr_) on the basis of the results on Week 7, when a steady state is assumed to have been reached in these animals.

3) Reproductive and developmental toxicity

In a study of fertility and early embryonic development until implantation in rats, effects on reproductive functions were not observed in male rats. In female rats, an increased incidence of abnormal estrus cycle was observed at the dose of 100 mg/kg/day, but there were no effects of NIK-333 administration on copulation capability and fertility. Therefore, the no-observed-adverse-effect level for reproductive functions was 100 mg/kg/day in male rats and 50 mg/kg/day in female rats.

In a study of prenatal and postnatal development and maternal functions in rats, there were no effects on the maintenance of pregnancy in dams (F_0_). Effects observed on the offspring (F_1_) included external abnormalities and weight-gain inhibition at 100 mg/kg/day. Therefore, the no-observed-adverse-effect level for the maintenance of maternal pregnancy was 100 mg/kg/day, and the no-observed-adverse-effect level for the offspring was 50 mg/kg/day.

In a study of embryonic and fetal development in rats and rabbits, teratogenicity was observed at the dose of 100 mg/kg/day or more in rats and at 270 mg/kg/day in rabbits. Therefore, the no-observed-adverse-effect level for fetal development and growth was 30 mg/kg/day in rats and 90 mg/kg/day in rabbits. In rabbits, miscarriage was seen in the dams treated with 270 mg/kg/day, and the no-observed-adverse-effect level for maintenance of pregnancy was 90 mg/kg/day.

4) Mutagenicity

The results of the reverse mutation study, chromosomal aberration study, and micronucleus study were all negative.

5) Carcinogenicity

The incidence of angiosarcoma increased in the mice that received a 78-week oral administration at 40 mg/kg/day or a higher dose, and squamous cell tumors in the anterior stomach was induced in the rats that received a 104-week oral administration at 20 mg/kg/day or higher.

6) Antigenicity

The antigenicity studies in Guinea pigs and the mouse-rat system were all negative.

[3] Pharmacokinetics

1) Absorption

After single oral administration of NIK-333 in rats and dogs, the plasma concentration reached the peak within 2 hours after administration and decreased with half-lives of 1.1 to 2.1 hours and 1.4 to 7.4 hours, respectively. The Cmax and AUC_0-∞_ were 21 to 446 ng/mL and 84 to 2811 ng·hr/mL, respectively, in rats (8 to 200 mg/kg), and 77 to 1014 ng/mL and 91 to 3617 ng·hr/mL, respectively, in dogs (1.6 to 34 mg/kg), exhibiting dose-dependent increases. Bioavailability was 16 to 20% and 15 to 41% in rats and dogs, respectively.

2) Distribution

After single oral administration of ^14^C-NIK-333 in rats, the radioactivity concentration in tissues reached the peak within about 8 hours after administration, and concentrations 3.51 to 5.83 times higher than the plasma concentration were observed in the Harderian gland, liver and brown fat. At 168 hours after administration, the concentration decreased to 3 to 17% of the peak local concentration in blood, prostate gland, stomach, spleen, submandibular gland, small intestines, bone marrow, bladder, liver, Harderian gland and plasma. At other sites, a radioactivity concentration of 20 to 84% of the peak local concentration was seen, suggesting a slow clearance.

The plasma protein binding rates of NIK-333 in *in vitro* studies were 95% or higher in rats, dogs and humans at concentrations ranging from 20 to 2000 ng/mL, with albumin as the major binding protein. Binding to albumin was not affected by diazepam, digitoxin, warfarin and bilirubin.

3) Metabolism

In a 7-day repeated oral dose study in rats, NIK-333 at 40 mg/kg/day exhibited neither enzyme-inducing nor inhibitory effects on drug-metabolizing enzymes. At 200 mg/kg/day, significant increases were seen in the cytochrome P450 content, cytochrome b5 content, aniline hydroxylase activity, 7-ethoxycoumarin O-deethylase activity and NADPH cytochrome c reductase activity.

NIK-333 is presumed to be metabolized by being taken into neutral lipid as a constituent fatty acid, as well as by glucuronate conjugation, reduction of conjugated double bond, shortening of fatty chain by α- and β-oxidation, and ω-hydroxylation of methyl end. CYP2C8, CYP2C9*1, CYP2C9*2, CYP2C19, CYP4F2 and CYP4F3B have been found to be involved in ω-hydroxylation.

4) Excretion

The radioactivity excretion rates in urine, feces and expired air at 168 hours after single oral administration of 40 mg/kg of ^14^C-NIK-333 were 16.3, 37.8 and 35.1%, respectively, in rats, and 11.3, 57.1 and 15.0%, respectively, in dogs.

5) Drug interactions

In dogs, the plasma concentrations of NIK-333 and NIK-333 lipids form after the use of NIK-333 in combination with ursodeoxycholic acid were, respectively, 1.8 and 1.6 times (Cmax) and 1.4 and 1.2 times (AUC_0-t_) as compared with the group without the concomitant use of ursodeoxycholic acid.

(2) Clinical studies

Effects of food, single administration and repeated administration were investigated in phase I clinical studies conducted beginning from September 10, 2001 at the National Cancer Center Hospital in subjects who had received treatment for HCC to examine the method of drug administration, tolerability and pharmacokinetics. Follow-up examination of the bone was conducted at Week 12 and Week 24 after completion of the repeated dose study to confirm the elimination phase of the drug concentration.

In this study, the phase was divided into three levels according to the daily dose as shown in the table below, and the drug was administered by the dose escalation method starting with the lower dose.

Dose at each level

| Level | Dose of NIK-333 | |
| --- | --- | --- |
|  | Step I | Step II |
| 1 | 300 mg (4 capsules) | 150 mg × 2/day (2 capsules × 2/day) |
| 2 | 600 mg (8 capsules) | 300 mg × 2/day (4 capsules × 2/day) |
| 3 | 900 mg (12 capsules) | 450 mg × 2/day (6 capsules × 2/day) |

In Step I, 2 out of 12 subjects in the target sample size were to be treated with the placebo. Dose escalations from Level 1 to Level 2 and from Level 2 to Level 3 were to be decided on the basis of the tolerability results obtained by Week 24 of the repeated administration study. Each level consisted of two steps, i.e., Step I [study of the effect of food (Level 1), single administration study (Level 2 and 3)] and Step II, and the study of Step I and Step II was conducted as a series of study in the same subjects. The administration period in each step is shown in the table below.

Administration period in each step

| Step I | Study of the effect of food: 1 day × 2 (once each in fasted and fed conditions)  Single administration study: 1 day |
| --- | --- |
| Step II | Repeated administration study: 48 weeks |

The number of subjects enrolled in this clinical study at each level is shown in the table below. In Step II at Level 1, 2 subjects showed recurrent HCC during the period of transition from Step I to Step II, and were withdrawn after Step I. The total number of subjects enrolled at Level 3 was 9, which was under the target sample size (12 subjects). However, as explained later in “Repeated administration study and follow-up examination of the bone,” this was because increased blood pressure was seen in 1 subject who was suspended from the study and in 3 subjects who discontinued the study, and enrollment of new subjects was subsequently discontinued prematurely to ensure subjects’ safety.

Number of subjects enrolled at each level

| Level | Step I | | Step II |
| --- | --- | --- | --- |
|  | Placebo | Active drug |  |
| 1 | 2 | 10 | 10 |
| 2 | 2 | 10 | 12 |
| 3 | 1 | 8 | 9 |

[1] Study of the effect of food (Level 1)

Before the start of this study, the effect of food on pharmacokinetics of NIK-333 was investigated in Step I at Level 1 to determine the administration method for the subsequent single administration study and the repeated administration study. The subjects who used ursodeoxycholic acid, a prohibited concomitant drug on the day of study drug administration, were excluded from the data analysis.

The pharmacokinetic parameters of unchanged NIK-333 in plasma, which is the source of drug efficacy, were as follows: Cmax, 284.0 ng/mL and AUC_0-24hr_, 849.5 ng • hr/mL under fasted condition and Cmax, 316.1 ng/mL and AUC_0-24hr_, 841.3 ng • hr/mL under fed conditions. No significant differences were seen in the Cmax and AUC_0-24hr_ under the two conditions. The results of the analysis of variance of Cmax and Log (Cmax) of the unchanged NIK-333 in plasma between fed-fasted conditions were Cmax (p = 0.466) and Log (Cmax) (p = 0.982), showing no significant difference.

The pharmacokinetic parameters of a metabolite of NIK-333, NIK-333 lipids form, were Cmax, 295.4 ng/mL and AUC_0-24hr_, 3518.3 ng • hr/mL under fasted condition and Cmax, 694.1 ng/mL and AUC_0-24hr_, 6497.7 ng • hr/mL under fed conditions. The values under fed conditions were about twice higher than the values under fasted condition. Results of the analysis of variance of Cmax and Log (Cmax) of the lipids form in plasma between fed-fasted conditions showed significant differences in Cmax (p=0.011) and Log (Cmax) (p=0.001).

Urine samples were also assayed for NIK-333, and the level was below the limit of quantitation in all samples, indicating the absence of unchanged NIK-333 in urine after single administration at Level 1.

[2] Single administration study (Level 2 and 3)

1) Safety

Fifteen adverse events were observed in 8 subjects at Level 1, 5 events were observed in 4 subjects at Level 2, and 11 events were observed in 7 subjects at Level 3. At each level, no clinically problematic adverse events and abnormalities were observed in the parameters assessed, including vital signs, but the incidence of headache, which is a known adverse reaction to NIK-333, tended to increase in a dose-dependent manner.

List of adverse events after single administration

| Dose | Causal relationship | Events | Number of events | Total |
| --- | --- | --- | --- | --- |
| Placebo  (n=5) | None | Blood pressure increase, AST increase, T-Bil increase, Hb decreased, pyrexia | 1 | 9 |
|  |  | ALP increase, headache | 2 |  |
|  | Related | Feeling hot, LDH increase | 1 | 4 |
|  |  | Diarrhoea | 2 |  |
| 300 mg  (n=10) | None | ALP increase, WBC decreased, back pain | 1 | 15 |
|  |  | Glu increase | 2 |  |
|  |  | ALT increase, AST increase | 3 |  |
|  |  | T-Bil increase | 4 |  |
|  | Related | − | − | 0 |
| 600 mg  (n=10) | None | Plt decreased, AST increase, ALT increase, blood pressure increase | 1 | 4 |
|  | Undeniable | Headache | 1 | 1 |
| 900mg  (n=8) | Related | ALP increase, ALT increase, Suffocation feeling, T-Bil increase, WBC decreased | 1 | 7 |
|  |  | AST increase | 2 |  |
|  | Relatede | Diarrhoea | 1 | 4 |
|  |  | Headache | 3 |  |

Note: For the placebo, the adverse events encountered in the placebo group at each level were totaled. In the 300 mg group, the adverse events encountered in Period I and Period II were totaled.

2) Pharmacokinetics

Pharmacokinetic parameters of unchanged NIK-333 and NIK-333 lipids form at each level in the single administration study [Study of the effect of food (fed condition) at Level 1] are shown in the table below. Moreover, the changes of the concentrations of unchanged NIK-333 and NIK-333 lipids form [before administration and 0.5, 1, 2, 4, 6, 8 and 24 hr after administration] are shown in the figures below. A subject under Level 1 treatment who used Urso, a prohibited concomitant drug on the day of study drug administration and a subject under Level 3 treatment who used an NSAID (administration of loxonin for the treatment of headache), a prohibited concomitant drug, were excluded from data analysis.

As a result, dose-responses of unchanged NIK-333 were observed in AUC_0-24hr_ and Cmax.

Moreover, as seen with the results from the study of the effect of food at Level 1, the concentrations of unchanged NIK-333 in urine determined at the same time were all below the limit of quantitation both at Level 2 and Level 3, suggesting the absence of urinary excretion of unchanged NIK-333.

Pharmacokinetic parameters of unchanged NIK-333 and NIK-333 lipids form after single administration

|  |  | AUC_0-24hr_^#^  (ng･hr/mL) | Cmax^#^  (ng/mL) | tmax^#^  (hr) | t1/2^#^  (hr) |
| --- | --- | --- | --- | --- | --- |
| 300 mg^*^  (n=9) | Unchanged | 841.3± 648.3 | 316.1± 275.9 | 4.2±1.2 | 1.3±0.8 |
|  | Lipid body | 6497.7± 1819.0 | 694.1± 326.1 | 5.8±1.2 | 7.2±1.8 |
| 600 mg  (n=10) | Unchanged | 1873.8± 1063.0 | 468.4± 191.0 | 4.6±1.3 | 3.4±1.9 |
|  | Lipid body | 15883.3±17241.8 | 1450.5±1466.5 | 6.4±0.8 | 8.3±1.9 |
| 900 mg  (n=7) | Unchanged | 4009.7± 1090.9 | 906.9± 579.6 | 5.3±2.5 | 3.6±2.5 |
|  | Lipid body | 16274.0± 6478.0 | 1239.9± 331.6 | 9.1±6.6 | 8.9±2.0 |
| *: Data after food intake #:Mean±S.D. | | | | | |


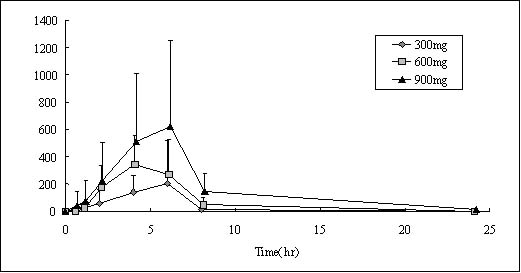


Unchanged NIK-333 concentration (ng/mL)

**Time course of unchanged NIK-333 concentration after single administration**

NIK-333 lipids form concentration (ng/mL)

**Time course of NIK-333 lipids form concentration after single administration**

[3] Repeated administration study and follow-up examination of the bone

1) Safety

A list of the adverse events encountered in the repeated administration study is shown in the table below. The numbers of adverse events observed were as follows: 62 events in 10 subjects at Level 1, 76 events in 11 subjects at Level 2, and 49 events in 8 subjects at Level 3. No clinically problematic adverse events were observed at Level 1 and Level 2 during the 48-week repeated administration period. At Level 1, however, gastric polyps, for which a causal relationship could not be ruled out, were seen in 2 subjects.

List of adverse events in repeated administration (1)

| Dose | Causal relationship | Events | Number of events | Total |
| --- | --- | --- | --- | --- |
| 300 mg  (At the start n=10)  (At the end n= 8) | None | Gastritis, Hypersensitivity, Hypertension, Rhinorrhoea, Nausea, Eczema, Toothache, Hordeolum, Blood cholesterol decreased, Cough, Headache, Productive cough, Sputum retention, Prostatitis, Varices oesophageal, Blood alkaline phosphatase increased, Weight decreased, Dermatitis, Hypergammagloblulinaemia benign monoclonal, Nasopharyngitis, Gamma-glutamyltransferase increased | 1 | 33 |
|  |  | Diarrhoea, Conjunctivitis, Oropharyngeal pain, Blood pressure increased | 2 |  |
|  |  | Pyrexia | 4 |  |
|  | Related | Thirst, Keratoconjunctivitis sicca, Pruritus generalised, Paraesthesia oral, Diarrhoea, Gastritis erosive, Gastric ulcer, Duodenal ulcer, Abdominal pain upper, Abdominal distension, Blood pressure increased, Nausea, Dyspepsia, Oropharyngeal discomfort, Weight decreased, Malaise, Night sweats, Bone density decreased, Aspartate aminotransferase increased, Alanine | 1 | 29 |
|  |  | LDH increase, Gastric polyps | 2 |  |
|  |  | Headache | 5 |  |
| 600 mg  (At the start n=12)  (At the end n= 7) | None | Diarrhoea, Constipation, Oedema peripheral, Pain in extremity, Occult blood, Large intestine carcinoma, Chest pain, Rhinitis, Dizziness, Diabetes mellitus, Dysphonia, Musculoskeletal stiffness, Dental care, Dry skin, Scapula fracture, Malaise, Blood urine present, Pruritus, Oropharyngeal Pain, Back pain, Protein urine present, Gamma-glutamyltransferase increased, Blood albumin decreased | 1 | 54 |
|  |  | Arthralgia, Varices oesophageal, Headache, Pyrexia | 2 |  |
|  |  | Eczema, ALT increase | 3 |  |
|  |  | AST increase | 4 |  |
|  |  | Cough | 5 |  |
|  |  | Nasopharyngitis | 8 |  |
|  | Related | Abdominal distension, Blood pressure increased, Urticaria, Abnormal sensation in eye, Hyperglycaemia, Duodenitis, Gastritis, Lip dry, Keratoconjunctivitis sicca, Oropharyngeal Pain, Malaise | 1 | 22 |
|  |  | Blood urine present,, Reflux oesophagitis, Hypertriglyceridaemia,  Gamma-glutamyltransferase increased | 2 |  |
|  |  | Headache | 3 |  |

List of adverse events in repeated administration (2)

| Dose | Causal relationship | Events | Number of events | Total |
| --- | --- | --- | --- | --- |
| 900 mg  (At the start n= 9)  * Discontinued during repeated administration | None | Vertigo, Abdominal pain upper, Joint swelling, Chest pain, Alanine aminotransferase increased, Aspartate aminotransferase increased, Blood lactate dehydrogenase increased, Varices oesophageal, Pyrexia, VIth nerve paralysis, Scar,Back pain, Protein urine, Oropharyngeal discomfort, Insomnia, Headache, Nausea | 1 | 23 |
|  |  | Nasopharyngitis | 6 |  |
|  | Related | Diarrhoea, Chapped lips, Abdominal pain upper, Retinal haemorrhage, Stomach discomfort | 1 | 26 |
|  |  | Dysgeusia, Abdominal pain, Protein urine present, Musculoskeletal stiffness | 2 |  |
|  |  | Urinary occult blood positive | 3 |  |
|  |  | Blood pressure increase | 4 |  |
|  |  | Headache | 6 |  |

Among the 9 subjects who moved on to repeated administration at Level 3, 1 subject was suspended from drug administration and 3 subjects were discontinued from the study due to increased blood pressure. In 1 subject who exhibited blood pressure increase, fundal haemorrhage was observed. However, the subject was placed on observation without requiring medical intervention.

The incidences of events suggesting the presence of effects on renal functions, such as protein urine and urinary occult blood, also increased as compared with Level 1 and Level 2. The numbers of subjects whose protein urine and urinary occult blood shifted to + or more after the start of repeated administration at each level are shown in the table below. Abnormal changes of protein urine and urinary occult blood after the start of repeated administration were also observed in subjects who showed no blood pressure increase, suggesting that NIK-333 affects renal functions.

Frequency of subjects exhibiting a shift to (+) after repeated administration at
each level

|  | Change  Dose | Level of change: From baseline | | | | |
| --- | --- | --- | --- | --- | --- | --- |
|  |  | None | 1 grade | 2 grades | 3 grades | 4 grades |
| Protein urine qualitative | Level 1 (300 mg) | 5/10 (50.0%) | 5/10 (50.0%) | − | − | − |
|  | Level 2 (600 mg) | 5/12 (41.7%) | 5/12 (41.7%)  1^*^ | 2/12 (16.7%) | − | − |
|  | Level 3 (900 mg) | 3/9 (33.3%)  1^*^ | 2/9 (22.2%)  1^*^ | 3/9 (33.3%)  1^*^ | − | 1/9 (11.1%)  1^*^ |
| Occult blood reaction | Level 1 (300 mg) | 4/10 (40.0%) | 4/10 (40.0%) | 2/10 (20.0%) | − | − |
|  | Level 2 (600 mg) | 8/12 (66.7%)  1^*^ | 2/12 (16.7%) | 1/12 (8.3%) | − | 1/12 (8.3%) |
|  | Level 3 (900 mg) | 1/9 (11.1%)  1^*^ | − | 6/9 (66.7%)  2^*^ | 2/9 (22.2%)  1^*^ | − |

*: Number of subjects who exhibited increased blood pressure

All of the adverse events observed at the relevant doses occurred within 24 weeks after the start of repeated administration. Since 1 subject was suspended from drug administration and 3 subjects discontinued the study due to the same event, continuous administration and enrollment of new subjects were discontinued out of concern for the subjects’ safety.

The clinical courses of the subject who was suspended from the study and the 3 subjects who discontinued the study are shown in the table below. While the condition after blood pressure increase varied among individuals, their blood pressures decreased after antihypertensive therapy. Two subjects under Level 3 treatment, at which the study was discontinued, were placed on concomitant antihypertensive. These subjects, however, showed no blood pressure increase, suggesting that blood pressure can be controlled by an antihypertensive therapy.

Clinical courses of the subjects at Level 3 who were suspended or discontinued from the study due to blood pressure increase

| Case of suspension | | At the start of repeated administration | At the time of suspension | Week 12 after discontinuation | Week 24 after discontinuation |
| --- | --- | --- | --- | --- | --- |
| 302 | Suspended from Week 24  (2003/12/11) | BP:108/69  HR:82 | BP:176/98  HR:77 | BP:155/88  HR:83 | BP:167/100  HR:76 |
|  |  | − | Not treated | Not treated | Referred to another hospital |
| Cases of discontinuation | | At the start of repeated administration | Upon discontinuation | Week 12 after discontinuation | Week 24 after discontinuation |
| 307 | Discontinued on Week 12  (2003/10/24) | BP:140/80  HR:86 | BP:176/111  HR:80 | BP:132/79  HR:83 | BP:145/93  HR:66 |
|  |  | − | Amlodin:  2.5 mg | The subject had failed to take most doses of Amlodin since 2004 at the subject’s own discretion. | The use of antihypertensive drugs was discontinued (2004/4/9). |
| 308 | Discontinued on Week 16  (2003/12/2) | BP:136/75  HR:85 | BP:186/110  HR:84 | BP:139/90  HR:83 | BP:148/84  HR:77 |
|  |  | − | Blopress:  4 mg  (12/3-12/10) | No antihypertensive therapy | |
| 309 | Discontinued on Week 4  (2003/10/24) | BP:150/100  HR:65 | BP:164/97  HR:82 | BP:131/81  HR:62 | BP:144/86  HR:66 |
|  |  | − | Adalat CR: 40mg The subject had been taking Adalat prescribed by a nearby physician as needed for 6 days. | Adalat was continued. | |

A list of the adverse events encountered in the follow-up examination of the bone is shown in the table below. The numbers of adverse events observed were as follows: 17 events in 7 subjects at Level 1, 5 events in 4 subjects at Level 2, and 15 events in 5 subjects at Level 3. No clinically problematic adverse events were observed at Level 1 and Level 2 during the 24-week period after completion of administration. At Level 3, nail disorder was seen in 2 subjects about 12 weeks after completion of administration, but it was a known adverse event associated with intake of a large amount of retinoid. Both of these subjects remitted within 24 weeks after completion of administration, but careful monitoring of the clinical course may be necessary depending on the long-term administration and the doses.

List of adverse events in follow-up examination of the bone

| Dose | Causal relationship | Events | Number of events | Total |
| --- | --- | --- | --- | --- |
| 300 mg | None | Diarrhoea, Abdominal distension, Dizziness postural, Decreased appetite, Malaise, Arthralgia, Stomatitis, Benign prostatic hyperplasia, Injury, Insomnia, Cough | 1 | 16 |
|  |  | Nasopharyngitis | 2 |  |
|  |  | Pyrexia | 3 |  |
|  | Related | Gastritis haemorrhagic | 1 | 1 |
| 600 mg | None | Headache, Hyperglycaemia, Urobilin urine, Osteoarthritis | 1 | 4 |
|  | Related | Hyperglycaemia | 1 | 1 |
| 900 mg | None | WBC decreased, Herpes zoster, LDH increase, Back pain, Weight decrease, Rib fracture, Cyanosis, γ-GTP increase, CKMB increase | 1 | 13 |
|  |  | AST increase, ALT increase | 2 |  |
|  | Related | Nail disorder | 2 | 2 |

2) Effects on bone

Changes in bone density (determined by DXA method) from the baseline (before repeated administration) up to 24 weeks after the end of repeated administration of NIK-333 are shown in the chart below. No marked changes were seen in the values from the baseline up to 24 weeks after the end of repeated administration at each level. There were no clinically problematic changes in thoracolumbar vertebral radiological findings, bone metabolism parameters, and bone density (determined by DIP method).


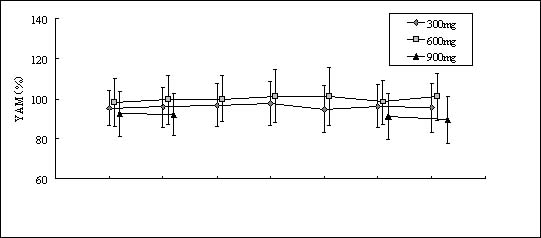


Before administration

Week 12

Week 24

Week 36

Week 48

Week 12 after completion

Week 24 after completion

Timing

Changes of bone density at each level

3) Pharmacokinetics

Changes in pharmacokinetics from the baseline up to 24 weeks after the end of administration are shown in the charts below. The time points of determination were the first day of administration, Week 24 of administration [before the start of administration and 0.5, 1, 2, 4 and 6 hours after administration], Weeks 2, 4, 8, 12, 16 and 20 of administration [before the start of administration and 2 hours after administration], Week 48 of administration, and Weeks 12 and 24 after the end of administration. At each level, a near-steady state was reached in Week 2 of repeated administration. Both unchanged NIK-333 and NIK-333 lipids form exhibited dose-response relationship after the start of administration.

Unchanged NIK-333 concentration (ng/mL)

0

Week 2

Week 4

Week 8

Week 12

Week 16

Week 20

Week 24

Week 48

Week 12

after

administration

Week 24

after

administration

Time course of unchanged NIK-333 concentration during repeated administration and follow-up examination of bone

NIK-333 lipid body concentration (ng/mL)

0

Week 2

Week 4

Week 8

Week 12

Week 16

Week 20

Week 24

Week 48

Week 12

after

administration

Week 24

after

administration

Time course of NIK-333 lipids form concentration during repeated administration and follow-up examination of bone

A list of subjects in whom NIK-333 was detected in the examination of the elimination phase at Week 48 of administration and at Week 12 and Week 24 after the end of administration is shown in the table below. NIK-333 lipids form (315 ng/mL) was detected at Week 12 after the end of administration in 1 subject at Level 2 (600 mg group), and the concentration of unchanged NIK-333 at that time was below the limit of quantitation. Moreover, at each level, unchanged NIK-333 was detected at concentrations close to the limit of quantitation after the end of administration in some subjects, but the extent was less than 1% of the Cmax after single administration at each level.

It is impossible to conclude definitively that NIK-333 is not accumulated in the body given the detectable levels of unchanged NIK-333 and NIK-333 lipids form in blood. Any potential accumulations, though, would be extremely small after the end of administration. However, NIK-333 lipids form was detected, at the concentration of 315 ng/mL, in only 1 out of 10 subjects treated with the same dose, and the concentration was only 45% of the mean Cmax (694.1 ng/mL) observed at Level 1 (300 mg group) in the single administration study, which is equivalent to the single dose given at Level 2 (600 mg group). Moreover, the concentration determined after the end of administration was below the limit of detection. Lipid body was not detected in the other 27 subjects for whom the elimination phase at each level could be determined.

These results suggest that, although NIK-333 is accumulated in the body, the extent and frequency are small and are not clinically problematic.

List of subjects with detectable level of NIK-333 in the elimination phase

| Subject No. |  | Week 12 after the end of administration | Week 24 after the end of administration |
| --- | --- | --- | --- |
| 107 | Unchanged  NIK-333 (ng/mL) | 2 | 0 |
|  | NIK-333 lipids form (ng/mL) | 0 | 0 |
| 207 | Unchanged NIK-333 (ng/mL) | 0 | 0 |
|  | NIK-333 lipids form (ng/mL) | 315 | 0 |
| 211 | Unchanged NIK-333 (ng/mL) | 2 | 2 |
|  | NIK-333 lipids form (ng/mL) | 0 | 0 |
| 212 | Unchanged NIK-333 (ng/mL) | 2 | 0 |
|  | NIK-333 lipids form (ng/mL) | 0 | 0 |
| 304 | Unchanged NIK-333 (ng/mL) | 2 | 0 |
|  | NIK-333 lipids form (ng/mL) | 0 | 0 |
| 306 | Unchanged NIK-333 (ng/mL) | 6 | 1 |
|  | NIK-333 lipids form (ng/mL) | 0 | 0 |

[4] Discussion of phase I studies

1) Safety

Adverse events of particular clinical significance were not observed at Level 1 (300 mg group) and Level 2 (600 mg group), and tolerability was judged to have been confirmed. However, the gastric polyp observed at Level 1 was considered an event requiring endoscopic exam in the subsequent phases.

At Level 3 (900 mg group), blood pressure increase was observed in 4 out of 9 subjects during the period of 24 weeks from the start of repeated administration. Since this event was an unknown adverse reaction, and the mechanism of its onset has not been understood, administration of the study drug and enrollment of new subjects were discontinued. Although the symptom related to the event disappeared after pharmacotherapy, the extent shifted from the normal range to the range of severe hypertension in some subjects, prompting the event to be regarded as an important event that could have accompanying secondary inconveniences unless some treatment was given. Moreover, some subjects exhibited protein urine and urinary occult blood in association with blood pressure increase, and some subjects exhibited abnormal changes in protein urine and urinary occult blood without blood pressure increase. The frequency of the occurrence of abnormal changes in urinary qualitative parameters was high at Level 3. Furthermore, nail disorder, which is known to be induced by taking a large amount of retinoid, was seen in 2 subjects about 12 weeks after the end of administration. For these reasons, 900 mg was deemed inappropriate as a clinical dose.

The effect of NIK-333 on bone, which was seen in non-clinical studies, was not observed at any of the levels assessed.

2) Pharmacokinetics

There were no differences in the plasma concentrations of unchanged NIK-333 in the fed and fasted conditions.

The pharmacokinetic parameters (AUC_0-24hr_ and Cmax) of unchanged NIK-333 in plasma were confirmed to exhibit linearity after single or repeated administration of 300 mg, 600 mg and 900 mg.

Since almost no unchanged NIK-333 and NIK-333 lipids form were seen in plasma at Week 48 of administration and Week 12 and Week 24 after the end of administration, the drug was not considered to be accumulated in the body during repeated administration.

Excretion of unchanged NIK-333 in urine was not seen after single administration.

3) Conclusions

It was considered appropriate to set the upper limit of the clinical daily dose of NIK-333 at 600 mg on the basis of the safety and pharmacokinetics results of the phase I clinical studies. However, given the duration from the start of repeated administration to the occurrence of the increased blood pressure in the group treated with 900 mg, the possibility that that the same event may occur during administration of 600 mg for more than 48 weeks, the length of time in which assessments could be carried out in this study, canot be ruled out. With both abnormal changes in protein urine and urinary occult blood and delayed adverse events (nail disorder) after study drug administration also taken into consideration, it was deemed necessary to carefully cope with the occurrence of cardiovascular, urologic and endoscopic adverse reactions during the administration period and to pay due attention to the follow-up of delayed adverse events after the end of administration, when conducting a long term administration study at 300 mg and 600 mg per day.

(3) Effects on hepatocarcinogenesis (Clinical study)

[1] Preventive effects of polyprenoic acid on recurrent hepatpma and second primary hepatoma after treatment^13)^

Subjects: Patients who have completed the treatment for hepatoma by subtotal resection of the liver and PEI

Study centers: Gifu University School of Medicine, the Institute of Gastroenterology of Tokyo Women’s Medical University, Gifu Municipal Hospital, Gihoku Hospital, Gifu Red Cross Hospital, and Murakami Memorial Hospital Asahi University; 6 centres in total

Study period: 1990 through February 1996 (including the follow-up period)

Number of subjects: 89 (active drug: 44, placebo: 45) *By random assignment

Dosage regimen: Oral administration of 300 mg each time, twice daily (morning and evening)

Administration period: 12 months

Inclusion criteria: - Patients who have been confirmed to have no more hepatpma by ultrasonography and plain or contrast CT of the entire remnant liver and blood tests including α-fetoprotein measurement

- Patients under the age of 75 in a favorable physical condition

Exclusion criteria: - Patients with a serum total bilirubin level of 5.0 mg/dL (84 µmol/L) or above

- Patients who have a severe heart, kidney or blood disorder which may be exacerbated by the study drug

*Consenting patients also agreed to refrain from the use of vitamin A supplements during the study period.

Study method: Drug administration was started no later than 8 weeks after surgery or PEI. Ultrasonography was performed every 3 months and CT was performed every 6 months during the treatment and follow-up periods. On each medical examination, history was taken and physical examination was conducted to record the patient’s systemic state, and venous blood samples were collected for laboratory tests including determination of plasma drug concentrations and α-fetoprotein levels.

Endpoint: Recurrence of hepatoma

*The size and position of the lesion were recorded on each ultrasonographic and CT examination. All of these lesions were biopsied within 1 week, and were examined histopathologically.

Results: No statistically significant differences were observed between the groups in terms of factors that presumably may have an effect on treatment failures (age, gender, causes and severity of liver disorders, number and size of primary hepatomas, and type of primary treatment).

Patients who took at least one capsule of the study drug during the study period were subjected to the analysis of adverse reactions. One patient who took the active drug complained of severe headache on the first day of treatment and was discontinued administration of the study drug. In the placebo group, one patient exibited a severe rash and another had moderate nausea, and admistration of the study drug was discontinued in both patients. Throughout the study period, adverse reactions characteristic to retinoids – xeroderma, cheilitis, and conjunctivitis – were not observed in either of the groups. No laboratory abnormalities possibly related to the treatment was reported in either group. The mean plasma drug concentration in 39 patients receiving the active drug for 1 year was 44.9 ± 13.9 ng/mL after 1 year of administration.

The incidence of hepatoma during the observation period of mean 3 years and 2 months on the average (including 1-year administration period) in the active drug group (12/44: 27%) was significantly lower than that in placebo group (22/45: 49%) (p = 0.04). The incidence of second primary hepatoma also showed a significant difference between the active drug group (7/44: 16%) and the placebo group (20/45: 44%) (p = 0.004).

[2] Results after follow-up for 62 months on average^14)^

In the above study on the preventive effect of polyprenoic acid on the reccurence of hepatoma and secondary primary hepatoma after hepatoma treatment18), a follow-up observation of mean 62 months was performed after the end of administration. The results showed a significant difference in the survival rate between the active drug group (74%) and the placebo group (46%) (p＝0.04). The relative risk of death was 0.3 (95% CI: 0.1 to 0.8).

## 2.4 Summary of known and potential risks and benefits for subjects

(1) Potential risks of the study drug NIK-333

In the phase I clinical studies, blood pressure increase was observed as an adverse reaction in 4 of the 9 subjects treated with 900 mg. Moreover, the incidences of protein urine and urinary occult blood, which suggest the effect on the renal functions, were higher at this dose than at other doses, and nail disorder occurred during the follow-up period after the end of drug administration.

In a clinical study conducted by Muto et al. to examine prevention of recurrent HCC by E-5166, which is the same substance as NIK-333 (600 mg/day, 1 year), headache (1 out of 44 subjects) was observed.^13)^ In a clinical study of E-5166 in patients with skin diseases (300 mg/day, 8 weeks), 180 subjects were included in the safety analysis, with thirst, cheilitis, lip dry, itching and other adverse reactions seen in 13% of the subjects (24 out of 180 subjects), and serum triglyceride increase seen in 1.1% of the subjects (2 out of 180 subjects). However, all of these events were reported to be transient.^12)^ (See the table below.)

The results of non-clinical studies indicate that the study drug may affect liver functions and lipid metabolism. It also has a potential to induce gastrointestinal symptoms (diarrhoea, soft stools, etc.) and skin and mucosal disorders. Since fractures were seen at the dose of 200 mg/kg/day in a 4-week oral dose toxicity study in rats and at 100 mg/kg/day in a 26-week oral dose toxicity study in rats, the study drug is likely to affect bone metabolism.

In a carcinogenicity study in mice, the incidence of angiosarcoma increased at the dose of 40 mg/kg/day or more in both male and female mice, and the increase was confirmed to be significant in male mice treated with 80 mg/kg/day. Since induction of vascular tumors has been seen with other types of retinoid (etretinate^17)^ and fenretinide^18)^), an association cannot be ruled out between the vascular tumor induction and the action of retinoid. However, induction of vascular tumors by retinoid, including NIK-333, is a phenomenon that has been observed only in mice but not in rats. Moreover, induction of vascular tumors by retinoid has not been reported in humans. Therefore, induction of vascular tumors by NIK-333 is presumed to be an action specific to mice, the species in which numerous chemical substances are known to induce vascular tumors.^21, 22)^

In a carcinogenicity study in rats, thikening (nonneoplastic lesion) of the anterior stomach increased at the dose of 10 mg/kg/day or more, and squamous cell carcinoma of the anterior stomach was seen in a small number of animals at the dose of 20 mg/kg/day or more. The proliferative action (thikening) and the inflammatory action (erosion and oedema) on the anterior stomach epithelia, which have also been seen in rat repeated dose toxicity studies, were presumed to be involved in the mechanism of anterior stomach carcinogenesis by NIK-333. Potential relationship between the tumor occurrence in the anterior stomach (squamous epithelia), which was seen only in rodents, and the potential risk of induction of gastric cancer in humans has not been elucidated to date. Any changes attributable to NIK-333 administration have not been seen in the rat glandular stomach, which has the same histologic structure (columnar epithelia) as that of humans. Moreover, injurious effects on the stomach and the onset of proliferative lesions potentially leading to tumor induction were not observed in long-term (52 weeks) administration of NIK-333 to dogs, which have a stomach anatomically resembling that of humans. Therefore, NIK-333 is considered unlikely to induce tumors in the stomach of mammals other than rodents.

In a carcinogenicity study in mice, the occurrence of squamous cell carcinoma was not observed in the anterior stomach, but hyperkeratosis attributable to NIK-333 administration was seen not only in the anterior stomach but also in the esophagus. In contrast, effects of NIK-333 on the esophagus have not been observed in rats and dogs. In mice, it is conceivable that the esophageal epithelium was exposed to NIK-333 for some reasons during forced oral administration via a gastric tube and became affected by the study drug. When extrapolating the data to humans, it should be noted that the drug is administered in capsules, and the chance of exposure of NIK-333 to the esophagus is small. Therefore, the drug is considered to have almost no effects.

Type, severity and number of episodes of adverse drug reactions in a clinical study in patients with skin diseases ^12)^

Incidence of adverse drug reactions = (Number of subjects exhibiting adverse reactions)/(number of subjects studied) = 24/180 subjects = 13.3%

| Symptom | Severe | Moderate | Mild | Total number of events | Frequency (%) |
| --- | --- | --- | --- | --- | --- |
| Thirst | 0 | 0 | 11 | 11 | 6.1 |
| Cheilitis | 0 | 2 | 6 | 8 | 4.4 |
| Dry lip | 0 | 0 | 4 | 4 | 2.2 |
| Itching | 0 | 0 | 3 | 3 | 1.6 |
| Folliculitis | 0 | 1 | 0 | 1 | 0.5 |
| Plantar fissure | 0 | 0 | 1 | 1 | 0.5 |
| Nail fragility | 0 | 0 | 1 | 1 | 0.5 |
| Flickering feeling in the eyes | 0 | 0 | 1 | 1 | 0.5 |
| Itchy sensation of eyelids | 0 | 0 | 1 | 1 | 0.5 |
| Foreign body feeling of oesophagus | 0 | 0 | 1 | 1 | 0.5 |
| Stomach discomfort | 0 | 0 | 1 | 1 | 0.5 |
| Diarrhoea | 0 | 0 | 1 | 1 | 0.5 |
| Queasy | 0 | 0 | 1 | 1 | 0.5 |
| Burning sensation in face | 0 | 1 | 0 | 1 | 0.5 |
| Malaise in whole body | 0 | 1 | 0 | 1 | 0.5 |

Abnormal laboratory test values in a clinical study in patients with skin diseases ^12)^

| Parameter | Total number of events | Frequency (%) |
| --- | --- | --- |
| Transient increase of TG | 2 | 1.1 |

(2) Potential risks in this study

In this study, liver biopsy will be performed for determination of the liver gene expression profile and drug concentrations. Risks of complications associated with liver biopsy such as haemorrhage (haemorrhage from the liver surface at the puncture site), infection, pneumothorax (it can be induced if the lung is injured during the puncturing process), and shock (it can be caused by incompatibility with anesthesia) cannot be completely ruled out, although the likelihood is extremely small. Therefore, the operation should be performed with measures of precaution, including careful preliminary examination.

(3) Benefits for subjects

In non-clinical studies, oral administration of the doses of 40 mg/kg or more significantly inhibited hepatocarcinogenesis in rats induced by 3'-MeDAB and DEN^15)^.

Muto et al. reported that in their clinical study of E-5166, a substance identical to NIK-333, a daily dose of 600 mg administered for 1 year to patients who received treatment for primary HCC significantly inhibited recurrent HCC, as compared with the placebo group,^13)^ and improved the survival rate.^14)^

## 2.5 Compliance with various standards

This study will be conducted in compliance with this study protocol, the standards stipulated in Article 14, Clause 3 and Article 80-2 of the Pharmaceutical Affairs Law, the “Contents of the Good Clinical Practice (GCP) (Report of the Central Pharmaceutical Affairs Council)” (hereinafter referred to as Report GCP) dated March 13, 1997, Ordinance No. 28 of the Ministry of Health and Welfare dated March 27, 1997 “Ordinance on Good Clinical Practice” (Ordinance GCP), and *Yakuhatsu* No. 430 dated March 27, 1997 “Enforcement of the Ordinance on Good Clinical Practice,” Ordinance No. 172 of the Ministry of Health, Labour and Welfare dated December 21, 2004 “Ordinance for Partially Revising the Ordinance on Good Clinical Practice,” the Declaration of Helsinki, and the study contract signed by the sponsor and the study center.

# 3. Study objectives

This 8-week repeated administration study compares the NIK-333 doses of 300 mg/day and 600 mg/day in patients who were completely cured of hepatitis C virus (HCV)-positive HCC by examining the changes in liver and peripheral gene expression profiles before and after administration of each dose and the drug concentrations in the liver and plasma. After the above test (liver biopsy), NIK-333 of 600 mg/day is administered to all subjects until Week 96 (88 weeks) to investigate safety, with a focus on the cardiovascular system, and the changes of plasma drug concentrations during long-term administration.

# 4. Study design

## 4.1 Study design

(1) Type of study

Step I: Open-label, randomized, parallel-group, comparison study in which NIK-333 is administered repeatedly at the dose of 300 mg/day or 600 mg/day (Week 0 to Week 8)

Step II: Open-label study in which NIK-333 is administered at 600 mg/day alone (Week 8 to Week 96)

(2) Flow chart of the study design, procedure and steps

Follow-up examination

Subject selection period

Study drug administration period

Step I

Step II

| Radical therapy  0  Gene expression profile  Pharmacokinetics in liver and peripheral blood  Safety evaluation  Pharmacokinetics in plasma  8  Week 12 after  completion of  administration  Week 96 |  | Briefing/Informed consent |  | Confirmation of complete cure |  | Registration |  |  | |
| --- | --- | --- | --- | --- | --- | --- | --- | --- | --- |
|  |  |  |  |  |  |  |  | Administration of 300 mg/day |  |
|  |  |  |  |  |  |  |  |  |  |
|  |  |  |  |  |  |  |  | Administration of 600 mg/day | Administration of 600 mg/day |
|  |  |  |  |  |  |  |  |  |  |

(3) Target sample size

12 subjects

Subjects will be divided into the following two groups in Step I (Week 0 to Week 8).

NIK-333 300-mg group: 6 subjects

NIK-333 600-mg group: 6 subjects

All subjects will receive 600 mg/day in Step II (Week 8 to Week 96).

## 4.2 Evaluation parameters

[1] Gene expression profile

Liver and peripheral blood gene expression profiles determined by the microarray method

[2] Safety

Adverse events, physiological tests, laboratory tests (hematology tests, blood biochemistry tests, urinalysis, blood pressure parameters), abdominal imaging diagnosis, endoscopy (esophagus/stomach), bone mineral analysis (DXA method), ECG, echocardiography, pulse wave examination (PWV/ABI), fundus examination

[3] Pharmacokinetics (unchanged NIK-333 and NIK-333 lipids form)

1) Drug concentrations in the liver: Concentrations of unchanged NIK-333 and NIK-333 lipids form

2) Drug concentrations in plasma: Concentrations of unchanged NIK-333, NIK-333 lipids form and NIK-333 metabolite

3) Drug concentration in urine: Unchanged NIK-333

[4] Exploratory biomarker

TGF-α concentration in plasma (biomarker related to gene expression)

## 4.3 Blinding procedures and maintenance of blindness

No blinding procedures are conducted in this study.

## 4.4 Administration method, doses and administration period

(1) Administration method

Orally administer the designated dose twice daily, after breakfast and after supper.

[Rationale]

Administration after meals was selected because: the results of the phase I clinical studies showed no significant differences in plasma concentration of unchanged NIK-333 between the fed and fasted conditions; good compliance is expected for administration after meals in clinical settings; and there are no reasons why administration in the fasted condition should be strongly recommended.

(2) Doses

NIK-333 of 300 mg (2 capsules x twice/day) or 600 mg (4 capsules x twice/day) (One capsule contains 75 mg of NIK-333.)

[Rationale for the doses]

Muto et al. administered to patients who had received treatment for HCC a substance identical to NIK-333 at the dose of 600 mg/day (twice daily) for 1 year, and found that the substance had an inhibiting effect on recurrence of HCC.^13)^ On the basis of these findings, a single administration study of 3 doses (600 mg/day as the main dose and 300 and 900 mg/day) and a 48-week repeated administration study were conducted as phase I clinical studies to confirm the pharmacokinetics and tolerability of NIK-333. As a result, unchanged NIK-333 was confirmed to exhibit linear pharmacokinetics in the dose ranging from 300 to 900 mg/day. With regard to tolerability in the repeated administration study, blood pressure increase was observed as an adverse drug reaction at the dose of 900 mg/day (twice daily), but no serious and significant adverse drug reactions were seen at 300 and 600 mg/day (twice daily).

In the pharmacology studies, on the other hand, NIK-333 administered at the dose of 40 mg/kg was confirmed to significantly inhibit chemical carcinogenesis induced by 3'-MeDAB and DEN^15)^ in rat liver. The peak plasma concentration of unchanged NIK-333 after single administration of 40 mg/kg (effective dose) in rats was 0.5 µM (151 ng/mL). Since the liver/plasma concentration ratio after single oral administration in rats is 2 to 6, the concentration of unchanged NIK-333 in liver tissues is estimated to be 1 to 3 µM. Therefore, after a dose of 40 mg/kg, the NIK-333 concentration in liver tissues was assumed and confirmed by *in vitro* studies to have reached the IC_50_ for the cell growth-inhibiting action (0.220 µM, 66.5 ng/mL), the EC_50_ for the differentiation-inducing action (4.3 µM, 1300 ng/mL), and the IC_50_ for the apoptosis-inducing action (1.91 µM, 578 ng/mL).

In the phase I clinical study, the peak concentrations of unchanged NIK-333 after single administration of 300, 600 and 900 mg were 1.0 µM (316.1 ± 275.9 ng/mL), 1.5 µM (468.4 ± 191.0 ng/mL) and 3.0 µM (906.9 ± 579.6 ng/mL), respectively. From these results, the concentrations in human liver tissues at each dose (twice daily) are calculated to be 1.0 to 3.1 µM (316.1 to 948.3 ng/mL), 1.5 to 4.6 µM (468.0 to 1404.0 ng/mL) and 3.0 to 9.0 µM (907.0 to 2721.0 ng/mL), respectively, suggesting that the concentrations which are confirmed to be effective in *in vitro* studies have already been reached after administration of 300 mg/day. In other words, judging from the concentrations of unchanged NIK-333 in plasma and liver tissues obtained from the effective dose in the pharmacology studies, the dose of 300 mg/day (twice daily) is expected to be effective based on comparison between rats and humans. The dose of 600 mg/day (twice daily) is equivalent to a twice-daily administration of 100 mg/kg in rats in terms of the blood concentration, and may be regarded as an excessive dose from the point of view of pharmacokinetics.

For these reasons, we have decided to determine the gene expression profile of liver, and peripheral blood and drug concentrations in the liver and plasma before and after 8-week repeated administration of the following two doses: 300 mg/day (twice daily) of which efficacy can be expected from the blood concentration, and 600 mg/day (twice daily) which has been confirmed to be effective by Muto et al., and which has also been confirmed to be tolerable during 48-week administration in phase I clinical studies (Step I). If the liver biopsy scheduled on the day of evaluation at Week 8 is postponed in Step I, the period of Step I will be extended until completion of liver biopsy. In other words, the period of administration of two doses, i.e., 300 mg/day (twice daily) and 600 mg/day (twice daily) will also be extended. After completion of the above test (liver biopsy), all subjects will receive 600 mg/day (twice daily) of NIK-333 until Week 96, and the safety and the time course of drug concentration in plasma during long-term repeated administration at a high dose will be examined (Step II).

(3) Study drug administration period

96 weeks (Step I; Week 0 to Week 8, Step II; Week 8 to Week 96)

[Rationale]

Step I:

The administration period for determination of the gene expression profile was selected with an intention to determine the best possible timing for identifying expressions of the genes that quickly respond to the drug after the start of NIK-333 administration, while taking into consideration the physical burden of liver biopsy on the patients and feasibility of administration at the study center. Since the results of examination of pharmacokinetics in the phase I repeated administration study of NIK-333 showed that a steady state was reached at Week 2 of repeated administration, the study drug administration for at least two weeks was considered necessary for determination of the gene expression profile. Also considering the stress imposed on the liver by the two liver biopsies, the administration period for determination of the gene expression profile was set at 8 weeks.

Step II:

The experience of long-term administration of the study drug is confined to the clinical study conducted by Muto et al.^13)^ and the phase I study. The reports by Muto et al. suggest that the effect of 1-year administration tends to be maintained for a long period. However, due to the small sample size, it has not confirmed whether adequate clinical effects are maintained long during 1-year administration. Therefore, the administration period has been set at 96 weeks in the phase II/III study of NIK-333 to examine the changes in the recurrence-inhibiting effect of repeated administration of the study drug. Thus, the optimal administration period will be evaluated based on the comparison between the inhibitory effect after completion of administration and the preceding inhibitory effect. However, while the safety of NIK-333 in administration for up to one year has been confirmed in the phase I study, safety in long-term administration for more than one year has not been investigated to date. In particular, in view of the blood pressure-increasing effect observed at 900 mg/day, the safety and pharmacokinetics of NIK-333 of 600 mg/day in administration for up to 96 weeks will be investigated by examining the cardiovascular and urinary systems.

## 4.5 Follow-up examination

(1) Follow-up examination of safety

Conduct medical examination, determination of vital signs, abdominal imaging diagnosis, determination of tumor markers, and laboratory tests at Week 12 after completion of the study drug administration.

[Rationale]

Conduct tests to ensure the subjects’ safety after completion of the study drug administration.

(2) Follow-up examination of pharmacokinetics

Determine drug concentrations in plasma at Week 12 after completion of the study drug administration.

[Rationale]

To confirm elimination of the study drug from plasma after repeated administration of NIK-333

(3) Other follow-up examinations

If any other abnormal findings (adverse events or abnormal laboratory values) are observed, conduct follow-up examinations as needed.

## 4.6 Expected period of subjects’ participation

The expected period of subjects’ participation is equivalent to the study drug administration period (96 weeks).

## 4.7 Amount of liver tissue samples collected and frequency of sample collection

Perform liver biopsy and collect liver tissues on the day before the start of study drug administration and at Week 8 of study drug administration. Collect about 40 mg of liver tissue sample in one procedure of liver biopsy and a total of about 80 mg of tissue.

## 4.8 Amount of blood collected and the number of days of blood collection

The amount of blood collected from the subject on each visit is shown below.

As the laboratory test facility switches to a different method of HCV-RNA test (quantitative), the amount of blood collected will increase from 34 mL to 39 mL beginning in August 2008 for the days of evaluation on Week 48 and Week 96 of study drug administration.

|  | Amount of blood collected per day (ml) | Number of days of blood collection |
| --- | --- | --- |
| At the time of registration | 12 | 1 |
| Day of the start of administration | 39 | 1 |
| Day of evaluation at Week 8 of administration | 39 | 1 |
| Days of evaluation at Weeks 12, 36, 60 and 84 of administration | 18 | 4 |
| Days of evaluation at Weeks 24 and 72 of administration | 24 | 2 |
| Days of evaluation at Weeks 48 and 96 of administration | 39 | 2 |
| Days of evaluation at Weeks 4, 16, 20, 28, 32, 40, 44, 52, 56, 64, 68, 76, 80, 88 and 92 of administration | 14 | 15 |
| At the time of follow-up examination (Week 12 after completion of administration) | 22 | 1 |
| Total | 520 | 27 |

This table does not include any blood samples collected at the discretion of the investigator or the subinvestigator due to the occurrence of adverse events, etc.

## 4.9 Amount of urine collected and frequency of urine sample collection

Urine sample will be accumulated for 24 hours at the time of “4.7 Liver tissues collection (during hospitalization for liver biopsy),” on the day of evaluation at Week 48, on the day of evaluation at Week 96, and in the presence of increased blood pressure (if possible).

# 5. Criteria for inclusion and exclusion of subjects

Before selecting the subjects, the investigator, among others, will carefully evaluate patients for their fitness to participate in the study, with consideration given to the condition of health, age, gender, ability to give consent, depending relationship with the investigator, etc., and participation in other clinical studies so as to comply with human rights protection and the study objectives.

## 5.1 Study subject population

Subjects are patients who have undergone topical medical therapy or surgical resection after a diagnosis of HCV-positive HCC and have been confirmed to have achieved a complete cure from HCC.

[Rationale]

In Japan, 15.5% and 71.8% of the patients with HCC are believed to be positive for HBs antigen and HCV antibody, respectively.^3)^ Moreover, the cumulative incidence rate of HCV-positive HCC is much higher than that of HCC induced by HBV or alcohol,^23)^ and the risk of recurrence is also high. Furthermore, in some cases of HBV-positive HCC, HCC may develop in the early stage of hepatitis without progression from chronic hepatitis to liver cirrhosis. Therefore, unlike HCV-positive HCC, the process of carcinogenesis of HBV positive HCC is not uniform, and other factors may be involved. In light of these facts, the patients with a complete cure from HCV-positive HCC were considered appropriate as the subjects to be analyzed for evaluation of the study drug’s efficacy, which is “the effect to inhibit recurrent HCC,” considering the number of patients, risk of recurrence, and uniformity of the process of carcinogenesis in the phase II/III study of NIK-333. In this clinical pharmacology study, the same patients as those in the phase II/III study of NIK-333 were selected as the subjects for the analysis of the gene expression profile, with the aim of identifying the drug response gene for NIK-333.

## 5.2 Inclusion criteria

Select patients who meet the following criteria as the subjects.

[1] Patients with HCV-positive HCC who meet the following conditions before radical treatment

1) Patients diagnosed as having typical HCC on dynamic CT (MD) or CTA/CTAP

2) Patients with first primary HCC or the first recurrence of primary HCC (recurrence after an interval of at least one year after treatment of the first primary HCC)

[2] Patients who received one of the following treatments

1) Topical medical therapy

Patients who have undergone local radical therapy of HCC mainly by radiofrequency ablation (RFA). (However, percutaneous ethanol injection (PEI) or percutaneous microwave coagulation therapy (PMC) in combination with RFA are allowed.)

2) Surgical resection

Patients who have undergone liver resection

[3] Patients in whom complete cure has been confirmed by the following methods

1) Topical medical therapy

The dynamic CT images taken from 8 weeks (56 days) to 12 weeks (84 days) after topical medical therapy show no findings suggestive of recurrence at the treatment site and other sites, and the investigator or the subinvestigator has confirmed complete cure

2) Surgical resection

The dynamic CT images taken from 8 weeks (56 days) to 12 weeks (84 days) after liver resection show no findings suggestive of recurrence or residue, and the investigator or the subinvestigator has confirmed complete cure

[4] Patients who are able to begin treatment with the study drug within 8 weeks (56 days) after the dynamic CT to confirm complete cure

[5] Patients confirmed of satisfying the following conditions based on the screening performed at subject registration

1) HCV-RNA is present in serum.

2) Grade A or B on Child-Pugh classification ^24.25)^

| Score | | 1 | 2 | 3 |
| --- | --- | --- | --- | --- |
| liver encephalopathy | | None | Mild | Occasional coma |
| Ascites | | None | Small amount | Moderate amount |
| Serum bilirubin (mg/dL) | Patients other than those mentioned below | <2 | 2-3 | 3< |
|  | Patients with biliary cirrhosis primary | <4 | 4-10 | 10< |
| Serum albumin (g/dL) | | 3.5< | 2.8-3.5 | <2.8 |
| Prothrombin (s: seconds), (%: activity value) | | <4，70%< | 4-6，40-70% | 6<，<40% |

Grade A:5-6 points Grade B: 7-9 points Grade C: 10-15 points

3) Platelet count of 50,000/µL or higher

[6] Patients of the age of 20 years or older at the time of informed consent

[Rationale]

[1]: The patients with HCV-positive HCC of the first onset or first recurrence are deemed less likely to have intrahepatic metastasis than those who have repeated recurrences and were judged to be appropriate for confirming efficacy of the study drug (effect to inhibit recurrent HCC) in the phase II/III studies of NIK-333. In this clinical pharmacology study, the same patients will be selected as the subjects.

[3]: To improve the accuracy of confirmation of complete cure by evaluation of images and to exclude local recurrence (residue) as much as possible

[4]: It was considered necessary to start administration of the study drug within a certain period after confirmation of complete cure.

[5]: 2) and 3): To exclude patients with a severe condition in terms of the remaining liver functions.

[6]: To make it essential to obtain informed consent from the patient personally, or from the patient personally and his or her representative

## 5.3 Exclusion criteria

Patients who fall under any of the following criteria will be excluded.

[1] Patients positive for HBs antigen

[2] Patients showing portal infiltration of HCC on CT images

[3] Patients who have also undergone transcatheter arterial embolization therapy (TAE/TACE) as combination with the radical therapy

[4] Patients who have received other study drugs, anticancer drugs, interferons, or vitamin K_2_ after radical therapy

[5] Patients who have hypertension as a complication, and whose blood pressure cannot be controlled by drug therapy (systolic blood pressure of 160 mmHg or higher or diastolic blood pressure of 100 mmHg or higher, as determined at subject registration)

[6] Patients who have a history of allergy to CT contrast media, and whose participation in this study is judged to be inappropriate by the investigator or the subinvestigator

[7] Patients whose hemoglobin content is less than 8.0 g/dL and whose prothrombin time (%: activity value) is less than 40%, patients who are taking drugs with a potential of inducing blood coagulation abnormality, and patients who are judged to be ineligible for liver biopsy by the investigator or the subinvestigator

[8] Patients with a history of total gastrectomy

[9] Patients with serious complications (serious renal disorder, heart disease, diabetes mellitus, autoimmune disease, asthma, etc.)

[10] Patients confirmed of having another malignant neoplasm or who had undergone a radical therapy within the last 5 years to treat another malignant neoplasm (however, this does not apply to endoscopic resection and resection of intraepithelial carcinoma）

[11] Patients who are pregnant, who have a possibility of being pregnant or who have a desire to become pregnant during the study period

[12] Lactating women

[13] Patients who have a history of allergy to retinoid-related substances (vitamin A, etc.) in the past

[14] Other patients deemed ineligible to participate in the study by the investigator or the subinvestigator

[Rationale]

[1]: The subjects of the ongoing phase II/III study of NIK-333 are patients with HCV-positive HCC. Moreover, while the liver gene expression profiles reportedly are different between patients with HBV-positive hepatitis and patients with HCV-positive hepatitis, the virological background of the patients should be made uniform.

[2]: Because NIK-333 is not indicated for highly malignant HCC associated with portal infiltration

[3]: To avoid the use of anticancer drugs in transcatheter arterial embolization therapy (TAE/TACE)

[5]: To ensure safety of subjects in light of the fact that obvious increase of blood pressure was observed in the group treated with 900 mg in the phase I studies

[7]: To ensure the subjects’ safety during liver biopsy

[8]: Because absorption of NIK-333 may be affected

[11]: Because NIK-333 was confirmed to have teratogenicity and to induce miscarriage in the reproductive and developmental toxicity study

[12]: Because safety for fetuses has not been confirmed

[4]，[6]，[9]，[10]，[13]，[14]: To ensure the subjects’ safety and to eliminate any factors interfering with evaluation of the safety of the study drug

# 6. Study drug

## 6.1 Name of study drug, components, contents, dosage form, etc.

(1) Name of study drug

Test drug: NIK-333

(2) Chemical name

（2*E*,4*E*,6*E*,10*E*）-3,7,11,15-Tetramethylhexadeca-2,4,6,10,14-pentaenoic acid

(3) Structural formula

Molecular formula: C_20_H_30_O_2_

Molecular weight: 302.45

(4) Content and dosage form of the study drug

Test drug: NIK-333

Yellow-brown soft capsules containing 75 mg of the active component in one capsule

(5) Stability of the study drug

The study drug was stable for 36 months in the long-term storage study (25°C, 60%RH) and for 6 months in the accelerated study (40°C, 75%RH). The expiration date will be 3 years (36 months) from the date of production of the study drug.

## 6.2 Packaging and labeling of the study drug

(1) Packaging

The study drugs are provided in PTP package containing 10 capsules per sheet. Ten sheets of PTP package are put in an aluminum bag, and one aluminum bag is placed in a small box. Fifteen small boxes are put in a large box.

(2) Labeling

The following information will be indicated on the label.

[1] The fact that it is an investigational drug

[2] Name and address of the sponsor

[3] Identification code and batch number

[4] Number of capsules

[5] Storage method

[6] Expiration date

(3) Storage condition

The PTP package will not be opened and will be kept at room temperature until the time of administration of the study drug.

## 6.3 Management of study drugs

(1) Delivery of the study drug

The sponsor will deliver the study drug directly to the study drug storage manager of the study center after the study contract has been signed by the sponsor and the study center. The sponsor will prepare the manual for study drug management and submit it to the head of the study center.

(2) Storage and management of the study drug

The study drug storage manager will properly manage and store the study drug according to the above-described manual for study drug management, and will prepare study drug management records to identify the status of the use of the study drug.

(3) Collection of the study drug

The sponsor will collect leftover study drugs, empty boxes and unused study drugs together with the study drug management record (copy) for the subjects at an appropriate timing.

# 7. Examination and observation parameters

## 7.1 Procedures of subject registration

(1) Informed consent and definite diagnosis

[1] Informed consent from the subjects

The investigator or the subinvestigator will evaluate patients who have undergone topical medical therapy or surgical resection, and obtain informed consent in writing from those patients deemed eligible to participate in this study.

[2] Confirmation of eligibility of the subjects

After an informed consent has been obtained from a subject, the investigator or the subinvestigator will perform the scheduled dynamic CT and various tests to confirm complete cure within 56 days before registration, and will confirm that the subject satisfies the inclusion criteria and does not fall under any exclusion criteria.

[Test parameters regarding the inclusion criteria and exclusion criteria]

1） Dynamic CT images

2） Medical examination (symptoms/signs)

3） Blood pressure (dorsal position or sitting position)

4） Child Pugh classification

(Liver encephalopathy, ascites, serum bilirubin, serum albumin and prothrombin time)

5) HCV-RNA tests (quantitative)

6) HBs antigen tests (qualitative)

7） HCG tests (pregnant or not: premenopausal women only)

8） Hematology tests (platelet count, hemoglobin level)

(2) Subject registration

The investigator or the subinvestigator will enter necessary information on the subject registration form and will fax it to the subject registration center. The sponsor will confirm on the subject registration form received (Fax) that there are no problems in the eligibility of the subjects.

(3) Study drug assignment and start of study drug administration

The investigator or the subinvestigator will start administration of the study drug according to the assignment table prepared in advance.

## 7.2 Preparation of subject screening name list, etc.

The investigator or the subinvestigator will prepare a tabulated list (subject screening name list) of those patients who have received briefing about the contents of the study using written information for informed consent. Record the day on which the information regarding the study is given, the day on which informed consent is obtained, and the day on which study drug administration is started on the subject screening name list.

## 7.3 Subject backgrounds

Record the information listed below for the subjects for whom registration confirmation forms have been sent.

[1] Subject number

[2] Before complete cure: As of the time of diagnosis of HCC [the date and procedure of CT examination, diameter of the main tumor, number of tumors, first occurrence/first recurrence (timing of treatment of first occurrence in the case of first recurrence)]

[3] Radical therapy: Date of treatment, operative procedure for treatment (treatment method in the case of topical medical therapy), and the date of CT examination for confirmation of complete cure

[4] Previous history and complications

[5] Results of the tests on registration

[6] Information regarding registration (date of subject registration, date of informed consent, date of birth, gender)

[Flow of registration]

Subjects will be registered according to the following flow.

|  | 8 weeks (56 days) or more and 12 weeks (84 days) or less | 8 weeks (56 days) or less |  |
| --- | --- | --- | --- |

| Subjects |  |  |  |  |  |  |  |  |  |  |  |  |  |  |  |  |  |  |  |  |  |  |  |  |
| --- | --- | --- | --- | --- | --- | --- | --- | --- | --- | --- | --- | --- | --- | --- | --- | --- | --- | --- | --- | --- | --- | --- | --- | --- |
|  | Hepatocellular carcinoma of first occurrence or first recurrence | |  | Topical medical therapy or surgical resection |  |  |  |  | Briefing to subjects |  | Obtaining subjects’ consent |  |  |  |  |  |  |  |  |  |  |  | Liver biopsy/start of study drug administration |  |
|  |  |  |  |  |  |  |  |  |  |  |  |  |  |  |  |  |  |  |  |  |  |  |  |  |
|  |  |  |  |  |  |  |  |  |  |  |  |  |  |  |  |  |  |  |  |  |  |  |  |  |
| Investigator  or  Subinvestigator |  |  |  |  |  |  |  |  |  |  |  |  |  |  |  |  |  |  |  |  |  |  |  |  |
|  | Diagnosis | |  |  |  | Diagnosis (postoperative confirmation) | |  |  |  |  |  | Definite diagnosis | | | |  |  |  | Registration/confirmation of assignment | |  |  |  |
|  |  |  |  |  |  |  |  |  |  |  |  |  |  |  |  |  |  |  |  |  |  |  |  |  |
|  |  |  |  |  |  |  |  |  |  |  |  |  |  |  |  |  |  |  |  |  |  |  |  |  |
| Kowa Company, Ltd.  Pharmaceutical Development 2 |  |  |  |  |  |  |  |  |  |  |  |  |  |  |  |  |  |  |  |  |  |  |  |  |
|  |  |  |  |  |  |  |  |  |  |  |  |  |  |  | Confirmation of eligibility | |  | Subject registration |  | Assignment | |  |  |  |
|  |  |  |  |  |  |  |  |  |  |  |  |  |  |  |  |  |  |  |  |  |  |  |  |  |
|  | | | | | | | | | | | | | | | | | | | | | | | | |

[Study schedule]

Conduct examination/observation/investigation upon subject registration and during the study drug administration period according to the following schedule.

| Timing (week)  Parameter | | Registration (within 56 days before) | During study drug administration period | | | | | | | At the time of follow-up examination (Week 12 after completion of study drug administration) | At the time of study discontinuation |
| --- | --- | --- | --- | --- | --- | --- | --- | --- | --- | --- | --- |
|  |  |  | Start of administration  (within 14 days before) | Weeks 4, 28, 52, 76 | Weeks 8, 32, 56, 80 | Weeks 12, 36, 60, 84 | Weeks 16, 40, 64, 88 | Weeks 20, 44, 68, 92 | Weeks 24, 48, 72, 96 |  |  |
| Study drug administration | | − |  | | | | | | | − | − |
| Examination of compliance status | | − | − | ● | ● | ● | ● | ● | ● | − | − |
| Gene expression profile | | − | ⦿ | − | ⦿ | − | − | − | − | − | − |
| TGF-α | | − | ⦿ | − | ⦿ | ● | − | − | ● | − | ✰ |
| Liver drug concentration | | − | ⦿ | − | ⦿ | − | − | − | − | − | − |
| Plasma drug concentration | | − | ⦿ | − | ⦿ | − | − | − | ● | ● | − |
| Urinary drug concentration | | − | ⦿ | − | ⦿ | − | − | − | − | − | − |
| Abdominal imaging diagnosis | | ● | − | − | − | ● | − | − | ● | ● | ✰ |
| Medical examination | | ● | ● | ● | ● | ● | ● | ● | ● | ● | ✰ |
| Vital signs, etc. | | ● | ● | ● | ● | ● | ● | ● | ● | ● | ✰ |
| Child-Pugh classification | Encephalopathy/ascites | ● | − | − | − | − | − | − | − | − | − |
|  | T-Bil, ALB, PT | ● | − | − | − | − | − | − | − | − | − |
| HBs antigen | | ● | − | − | − | − | − | − | − | − | − |
| HCG tests (premenopausal women only) | | ● | − | − | − | − | − | − | ● | − | − |
| Hematology tests  (platelet count, hemoglobin level) | | ● | − | − | − | − | − | − | − | − | − |
| Hematology tests | | − | ● | ● | ● | ● | ● | ● | ● | ● | ✰ |
| Blood biochemistry tests | | − | ● | ● | ● | ● | ● | ● | ● | ● | ✰ |
| Urinary tests (fresh urine) | | − | ● | ● | ● | ● | ● | ● | ● | ● | ✰ |
| Blood pressure parameters | | − | ● | − | − | − | − | − | ★ | − | ✰ |
| HCV-RNA (quantitative) | | ● | − | − | − | − | − | − | ★ | − | ✰ |
| HCV-RNA (genotype analysis) | | − | ● | − | − | − | − | − | − | − | − |
| Tumor markers | | − | ● | − | − | ● | − | − | ● | ● | ✰ |
| Fibrosis marker | | − | ● | − | − | − | − | − | ★ | − | ✰ |
| Bone densitometry (DXA，lumbar vertebrae) | | − | ⭘ | − | − | − | − | − | ★ | − | ✰ |
| Endoscopy | | − | ⭘ | − | − | − | − | − | ★ | − | ✰ |
| ECG | | − | ⭘ | − | − | − | − | − | ● | − | ✰ |
| Echocardiography | | − | ⭘ | − | − | − | − | − | ★ | − | ✰ |
| Pulse wave examination | | − | ⭘ | − | − | − | − | − | ★ | − | ✰ |
| Fundus examination | | − | ⭘ | − | − | − | − | − | ★ | − | ✰ |
| Adverse events | | − |  | | | | | | | |  |

⦿: The day before the start of administration and the day of evaluation at Week 8

⭘: Examination will be conducted during the period between the end of radical therapy and the start of study drug administration.

★: Examination will be conducted at 48-week intervals.

✰: Examination will be conducted upon discontinuation of the study if possible.

Note 1: The acceptable range of deviation from the scheduled examination, observation and evaluation during the study period is +28 days for gene expression profile (Week 8) and pharmacokinetics (Week 8); ±35 days for abdominal imaging diagnosis, HCV-RNA, HCG tests, tumor markers, fibrosis markers, bone densitometry, endoscopy, ECG, echocardiography，pulse wave examination, and fundus examination; and ±7 days for other parameters.

Note 2: Subjects need to be hospitalized on the day of the start of administration and on the day of evaluation at Week 8. Liver biopsy will be performed and blood samples and accumulated urine samples will be collected on the day before the start of administration, with administration of the study drug to start 24 hours after the operation. On the day of evaluation at Week 8, subjects will visit the hospital without taking the study drug, and undergo liver biopsy 4 hours after administration. Blood samples will be collected 4 and 8 hours after administration. Urine samples will be accumulated for 24 hours after administration.

Note 3: Blood pressure parameters will be assessed on the day of the start of administration and at Week 48 and Week 96 of administration. If the systolic blood pressure exceeds 140 mmHg or the diastolic blood pressure exceeds 90 mmHg in two or more consecutive tests, the parameters will be determined on the second or subsequent tests. Determination of creatinine clearance, one of the blood pressure parameters, under increased blood pressure will be conducted to the extent possible. Creatinine clearance will be determined using urine samples collected at home at the timings other than the day of the start of administration (i.e., Week 48 and Week 96 of administration, and examination of blood pressure parameters in the presence of increased blood pressure).

Note 4: See Table 1, Table 2 and Table 3 at the end of this document for details about the contents of the tests.

## 7.4 Gene expression profile

(1) Liver biopsy

Perform liver biopsy to collect liver tissue samples for determinations of the liver gene expression profile drug concentrations.

[1] Liver biopsy procedure

Perform liver biopsy after the absence of the risk of haemorrhage has been confirmed in advance by the blood coagulation test, etc. Under local anesthesia, needle biopsy is performed with the aid of ultrasonography using a Bard Monopty automatic biopsy needle (Max Core). The specification is as follows: depth of insertion; 22 mm, external diameter; 16 G, needle length; 160 mm. The upper limit for the amount of liver tissue samples to be collected per day for determination of the gene expression profile and drug concentrations in the liver will be 20 mg, respectively. The patients will remain at rest for a while after liver biopsy. They will be hospitalized on the days of examination.

[2] Timing of liver biopsy and the acceptable range

On the day before the start of study drug administration and on the day of evaluation at Week 8 after the start of administration (4 hours after administration)

- 4 hours after administration: ±30 minutes

If a deviation from the designated timing of sample collection is within the acceptable range, the sample will be deemed as collected at the designated timing.

If the investigator or the subinvestigator deems that liver biopsy cannot be performed on the designated day of evaluation at Week 8 due to the subject’s health condition, the liver biopsy can be postponed by up to 28 days. In such cases, the period of Step I [the period of administration of two doses, i.e., 300 mg/day (twice daily) and 600 mg/day (twice daily)] will be extended until completion of liver biopsy.

[Rationale for timing of liver biopsy]

The rationale for the timing of liver biopsy is described, as the rationale for selection of the administration period for determination of the gene expression profile, in the section of “Rationale” under “4.4.(3) Study drug administration period” for determination of the gene expression profile.

From the results of the phase I single administration study, the appropriate timing of liver biopsy on the day of evaluation at Week 8 of study drug administration was deemed to be 4 hours after administration, which is close to the time point of the C_max_ of unchanged NIK-333.

(2) Determination of the gene expression profile in the liver

The details of the procedure of determination of the gene expression profile are described in the “Manual for determination of the gene expression profile of NIK-333.”

[1] Procedure for specimen processing

Collect a liver tissue sample and remove blood using a piece of physiolocial saline-soaked filter paper. Then transfer the sample to a sample tube containing RNAlater (Ambion) and immerse it in the solution at 4°C at least for a night. Take the liver tissue sample out from the RNAlater within 3 days, transfer it to a new sample tube, and preserve in liquid nitrogen. After that, subject the liver tissue sample to RNA extraction and purification within 14 days, and then freeze and store at -80°C until determination of the gene expression profile.

[2] Determination of gene expression profile in the liver

The gene expression profile in the liver tissue will be determined before and after administration of NIK-333 using the microarray of Kanazawa University (KANAZAWA Liver Chip 10K).

Subject genes shown by the above analysis to be potential drug response genes to further analysis by the Real-Time RT-PCR method.

[3] Preservation and disposal of the specimens after determination of the gene expression profile

Store the RNA samples remaining after determination of the gene expression profile at the facility undertaking determination and analysis of gene expression profile (Disease Control and Homeostasis, Graduate School of Medicine, Kanazawa University) until the day when 5 years have passed after the date of manufacturing approval for a drug related to the study drug concerned (or 3 years after the day on which discontinuation of development is decided, if development of the drug is discontinued) or until the day of completion of re-examination, which ever later. At the end of the storage period, the study investigator of the facility undertaking determination and analysis of gene expression profile will discard the samples concerned.

(3) Determination of the gene expression profile in peripheral blood

[1] Timing of blood sample collection and the acceptable range

On the day before the start of study drug administration and on the day of evaluation at Week 8 after the start of administration (4 and 8 hours after administration).

- 4 hours after administration: ±30 minutes

- 8 hours after administration: ±30 minutes

[Rationale for timing of blood sample collection]

Blood samples for determination of the gene expression profile in peripheral blood will be collected 4 hours after study drug administration, the same timing as determination of the gene expression profile in the liver. The blood samples for determination of the gene expression profile in peripheral blood will also be collected 8 hours after administration to monitor the time-course change of the gene expression profile after study drug administration. The timing of 4 hours after study drug administration is close to the time point of the C_max_ of unchanged NIK-333, and the timing of 8 hours after study drug administration corresponds with the elimination phase of unchanged NIK-333 and which is close to the time point of the C_max_ of NIK-333 lipids form.

[2] Procedure for specimen processing

Collect blood samples using the PAXgene Blood RNA vacuum blood collection tube (2.5 mL×2). Extract, purify and concentrate RNA using the PAXgene Blood RNA Kit (QIAGEN). Then, freeze and store samples at -80°C.

[3] Shipment of the specimens

The study center will send the RNA samples in the frozen state to the facility undertaking determination of the gene expression profile in peripheral blood. The quality of RNA samples after shipment will be confirmed using a bioanalyzer at the facility undertaking determination of the gene expression profile in peripheral blood.

[4] Determination of the gene expression profile in peripheral blood

The gene expression profile in peripheral blood will be determined before and after administration of NIK-333 using AceGene®Premium (DNA Chip Research Inc.).

Subject genes shown by the above analysis to be potential drug response genes to further analysis by the Real-Time RT-PCR method.

[5] Preservation and disposal of the specimens after determination of the gene expression profile

Handle the RNA samples extracted from peripheral blood in the same way as the RNA samples extracted from the liver tissue as described in the section of “7.4 (2), [3] Preservation and disposal of the specimens after determination of the gene expression profile.”

## 7.5 Safety

(1) Indicators for safety evaluation

[1] Examinations for confirming recurrence

The investigator or the subinvestigator will perform imaging diagnosis by abdominal ultrasonography (US) and dynamic CT or dynamic MRI at 12-week intervals during the study drug administration period, as well as at the time of follow-up examination. If recurrence is suspected based on any other findings (such as gradual increase of tumor markers), imaging diagnosis by dynamic CT or dynamic MRI will be performed as needed.

[2] Other examinations for confirming recurrence

The investigator will precisely look for the recurrence of HCC by determining the levels of tumor markers during the study drug administration period. If recurrence is suspected by this determination, perform imaging diagnosis by dynamic CT or dynamic MRI as needed.

Tumor markers tests:

Determine AFP, AFP-L3 and PIVKA-II at 12-week intervals from the day of the start of study drug administration (within 14 days before the start) until the last day of study drug administration, and as well as at the time of follow-up examination.

If the study has been discontinued, AFP, AFP-L_3_ and PIVKA-II will be tested to the extent possible.

[3] Examinations related to the underlying disease

The investigator will perform HCV-RNA tests and fibrosis markers tests so as to examine the underlying disease during the study drug administration period.

1) HCV-RNA tests (quantitative)

Perform HCV-RNA tests at the time of registration (within 56 days before administration) and at 48-week intervals from the start of study drug administration until the end of study drug administration. Conduct genotype analysis on the day of the start of administration.

If the study is discontinued, perform HCV-RNA test to the extent possible.

2) Fibrosis markers tests

Perform hyaluronic acid test at 48-week intervals from the start of study drug administration (within 14 days before the start of administration) until the end of study drug administration.

If the study has been discontinued, hyaluronic acid will be tested to the extent possible.

[4] Medical examination

Examine symptoms and signs at 4-week intervals from the start of study drug administration (within 14 days before the start of administration) until the end of study drug administration, and at the time of follow-up examination.

If the study has been discontinued, symptoms and signs will be examined to the extent possible.

[5] Vital signs, etc.

Take body temperature (axillary), blood pressure (systolic/diastolic), pulse rate (dorsal position or sitting position) and body weight at 4-week intervals from the start of study drug administration (within 14 days before the start of administration) until the end of study drug administration, and at the time of follow-up examination.

If the study has been discontinued, body temperature (axillary), blood pressure (systolic/diastolic), pulse rate (dorsal position or sitting position) and body weight will be determined to the extent possible.

[6] Laboratory tests

Collect blood and urine samples and determine the following parameters at 4-week intervals from the start of study drug administration (within 14 days before the start of administration) until the end of study drug administration, and at the time of follow-up examination.

If the study has been discontinued, blood and urine samples will be collected and the following parameters will be determined to the extent possible.

1) Hematology tests: White blood cell count, red blood cell count, hemoglobin level, hematocrit, platelet count

2) Blood biochemistry tests: Total protein, serum albumin, serum bilirubin, total cholesterol, LDL cholesterol, HDL cholesterol, triglyceride, blood glucose, urea nitrogen, Cl, Na, K, Ca, ALP, AST (GOT), ALT (GPT), LDH, γ-GTP, choline esterase, serum creatinine, A/G ratio, free fatty acid, β2-microglobulin

3) Urinary tests (fresh urine): Qualitative (glucose, protein, urobilinogen, ketone body, occult blood), urinary albumin (converted to creatinine), urinary sediment (red blood cells, white blood cells, epithelial cells, casts, others), β2-microglobulin

[7] Blood pressure parameters

Collect blood and urine samples and determine the following parameters at 48-week intervals from the start of study drug administration until the end of study drug administration. If the systolic blood pressure exceeds 140 mmHg or the diastolic blood pressure exceeds 90 mmHg on two or more successive measurements, blood pressure parameters will be determined at the time of second or subsequent examination. However, determination of creatinine clearance, one of the blood pressure parameters, under increased blood pressure will be conducted to the extent possible.

1) Blood biochemistry tests: Renin activity, aldosterone, cortisol, uric acid, FT_3_, FT_4_, TSH, angiotensin II, hANP, BNP, catecholamine, growth hormone, dehydroepiandrosterone sufate (DHEAS)

2） Urinalyses (accumulated urine): creatinine clearance (if possible)

[8] Bone densitometry

Determine bone density of lumbar vertebrae (L_2_ to L_4_) with DXA before the start of study drug administration (period after radical therapy and before administration of the study drug) and at 48-week intervals until the day of the end of study drug administration.

If the study has been discontinued, bone density of lumbar vertebrae will be tested using DXA to the extent possible.

[9] Endoscopy

Conduct endoscopy of the stomach and esophagus before the start of study drug administration (period after radical therapy and before administration of the study drug) and at 48-week intervals until the day of the end of study drug administration.

If the study has been discontinued, endoscopy of the stomach and esophagus will be performed to the extent possible.

[10] ECG

Perform ECGs (12-lead) before the start of study drug administration (period after radical therapy and before administration of the study drug) and at 24-week intervals until the day of the end of study drug administration.

If the study has been discontinued, perform ECGs (12-lead) to the extent possible.

[11] Echocardiography

Perform echocardiography before the start of study drug administration (period after radical therapy and before administration of the study drug) and at 48-week intervals until the day of the end of study drug administration.

If the study has been discontinued, perform echocardiography to the extent possible.

[12] Pulse wave examination

Perform pulse wave examination before the start of study drug administration (period after radical therapy and before administration of the study drug) and at 48-week intervals until the day of the end of study drug administration.

If the study has been discontinued, perform pulse wave examinations to the extent possible.

[13] Fundus examination

Perform fundus examination before the start of study drug administration (period after radical therapy and before administration of the study drug) and at 48-week intervals until the day of the end of study drug administration.

If the study has been discontinued, perform fundus examination to the extent possible.

(2) Adverse events

Adverse events are defined as any unfavorable or unintended signs (including abnormal variations of laboratory test values), symptoms or diseases observed after administration of the study drug, irrespective of the causal relationship with the study drug concerned.

Recurrent HCC and other secondary cancers observed during the study period will be regarded as adverse events and analyzed separately from other adverse events.

If any adverse events have occurred, the following information will be recorded in detail. At that time, the severity of the symptom, outcome, causal relationship with the study drug, and comments will be assessed according to the standards presented below.

Adverse events for which causal relationship with the study drug cannot be definitely ruled out (causal relationship: 1 to 3) will be handled as adverse drug reactions.

[1] Name of adverse event

[2] Timing of onset and timing of outcome

[3] Degree of symptoms (severity: mild/moderate/severe; seriousness: not serious/serious)

[4] Study drug administration (continued/discontinued/completed)

[5] Whether treated or not

[6] Outcome (disappeared/remitted/unchanged/exacerbated/death/unknown)

[7] Causal relationship with the study drug (related/probab/related/possibly related/not related)

[8] Details about treatment and clinical course

[9] Details about causal relationship with the study drug

[Degree of symptoms]

| Severity | Mild | Easily bearable and not interfering with daily life |
| --- | --- | --- |
|  | Moderate | Interfering with usual activities |
|  | Severe | Disabling usual activities |
| Seriousness | Not serious | Adverse events other than serious adverse events |
|  | Serious | Serious adverse events (See 9.3 Reporting adverse events) |

[Outcome ]

| 1 | Disappeared | Symptoms/signs have disappeared, and test values have become normal or recovered to the pre-administration levels. |
| --- | --- | --- |
| 2 | Remitted | The severity has decreased or symptoms/signs have shown a tendency toward improvement. |
| 3 | Unchanged | There are no changes in symptoms · signs and test values. |
| 4 | Exacerbated | Symptoms/signs and test values have become exacerbated. |
| 5 | Death | The patient died. |
| 6 | Unknown | Symptoms/signs or test values are unknown because the patient did not show up, etc. |

[Causal relationship with the study drug]

| 1 | Related | Clinical symptoms including abnormal laboratory test results have occurred at a temporally reasonable timing in relation to the study drug administration, and cannot be explained by concurrent diseases or other concomitant drugs or chemical substances. Clinically plausible responses are seen after discontinuation (interruption) of study drug administration. The symptoms can be reproduced pharmacologically or phenomenologically definitely by reasonable re-administration, if necessary. |
| --- | --- | --- |
| 2 | Probably Related | Clinical symptoms including abnormal laboratory test results have occurred at a temporally reasonable timing in relation to study drug administration, and their association with concurrent diseases or other concomitant drugs or chemical substances can probably be ruled out. A clinically reasonable response is observed after discontinuation (interruption) of study drug administration. Information regarding re-administration is not required for this definition. |
| 3 | Possibly related | Clinical symptoms including abnormal laboratory test results have occurred at a temporally reasonable timing in relation to study drug administration, and can be explained by concurrent disease or other concomitant drugs or chemical substances. Information regarding discontinuation of study drug administration is not available or unclear. |
| 4 | Not related | Definite factors other than the study drug (such as physiological factors, environmental factors, restraints, exercise, measurement errors, etc.) are present, and the absence of causal relationship with the study drug can be reasonably explained with sufficient information. |

## 7.6 Pharmacokinetics

(1) Drug concentrations in the liver

[1] Timing of liver biopsy and the acceptable range

Described in the section of “7.4 (1) Liver biopsy.”

[2] Procedure for specimen processing

Collect liver tissue samples for determination of drug concentrations at the same time as liver biopsy prescribed in the section of “7.4 (1) Liver biopsy.” Remove blood from the liver tissue samples collected using a piece of physiological saline-soaked filter paper, with the tissue weight determined using a semi-micro balance. Immerse the tissue samples in a polypropylene container containing 99.5% ethanol and purge the residual space with argon gas. After that, seal and store the container protected from light in a freezer at -80°C.

[3] Shipment of the specimens

The study center will send the liver tissue samples for determination of drug concentrations in the frozen state to the facility undertaking determination of drug concentrations.

[4] Procedure of drug concentration determination

To determine the concentrations of unchanged NIK-333 and NIK-333 lipids form in the liver, extract liver samples, obtain derivatives and determine the concentrations by liquid chromatography-atmospheric pressure chemical ionization-tandem mass spectrometry (LC-APCI-MS/MS).

(2) Drug concentration in plasma

[1] Timing of blood sample collection and the acceptable range

- On the day before the start of study drug administration and on the day of evaluation at Week 8 after the start of administration (4 and 8 hours after administration)

- 4 hours after administration: ±30 minutes

- 8 hours after administration: ±30 minutes

- Weeks 24, 48, 72 and 96 after the start of study drug administration

- 4 hours after administration: ±30 minutes

If a deviation from the blood sample collection time is within the acceptable range, the sample will be deemed as being collected at the designated time.

[Rationale for timing of blood sample collection]

On the day before the start of administration and at Week 8 after the start of administration when liver biopsy is performed and the subject is hospitalized for monitoring, collect blood samples at 4 hours after administration, which is close to the time point of the C_max_ of unchanged NIK-333, and at 8 hours after administration, which is equivalent to the elimination phase of unchanged NIK-333 and close to the time point of the C_max_ of NIK-333 lipids form.

From Week 8 after the start of administration, fixed-point determination will be made only at 4 hours after administration to confirm that NIK-333 and NIK-333 lipids form have reached a steady state during the administration period of 48 weeks or more. Determination of NIK-333 lipids form will provide essential information to be taken into consideration in the cases in which any changes are seen in the blood concentrations of NIK-333, because it is the “strage form” of NIK-333 and present in blood at higher concentrations than NIK-333.

- Follow-up examination of pharmacokinetics

Week 12 after the end of study drug administration

[Rationale for timing of blood sample collection]

To confirm elimination of the study drug from plasma after repeated administration of NIK-333

[2] Procedure of blood sample collection and amount of blood samples

At each time point for blood sample collection, collect 5 mL of blood from the cubital vein into a vacuum blood collection tube using EDTA-2Na as an anticoagulant, immediately mix thoroughly and centrifuge (4°C, 3,000 rpm, 15 minutes) to obtain the plasma. Dispense the plasma into 5 tubes of 500 μL each. Purge the residual space of 4 of these tubes with argon gas, and seal and store the tubes protected from light in a freezer at -80°C. Store the remaining 1 tube for determination of TGF-α.

At the time of follow-up examination of pharmacokinetics, collect 2.5 mL of blood, but do not collect any blood samples for the determination of TGF-α.

[3] Shipment of the specimens

The study center will send the plasma samples for determination of drug concentrations in the frozen state to the facility undertaking determination of drug concentrations.

[4] Procedure of determination of the concentrations of unchanged NIK-333 and NIK-333 lipids form

To determine the concentrations of unchanged NIK-333 and NIK-333 lipids form in plasma, extract plasma samples, obtain derivatives and determine the concentrations by liquid chromatography-atmospheric pressure chemical ionization-tandem mass spectrometry (LC-APCI-MS/MS).

[5] Determination of metabolites in plasma

The sponsor will determine the concentration of NIK-333-26, a presumed metabolite,in plasma using the plasma samples remaining after determination of unchanged NIK-333 and NIK-333 lipids form in plasma. However, the metabolite can be determined only when the subject’s additional consent has been obtained in advance. The chemical name and structural formula of the presumed metabolite are shown below.

- NIK-333-26

Chemical name: (6*E*,10*E*)-3,7,11,15-tetramethyl-6,10,14-hexadecatrienoic acid

Structural formula:

[6] Procedure of determination of the concentrations of metabolite

To determine the concentration of NIK-333-26, extract the remaining plasma samples, obtain derivatives and determine the concentrations in plasma by liquid chromatography-atmospheric pressure chemical ionization-tandem mass spectrometry (LC-ESI-MS/MS).

(3) Determination of drug concentrations in urine

[1] Timing of urine sample collection

Accumulate urine samples at the time of liver biopsy. In other words, accumulate urine samples for 24 hours from the day before the start of study drug administration (day of liver biopsy), and for 24 hours from immediately after administration at Week 8 of administration.

[2] Procedure of urine sample collection and amount of urine samples

Place each single voided urine sample obtained by natural micturition in a light-protected glass container and store in a refrigerator (4°C). Combine single voided urine samples and determine the total urine volume with a glass measuring cylinder. Wash the containers with the same volume of methanol and stir the sample thoroughly. Then, dispense 200 mL each of the sample into 3 designated containers. Purge the residual space of the designated container with argon gas, and store the containers protected from light in a freezer at -80°C.

[3] Shipment of the specimens

The study center will send 3 containers of the urine samples in the frozen state to the facility undertaking determination of drug concentrations for determination of drug concentrations. The facility undertaking determination of drug concentrations will keep one of the containers.

[4] Procedures for drug concentration determination

To determine the urinary concentrations of unchanged NIK-333, extract urine samples with diethyl ether/2-propanol, and determine the concentrations by liquid chromatography-ultraviolet spectrophotometry (LC-UV).

[5] Determination of metabolites in urine

The sponsor will determine NIK-333 metabolites in urine when deemed necessary. If a procedure for determining NIK-333 metabolites is established in the future, metabolites can be determined after an additional consent is obtained from the subjects.

## 7.7 Exploratory biomarkers (TGF-α concentration in plasma)

In addition to determination of the gene expression profiles in the liver and peripheral blood, determine concentrations of TGF-α in plasma to assess its potential as a biomarker associated with gene expression. Moreover, monitor the changes of TGF-α concentrations in plasma from Week 8.

[1] Timing of blood collection

- On the day before the start of study drug administration and on the day of evaluation at Week 8 after the start of administration (4 and 8 hours after administration)

- 4 hours after administration: ±30 minutes

- 8 hours after administration: ±30 minutes

- Weeks 12, 24, 36, 48, 60, 72, 84 and 96 after the start of study drug administration

- 4 hours after administration: ±30 minutes

[Rationale for timing of blood sample collection]

In the same way as determination of gene expression profile in peripheral blood, determine TGF-α in plasma on the day before the start of administration, at Week 8 after the start of administration (4 and 8 hours after administration), and subsequently at Weeks 12, 24, 36, 48, 60, 72, 84 and 96 after the start of study drug administration to monitor the changes over time and to explore the potential of TGF-α as a biomaker.

[2] Procedure of blood sample collection and amount of blood samples

<<Weeks 12, 36, 60 and 84 after the start of administration>>

At each time point for blood sample collection, collect 1 mL of blood from the cubital vein into a vacuum blood collection tube using EDTA-2Na as an anticoagulant, immediately mix thoroughly and centrifuge (4°C, 3,000 rpm, 15 minutes) to obtain the plasma. Store the plasma, 500 μL, in a freezer at -80°C.

<<Day before the start of study drug administration and at Weeks 8, 24, 48, 72 and 96 after the start of administration>>

At each time point for blood sample collection, collect 5 mL of blood from the cubital vein into a vacuum blood collection tube using EDTA-2Na as an anticoagulant, immediately mix thoroughly and centrifuge (4°C, 3,000 rpm, 15 minutes) to obtain the plasma. Dispense the plasma into 5 tubes of 500 μL each. Store one of them in a freezer at -80°C and the remaining 4 tubes as the samples for determination of drug concentrations in plasma.

[3] Shipment of the specimens

The study center will send the plasma samples for TGF-α determination in the frozen state to the facility undertaking determination of TGF-α.

[4] Procedure of determination

Determine the concentrations of TGF-α in plasma by ELISA.

## 7.8 Acceptable range of deviations from the dates of examination, observation and evaluation

The acceptable range of deviation from the scheduled examination, observation and evaluation on the day of the start of administration is the period from completion of radical therapy until the start of study drug administration for bone densitometry, endoscopy, ECG, echocardiography, pulse wave examination and fundus examination and within 14 days before the designated date for other parameters.

The acceptable range of deviation from the scheduled examination, observation and evaluation during the study drug administration period is +28 days for gene expression profile (Week 8), TGF-α (Week 8), and liver, plasma and urinary pharmacokinetics (all are at Week 8), ±35 days for abdominal imaging diagnosis, HCV-RNA, HCG tests, tumor markers, fibrosis markers, bone densitometry, endoscopy, ECG, echocardiography, pulse wave examination and fundus examination, and ±7 days for other parameters.

The acceptable range of deviation from the scheduled examination, observation and evaluation at follow-up examination (Week 12 after the end of administration) is +28 days.

# 8. Subjects’ treatment compliance and subject management

## 8.1 Subjects’ treatment compliance

The investigator or the subinvestigator will instruct subjects to make sure to bring the remaining study drugs when they visit the outpatient clinic. The investigator or subinvestigator will confirm and record the subjects’ treatment compliance status on the basis of the remaining drugs.

## 8.2 Subject management

The investigator or the subinvestigator will provide guidance to the subjects regarding the following matters.

[1] Health management

1) Instruct the subjects to immediately report any abnormalities observed during the study period to the investigator or the subinvestigator, to visit the hospital as needed, and to receive medical examination and treatment.

2) Premenopausal women will be instructed to make sure to practice contraception during the study period. Conduct the HCG test at 24-week intervals to check for pregnancy.

[HCG tests]

During the study period, test serum HCG (human chorionic gonadotropin) at the time of subject registration and at 24-week intervals during the period from the start of study drug administration until the day of the end of study drug administration. In addition, examine serum HCG at the time of completion/discontinuation of study drug administration if possible. If the study has been discontinued, test serum HCG to the extent possible.

[Rationale]

NIK-333 has been confirmed to be teratogenic and induce miscarriage, and administration to pregnant or potentially pregnant women must be avoided.

[2] Use of other drugs

1) Instruct the subjects to refrain from taking vitamin A products and nutritional supplements of which labeled components include vitamin A (retinol) and vitamin K during the study period.

2) Instruct the subjects to consult with the investigator or the subinvestigator in advance and to follow their directions regarding the intake of other drugs and nutritional supplements during the study period.

## 8.3 Status of completion of the study

The investigator or the subinvestigator will confirm the status of study completion of each subject, and record it in the case report form. If the study has been discontinued, record the date of study discontinuation, reasons for study discontinuation, details about study discontinuation, treatment and course, and follow-up examinations. If the study has been discontinued, follow the procedure in “10.2. Procedures of discontinuation.”

# 9. Measures taken to cope with adverse events and reporting procedures

## 9.1 Securing subjects’ safety

The investigator or the subinvestigator will pay due attention to the safety of subjects, give appropriate treatment to the subjects, and take appropriate measures when any adverse events have occurred.

## 9.2 Follow-up examination

When any adverse events for which causal relationship with the study drug cannot be ruled out are observed at the end of the study, when the clinical study is discontinued due to any safety problems, including the occurrence of adverse events, and when the investigator or the subinvestigator has found it necessary, follow-up examination will be conducted and the results will be recorded until recovery from the symptoms is observed, or until the symptoms become stable and the investigator medically judges that further follow-up is no longer necessary. If a subject failed to show up for follow-up visits for any reasons, such as death, the reason will be recorded.

## 9.3 Reporting of adverse events

The investigator or the subinvestigator will report all adverse events encountered to the sponsor. Immediately report the emergence of any serious adverse events listed below to the head of the study center and the sponsor by telephone or fax irrespective of the causal relationship with the study drug. After that, the investigator will immediately report the events to the head of the study center and the sponsor in writing. When additional information has been requested by the head of the study center and the sponsor, the investigator will provide such information.

[Serious adverse events]

[1] Resulting in death

[2] Life-threatening

[3] Requiring inpatient hospitalization or prolongation of existing hospitalization for treatment

[4] Resulting in persistent or significant disability/incapacity

[5] Leading to a congenital anomaly

[6] Other medically important states

The adverse events listed above include those attributable to adverse reactions to the study drug, or to infections which are suspected to be attributable to the use of the study drug.

[Emergency safety center]

Bellsystem 24, Inc.

Tel. 0120-490-773 Fax. 0120-490-774

Reception hours: Around the clock (24 hours)

## 9.4 Reporting to the regulatory authority

When the sponsor has concluded that any report of serious adverse events received from the investigator falls under the definition of Article 273 of the Enforcement Regulations for the Pharmaceutical Affairs Law, the sponsor will report the adverse events to the regulatory authority according to the provision of the article. Moreover, the adverse events will be reported to the heads and investigators of other study centers.

# 10. Discontinuation criteria and procedures of discontinuation

## 10.1 Discontinuation criteria

When any of the following discontinuation criteria is met, the trial for an individual subject or the entire study will be discontinued pursuant to the “10.2 Procedures of discontinuation.”

(1) Rules regarding discontinuation of the study for individual subjects

When any of the following criteria is met, the trial for the subject concerned will be discontinued.

[1] When the investigator or the subinvestigator has confirmed the recurrence of liver cancer.

[2] When the investigator or the subinvestigator has confirmed the occurrence of a secondary cancer

[3] When the patient died

[4] When the investigator or the subinvestigator has judged that it is difficult to continue the trial

[5] When a violation of “5.2 Inclusion criteria (1),(2),(3),(5)-1)” has been indentified after initiation of administration

[6] When the subject is found to be pregnant

[7] When the subject has withdrawn his or her consent

[8] When the subject failed to show up for follow-up visits due to transfer to another hospital or for other reasons

[9] When the subject took any of the drugs listed in 1 or 2 of “11. Treatment for subjects, (2) Prohibited or caution-required concomitant therapy, (1) Prohibited concomitant drugs and prohibited concomitant food”

[10] Other cases in which the investigator or the subinvestigator has concluded that continuation of the study is difficult

(2) Rules regarding discontinuation of the study

Discontinue the study when any one of the following criteria is met.

[1] When the Institutional Review Board has stated an opinion that continuation of the clinical study is inappropriate.

[2] When the investigator has concluded that it is inappropriate to continue the clinical study

[3] When the study center has violated the Ordinance GCP, study protocol or clinical study contract, and the sponsor has concluded that the violation interferes with the proper conduct of the clinical study

(3) Rules regarding discontinuation of the study for reasons other than those listed above

Discontinue the study when the sponsor has judged that there is no alternative but to discontinue the study for the reasons listed below.

[1] When any events which become a problem in the conduct of the clinical study have been observed in non-clinical studies, and the sponsor has concluded that it is difficult to continue the study

[2] When any important reports regarding safety have been obtained from the clinical study concerned or from other sources of information, and the sponsor has concluded that it is difficult to continue the study

[3] When the sponsor has discontinued the study for any other reasons

## 10.2 Procedures of discontinuation

(1) Procedures for coping with subjects

If the clinical study is discontinued, the investigator or the subinvestigator will immediately discontinue administration of the study drug to the subject concerned, and explain to the subject the fact that the study will be discontinued as well as the reasons of discontinuation. At that time, necessary examinations will be conducted, and the fact that the study is discontinued, the date of discontinuation, reasons of discontinuation, and details (outcome, treatment, etc.) will be recorded.

The subjects who have discontinued the study due to safety problems, such as the occurrence of adverse events, will be followed up to the extent possible, and the details will be recorded.

In the cases of discontinuation of the trial for a subject without involving death or recurrence of HCC, investigate whether or not HCC has recurred in the subject during the period from the start of study drug administration until Week 96 following the start after obtaining an additional consent from the subject.

(2) Reporting of discontinuation by the investigator

When the investigator has decided to discontinue the clinical study, he/she will immediately report that to the sponsor and the head of the study center.

(3) Reporting of discontinuation by the sponsor

The sponsor, after deciding to discontinue the clinical study, will immediately report that to the head of the study center and the investigator.

# 11. Treatment for subjects

Record any treatment other than drug therapy with the study drug given to the subjects, (e.g., a concomitant drug).

(1) Permitted treatment method

Treatments during the study drug administration period

During Step I, to the extent possible, avoid changing the type and dosage regimen of the drug, or discontinuing administration of the drug which the subject has been taking before the start of the study drug administration because gene expression may be affected. If concomitant use of any other drugs has become necessary, long-term administration should be avoided to the extent possible.

In Step II, there are no restrictions regarding the treatments other than those targeted to HCC, unless prohibited concomitant drugs and caution-required concomitant drugs are used.

However, if any other drugs are used during the study drug administration period, the name of the drug used, daily dose, route of administration, administration period and objectives of the use should be recorded.

(2) Prohibited or caution-required treatment methods

Treatments with the following drugs are designated as prohibited or caution-required concomitant treatments because they may interfere with efficacy evaluation of the subjects or assurance of safety. Moreover, concomitant use of both prohibited concomitant drugs and caution-required concomitant drugs is prohibited during the period from the day of the start of study drug administration until Week 8 after the start of administration because gene expression may be affected.

[1] Prohibited concomitant drugs and prohibited concomitant food: Administration and consumption of the following drugs and food are prohibited during the study drug administration period.

|  | Drug classification | Generic name | Brand name |
| --- | --- | --- | --- |
| 1 | Other study drugs | − | − |
| 2 | Anticancer drugs | All anticancer drugs including 5FU, MMC, DXR and tretinoin | |
| 3 | Natural interferons | Interferon α | OIF, Sumiferon |
|  |  | Interferon β | IFN β, Feron |
| 4 | Genetically-modified interferons | Peginterferon-α-2a | Pegasys |
|  |  | Interferon α-2b | Intron A |
|  |  | Peginterferon-α-2b | Pegintron |
|  |  | Interferon alfacon-1 | Advaferon |
|  |  | Interferon β-1a | Avonex |
|  |  | Interferon β-1b | Betaferon |
| 5 | Vitamin K_2_ | Menatetrenone | Glakay, Kaytwo |
| 6 | Retinoid | Etretinate | Tigason |
| 7 | Vitamin A | Cod-liver oil | Cod-liver oil |
|  |  | Retinol palmitate | Chocola A |
| 8 | Antiviral drugs | Ribavirin | Rebetol, Copegus |
| 9 | Supplements containing vitamin A (Lyc-O-Mato Power E, etc.) (health food) | | |
| 10 | Supplements containing vitamin K (health food) | | |

Concomitant administration of the drugs containing the above components will also be prohibited.

[Rationale]

1-5, 10: Because they interfere with or may interfere with evaluation of the efficacy

6: Because they may induce symptoms of adverse reactions resembling those associated with retinoic acid syndrome.

7,9: Because they may induce symptoms of adverse reactions resembling those associated with hypervitaminosis A.

8: Because they are used in the concomitant therapy with interferon.

[2] Caution-required concomitant drugs: The following drugs should be administered with caution during the study drug administration period. The concomitant use of any of these drugs is prohibited from the day of the start of study drug administration until Week 8 after the start of administration.

|  | Drug classification | Generic name | Brand name |
| --- | --- | --- | --- |
| 1 | Ursodesoxycholic acid | Ursodesoxycholic acid | Ubiron, Ursamic, Urso, Urdeston, Urdex, Urdenacin, Gokumisin, Shikichol, Braue, Precoat, Reptor |
| 2 | Phenytoin | Phenytoin-sodium | Aleviatin, Hydantol, Phenytoin |
| 3 | Tetracyclines antibiotics | Tetracycline hydrochloride | Achromycin, Achromycin V |
|  |  | Demethylchlortetracycline hydrochloride | Ledermycin |
|  |  | Doxycycline hydrochloride | Paldomycin, Vibramycin, Piperamycin, Rasenamycin |
|  |  | Minocycline hydrochloride | Minocyclin hydrochloride, Coupelacin, Namimycin, Pardoclin, Minotowa, Minopen, Minomycin |
| 4 | Drugs interfering with glucuronate conjugation | Sodium valproate | Epirenat, Sanoten, Cebotval, Selenica R, Cereb, Depakene, Depakene R, Hyserenin, Baldeken R, Sodium Valproate, Valpram R, Valerin |
|  |  | Lorazepam | Azrogen, U-Pan, Rocosgen, Lorazepam, Wypax |

[Rationale]

1: Because the surface-active action of the drug itself may promote absorption of the components, thereby increasing the plasma concentrations

2: Because etretinate (Tigason) has been reported to decrease the protein-binding ability of phenitoin (however, interactions with this component are unknown)

3: Reports of other retinoid have shown that, when used concomitantly with tetracyclines antibiotics, it increased the incidence of headache

4: Because metabolism of this component may be inhibited and the plasma concentration may be increased

# 12. Statistic analysis

## 12.1 Tabulation of subject background

The subject background will be tabulated by group.

## 12.2 Analysis of gene expression profile

Compare the data on gene expression profile obtained before and after the study drug administration (days of evaluation at Week 0 and Week 8) by dose (300 mg/day and 600 mg/day) and by patient. The details about the method of analysis of the gene expression profile are presented in the “Manual for determination of the gene expression profile of NIK-333.”

It may become necessary to make additional analyses focused on the NIK-333 response gene on the basis of the results of the present analysis of gene expression profile. In such cases, revise the “Manual for determination of the gene expression profile of NIK-333.”

## 12.3 Safety analysis

Prepare tabulated lists of adverse events (symptoms and signs, etc.), and tabulate the data by type and severity. Concerning laboratory tests, prepare tabulated lists and time-course charts, and calculate summary statistics. Tabulate other observation parameters in the same way as laboratory tests.

## 12.4 Pharmacokinetics analysis

Tabulate and examine the data on liver, plasma and urinary drug concentrations.

## 12.5 Analysis of TGF-α

Tabulate and examine the data.

## 12.6 The number of subjects to be registered according to the plan

12 subjects

Divide subjects into the following two groups in Step I (Week 0 to Week 8).

NIK-333 300 mg group: 6 subjects

NIK-333 600 mg group: 6 subjects

All subjects will receive 600 mg/day in Step II (Week 8 to Week 96).

[Rationale]

This clinical pharmacology study is an exploratory study aimed at investigating the changes of gene expression profiles in the liver and peripheral blood and identifying the drug response gene for NIK-333 in patients who were completely cured of HCV-positive HCC before and after 8-week repeated administration of 300 mg/day or 600 mg/day of NIK-333. The initial target sample size has been set at 6 subjects per group, considering feasibility of the study at the study center.

## 12.7 Selection of the subjects to be analyzed and handling of missing data, excluded data and abnormal data

The sponsor will screen for ineligible cases and cases of deviation after registration. Ineligible cases are defined as cases with study protocol deviations before subject registration, including violation of the inclusion criteria and the exclusion criteria. Cases of deviation after registration are defined as cases with study protocol deviations after subject registration, including cases involving violation of concomitant drugs, violation regarding the determination of examination and observation parameters, and violation of the discontinuation criteria. The sponsor will decide the handling of ineligible cases, cases with study protocol deviations after subject registration, and other cases with any problems according to the following standards for handling in consultation with medical experts, etc.

(1) Gene expression profile

The objective of this study is to idendify the drug response gene from the gene expression profile of the subjects who have received NIK-333. Therefore, the primary results will be obtained from the analysis of Per Protocol Set (PPS). At the same time, the analysis of the Full Analysis Set (FAS) will also be performed.

[1] FAS

The population obtained by excluding the following subjects from among the registered subjects

1) Ineligible cases

-Cases with violation of “5.2 Inclusion criteria (1), (2), (3) and (5)-1)”

2) Cases of deviation after registration

-Subjects with no available data of gene expression analysis

- Subjects who have not taken the study drug at all during the period from the day of the start of study drug administration until the day of evaluation at Week 8 of study drug administration

[2] PPS

The population obtained by excluding the following subjects from the FAS

1) Ineligible cases

- Cases with violation of “5.2 Inclusion criteria (4), (5)-2) and -3), (6) and “5.3 Exclusion criteria”

2) Cases of deviation after registration

- Subjects for whom the compliance rate from the day of the start of study drug administration until the day of evaluation at Week 8 of study drug administration is less than 70%

- Other subjects with study protocol deviations (however, slight deviations, including partially missing data will be examined as needed.）

(2) Safety

- The “Safety Analysis Set” is obtained by eliminating the following subjects from the registered subjects.

[1] Cases of deviation after registration

- Subjects for whom safety data are not available

- Subjects who have not taken the study drug at all

(3) Pharmacokinetics

The “Pharmacokinetics Analysis Set” is obtained by eliminating the following subjects from among the registered subjects.

[1] Cases of deviation after registration

- Subjects for whom pharmacokinetics data are not available

- Subjects who have not taken the study drug at all

(4) Plasma TGF-α

The “Plasma TGF-α Analysis Set” is obtained by eliminating the following subjects from among the registered subjects.

[1] Cases of deviation after registration

- Subjects for whom the data of TGF-α concentrations in plasma are not available

- Subjects who have not taken the study drug at all

(5) Handling of missing data

If any values of vital signs, hematology tests, blood biochemistry tests, urinary tests, bone densitometry, endoscopy, ECG, echocardiography, pulse wave examination or fundus examination are missing because the examination or determination has not been conducted or for other reasons, the data will be handled as missing data.

## 12.8 Deviations from the initial statistic analysis plan

When new statistic analysis is conducted without carrying out the predefined statistic analysis plan, this study protocol will be modified.

# 13. Direct access to the source documents, etc.

## 13.1 Identification of source documents

Source documents are defined as all documents, data and records (hospital records, medical records, examination notebooks, nursing diary, data recorded by automatic measuring device, and records, etc. which are stored at the pharmacies or laboratories involved in the study), which become the source of information about the study.

## 13.2 Information directly recorded on case report forms to be regarded as source data

Among all kinds of information contained in the original records or their guaranteed photocopies regarding clinical findings, observations and other activities in the study, the following data which are directly recorded in case report forms only will be treated as the source data.

[1] Severity of adverse events, their outcomes, causal relationship with the study drug, and comments

[2] Status of protocol compliance

## 13.3 Direct access to source documents

The investigator and the study center, when requested, will allow inspections by the sponsor’s monitors, auditors, the Institutional Review Board and any regulatory authority as well as direct access to all study-related records, including the source documents.

# 14. Quality control and quality assurance of the study

## 14.1 Quality control of the study

(1) Sponsor

The sponsor will control the quality of the study according to the GCP Standard Operating Procedures of Kowa (hereinafter referred to as Kowa GCP-SOP).

(2) Study center

The study center and the investigator, etc. will comply with the study protocol. The study center and the investigator, etc. will carry out quality control in accordance with the regulations of the study center to guarantee that the study is conducted and the data are prepared, recorded and reported in compliance with the Ordinance GCP and the study protocol.

The sponsor will also cooperate with monitoring.

## 14.2 Quality assurance of the study

The sponsor will guarantee the quality of the study in accordance with the Kowa GCP-SOP prescribed by the sponsor.

# 15. Ethics

## 15.1 Approval of the Institutional Review Board

This clinical study will be conducted with an approval of the Institutional Review Board of the study center and an approval of the Appropriateness Assessment Committee of the sponsor.

## 15.2 Subjects’ informed consent

(1) Preparation of informed consent forms and written information

The investigator will prepare the informed consent forms and written information with cooperation of the sponsor, and obtain an approval of the Institutional Review Board of the study center. Moreover, the investigator will prepare the informed consent forms and written information regarding determination of the gene expression profile in addition to the informed consent forms and written information regarding participation in the study, and obtain approval of the Institutional Review Board of the study center.

(2) Information to be provided in writing

The following information will be included in the written information to be provided to the subjects for obtaining informed consent.

[1] That the trial involves research

[2] Study objectives

[3] Study methods

[4] Determination of gene expression profile

[5] The expected duration of the subject’s participation in the study

[6] The number of subjects who are to participate in the study

[7] Reasonably expected clinical benefits and foreseeable risks or inconveniences

[8] The alternative procedures of treatment that may be available to the subject and their important potential benefits and risks

[9] The compensation and treatment available to the subject in the event of study-related health injury

[10] That the subject’s participation in the study is voluntary and that the subject or the subject and his/her representative may refuse to participate or withdraw from the study, at any time, without penalty or loss of benefits to which the subject is otherwise entitled

[11] That the subject or the subject and his/her representative will be informed in a timely manner if information becomes available that may be relevant to the willingness of the subject or the subject and his/her representative to continue participation in the study

[12] The foreseeable circumstances and/or reasons under which the subject’s participation in the study may be terminated

[13] That the monitors, the auditors, the Institutional Review Board and the regulatory authority will be granted direct access to the subject’s original medical records, without violating the confidentiality of the subject, and that, by sealing or signing a written informed consent form, the subject or the subject and his/her representative is or are authorizing such access.

[14] That, even if the results of the study are published, the subject’s identity will remain confidential.

[15] The anticipated expenses, if any, to the subject for participating in the study

[16] The anticipated prorated payment, if any, to the subject for participating in the study

[17] The name, title and contact information of the investigator or the subinvestigator

[18] The person to contact at the study center when the subject or the subject and his/her representative need(s) to obtain further information regarding the study and the rights of subjects, and the person to contact in the event of study-related health injury

[19] The subject’s responsibilities

(3) Giving information in writing and obtaining informed consent

The investigator or the subinvestigator will give an informed consent form and written information to a prospective subject who meets the inclusion criteria and does not fall under any of the exclusion criteria or to the subject and his/her representative, provide thorough explanation about the study and other matters related to the study, and obtain a voluntary consent to participate in the study from the subject or the subject and the subject’s representative in writing. The following points should be kept in mind when obtaining the informed consent.

[1] The investigator and the subinvestigator must not persuade the subject or the subject and his/her representative to participate in the study or to continue participation in the study by coercion or by unjustifiable influences.

[2] Any phrases making or suggesting to make the subject or the subject and his/her representative give up their rights, or any phrases exempting or suggesting to exempt the investigator, the subinvestigator, the study center or the sponsor from any legal responsibilities must not be included in the informed consent forms, written information and the information orally provided to the subject.

[3] The investigator or the subinvestigator must give the subject or the subject and his/her representative chances to ask questions and ample time to think over whether or not to participate in the study before obtaining the consent. The investigator and the subinvestigator must answer all questions to the satisfaction of the subject or the subject and his/her representative.

(4) Signing, etc. and issuance of the informed consent form

The investigator or the subinvestigator who has given briefing about the study will provide his/her name, affix his/her seal or signature, and enter the date of briefing on the informed consent form. If the subject or the subject and his/her representative agree(s) to participate in the study, he/she or they will be asked to sign the informed consent form and to enter the date of informed consent. After obtaining the informed consent, the investigator or the subinvestigator will give a copy of the informed consent form along with the written information to the subject or the subject and his/her representative, and the study center will keep the original copy of the informed consent form.

(5) Provision of information and confirmation of willingness to continue participation

When any information becomes available that may be relevant to the willingness of the subject or the subject and his/her representative to continue participation in the study or that may be relevant to the subject’s consent, the investigator or the subinvestigator will immediately provide the information to the subject and confirm the willingness of the subject or the subject and the subject’s representative to continue the study.

(6) Revision of informed consent forms and written information

When any revisions of the informed consent form or written information have become necessary due to the information mentioned above in (5), the investigator will immediately revise the informed consent form and the written information based on that information with cooperation of the sponsor, and obtain an approval of the Institutional Review Board of the study center. Moreover, the investigator or the subinvestigator will conduct briefing to the subject or the subject and his/her representative again using the revised informed consent form and written information and obtain the subject’s consent to continue participation in the study in writing. After obtaining the subject’s consent, the investigator or the subinvestigator will hand a copy of the informed consent form along with written information to the subject or the subject and his/her representative, and the study center will keep the original copy of the informed consent form.

## 15.3 Protection of subjects’ privacy

The investigator, the subinvestigator and the sponsor, etc. will pay due attention to protection of the subjects’ privacy when handling case report forms, gene expression profile data, imaging data, source data related to the conduct of the study, and informed consent forms.

# 16. Handling of data and storage of records

## 16.1 Essential documents to be stored

(1) Study center

[1] Source documents

[2] Documents or their copies prepared by those working at the study center according to the clinical study contract and the provisions of the Ordinance GCP

[3] Study protocol, case report forms (copy), documents obtained from the Institutional Review Board according to the provisions of Article 32, Clause 1 and Clause 2 of the Ordinance GCP, and documents obtained according to the other provisions of Ordinance GCP

[4] Records of management of the study drugs

[5] Records of other study-related works

(2) Institutional Review Board

[1] Manuals and list of members prescribed in Article 28, Clause 2 of Ordinance GCP

[2] Documents listed in the paragraphs of Article 32, Clause 1 of Ordinance GCP

[3] Notifications to the Institutional Review Board and records of the meetings of Institutional Review Board prescribed in Article 40, Clause 1 to Clause 4 of Ordinance GCP

(3) Sponsor

[1] Study protocol, clinical study contract, clinical study report, and other documents prepared by the sponsor according to the provisions of Ordinance GCP or reproduced copies of these documents

[2] Case report forms, documents of notification prescribed in Article 32, Clause 4 of Ordinance GCP, and other records obtained from the head of the study center or the investigator according to the other provisions of Ordinance GCP

[3] Records of monitoring, auditing and other works related to sponsoring and management of the study

[4] Data obtained through the conduct of the study

[5] Records prescribed in Article 16, Clause 5 of Ordinance GCP

## 16.2 Responsibility for storing and storing period

(1) Study center

For storing records, the head of the study center will appoint a person responsible for storage of the records.

The person responsible for storage will store the essential documents to be stored at the study center for the period prescribed below in (1) or (2), which comes later. If the sponsor requires the documents to be stored for a period longer than this, the sponsor will consult with the head of the study center to decide the storing period and the storing method.

[1] The day of approval for the manufacturing and marketing of a drug related to the study drug concerned (or 3 years after the day on which discontinuation of development is decided, if development of the drug is discontinued)

[2] The day on which 3 years have passed since the discontinuation or completion of the clinical study

(2) Institutional Review Board

The person who has established the Institutional Review Board will store the essential documents to be stored at the Institutional Review Board for the period prescribed below in (1) or (2), which comes later. If the sponsor requires the documents to be stored for a longer period, the sponsor will deliberate on the storing period and the storing method.

[1] The day of approval for the manufacturing and marketing of a drug related to the study drug concerned (or the day on which discontinuation of development is decided, if development of the drug is discontinued)

[2] The day on which 3 years have passed since the discontinuation or completion of the clinical study

(3) Sponsor

The sponsor must store the essential documents to be stored at the sponsor for the period prescribed below in (1) or (2), which comes later.

[1] The day on which 5 years have passed from the day of approval for the manufacturing and marketing of a drug related to the study drug concerned (or 3 years after the day on which discontinuation of development is decided, if development of the drug is discontinued), or the day of completion of re-examination, which ever comes later

[2] The day on which 3 years have passed since the discontinuation or completion of the clinical study

(4) Notification of completion of the storing period

When it is no longer necessary to store the documents that are required to be stored by the head of the study center or the person who has established the Institutional Review Board, the sponsor will notify the head of the study center or the person who has established the Institutional Review Board, through the head of the study center, to that effect.

# 17. Payment and compensation

## 17.1 Payment

Payment related to the clinical study will be set forth in writing between the sponsor and the study center before the start of the study.

## 17.2 Compensation

When any health injury has occurred to the subjects due to the conduct of the study, the sponsor will be responsible for the compensation unless it is proven that the injury has been induced by deliberation or gross negligence of the study center or the subject him- or herself.

The contents of compensation will be the amount of self-payment of the medical expense which is not covered by the benefit of health insurance, medical allowance based on the Adverse Drug Reaction Suffering Relief Law, and compensation payment. The sponsor must take necessary precautionary measures, including insurance, to assume the liability for compensation. Furthermore, when any liability for reparation has occurred in connection with this clinical study, the sponsor will assume the liability unless the study center is obviously responsible for the event.

When it is ambiguous as to who is responsible for the event concerned, the study center and the sponsor will consult with each other in good faith to solve the problem. The details about compensation are described in the “Outline of compensation.”

# 18. Arrangement regarding publication

Due attention should be paid to protection of the subjects’ privacy in the treatment of case report forms, source data regarding the conduct of the study, and informed consent forms. During preparation of case report forms, subject ID codes will be used to identify individual subjects. Attention should also be paid to protection of the subjects’ privacy when publishing the results of this clinical study (submission to medical journals, submission of applications to the Ministry of Health, Labour and Welfare, etc.). The sponsor will not use the case report forms prepared for any purposes other than this clinical study. When the results obtained from this clinical study are publicized in academic conferences or technical journals, the investigator or the subinvestigator will obtain prior consent from the sponsor in writing.

# 19. Study period

September 2005 to December 2009

# 20. Protocol agreement/compliance, deviations/changes and revisions

## 20.1 Protocol agreement and compliance

The sponsor’s study manager and the investigator shall agree with the contents of the study protocol and case report forms and further agree to comply with the study protocol by providing his/her name, affixing his/her seal or signature and the date on the agreement document prescribed separately. The same procedure should be followed when the study protocol and the case report forms have been revised.

## 20.2 Protocol deviations, changes and revisions

[1] The investigator or the subinvestigator must not deviate from or change the study protocol without prior agreement with the sponsor in writing and prior approval of the Institutional Review Board in writing based on prior examination. However, in the situations where the deviation or change cannot be avoided for medical reasons, such as the need to avert an urgent risk to subjects, the investigator or the subinvestigator can deviate from or change the study protocol without prior agreement with the sponsor in writing or prior approval of the Institutional Review Board. In such cases, the investigator will notify the head of the study center as soon as possible of the content of the deviation or change and the reason, or the draft of any revisions, if protocol revisions are necessary, and obtain the approval of the Institutional Review Board. Moreover, the investigator or the subinvestigator will obtain an agreement in writing from the sponsor via the head of the study center.

[2] When any protocol deviations have occurred, the investigator will record the deviation, submit a report about the deviation and the reasons to the sponsor, and keep a reproduced copy of the report.

[3] The investigator will immediately submit to the sponsor, head of the study center and the Institutional Review Board a report about any changes which may have a major influence on the conduct of the study and may greatly increase the risk to subjects.

[4] The “7.8 Acceptable range of deviations from the dates of examination, observation and evaluation” will be followed regarding deviations to the days of each evaluation.

# 21. Procedures of completion and correction of case report forms

## 21.1 Case report forms

Use “Clinical Pharmacology Study of NIK-333, Case Report Forms” [Step I] and [Step II] as the case report forms.

## 21.2 Precautions for completion of case report forms

Follow the procedures provided in “Clinical Pharmacology Study of NIK-333, Case Report Forms, Guidance on Completion and Correction” when completing the case report forms.

## 21.3 Procedures for correction of case report forms

Use “Clinical Pharmacology Study of NIK-333, Case Report Forms, Correction Record Form” when making changes, additions or deletions in the information entered in the case report forms.

# 22. References

1) Vital Statistics of Japan, 2002, Statistics and Information Department of the Ministry of Health, Labour and Welfare.

2) White Paper on Hepatic Cancer 1999, The Japan Society of Hepatology, 1999.

3) The 16^th^ report of National Primary Hepatic Cancer Follow-up Examination (2000 to 2001), Liver Cancer Study Group of Japan, 2004.

4) Shiratori Y, *et al.*: Interferon therapy after tumor ablation improves prognosis in patients with hepatocellular carcinoma associated with hepatitis C virus. *Ann. Intern. Med.* 138: 299-306, 2003.

5) Imazeki F, *et al.*: Chronic hepatitis C. Histological improvement and inhibition of hepatic carcinogenesis in the cases treated with IFN. *Kanzo* (Acta Hepatologica Japonica) Vol. 39 10: 783-784, 1998.

6) Ikeda K, *et al.*: Interferon beta prevents recurrence of hepatocellular carcinoma after complete resection or ablation of the primary tumor:A prospective randomized study of hepatitis C virus-related liver cancer. *Hepatology* 32: 228-232, 2000.

7) Suou T, *et al.*: Interferon alpha inhibits intrahepatic recurrence in hepatocellular carcinoma with chronic hepatitis C:a pilot study. *Hepatol. Res.* 20: 301-311, 2001.

8) Kubo S, *et al.*: Effects of long-term postoperative interferon-α therapy on intrahepatic recurrence after resection of hepatitis C virus-related hepatocellular carcinoma. *Ann. Intern. Med.* 134: 963-967, 2001.

9) Nishiguchi S, *et al*.:Prevention of recurrent hepatocellular carcinoma by interferon. New strategy for treatment of chronic hepatitis C. Future perspective of interferon therapy. Edited by Hayashi N, *et al.* Sentanigaku-sha, pp103-108, 2004.

10) Sporn MB and Newton DL: Chemoprevention of cancer with retinoids. *Fed. Proc.* 38: 2528-2534, 1979.

11) Muto Y, *et al.*: In vitro binding affinity of novel synthetic polyprenoids (polyprenoic acids) to cellular retinoid-binding proteins. *Gann* 72: 974~977, 1981.

12) E5166 Clinical Study Team: Results of a clinical study of E5166 in skin diseases -- Examination by Open Study --, Nishi Nichi Hifu (Nishinion Journal of Dermatology) Vol. 48, 513-519, 1986.

13) Muto Y, *et al.*: Prevention of second primary tumors by an acyclic retinoid, polyprenoic acid, in patients with hepatocellular carcinoma. *N. Engl. J. Med.* 334: 1561-1567, 1996.

14) Muto Y, *et al.*: Prevention of second primary tumors by an acyclic retinoid in patients with hepatocellular carcinoma. *N. Engl. J. Med.* 340: 1046-1047, 1999.

15) Kagawa M, *et al.*: An acyclic retinoid, NIK-333, inhibits N-diethylnitrosamine-induced rat hepatocarcinogenesis through suppression of TGF-α expression and cell proliferation. *Carcinogenesis* 25: 979-985, 2004.

16) Kusano N, *et al*.: Mechanism of carcinogenesis. *Igaku to Yakugaku* (Journal of Medicine and Pharmaceutical Science) 43: 1021-1026, 2000.

17) Suzui M, *et al.*: Growth inhibition of human hepatoma cells by acyclic retinoid is associated with induction of p21(CIP1) and inhibition of expression of cyclin D1. *Cancer Res.* 62: 3997-4006, 2002

18) Nakamura N, Apoptosis in human hepatoma cell line induced by 4,5-didehydro geranylgeranoic acid (acyclic retinoid) via down-regulation of transforming growth factor-alpha. Biochem Biophys Res Commun. 219: 100-104, 1996

19) Davies TS and Monro A: Marketed human pharmaceuticals reported to be tumorigenic in rodents. *J. Am. Toxicol.* 14: 90-107, 1995.

20) Kelloff GJ, *et al.*: Clinical development plan:N-(4-Hydroxyphenyl) retinamide (4-HPR). *J. Cell Biochem. Suppl.* 20: 176-196, 1994.

21) Bogovski P: Tumors of the skin. In“Pathology of tumors in laboratory animals. Volume II-Tumors of the mouse”. pp1-41, *International Agency for Research on Cancer*, Lyon, 1979.

22) Elwell MR: Heart, Blood Vessels, and Lymphatics. In “Pathology of the mouse”. pp361-380, *Cache River Press*, USA, 1999.

23) Ikeda K, *et al.*: A multivariate analysis of risk factors for hepatocellular carcinogenesis: A prospective observation of 795 patients with viral and alcoholic cirrhosis. *Hepatology* 18: 47-53, 1993.

24) Pugh RNH, *et al.*: Transection of the oesophagus for bleeding oesophageal varices. *Br. J. Surg.* 60: 646-649, 1973.

25) General rules for the clinical and pathological study of primary liver cancer [Version 4], Liver Cancer Study Group of Japan, 2000.

Table 1 Examination, observation and evaluation parameters at registration

| Examination parameters | Examination, observation and evaluation parameters |
| --- | --- |
| Abdominal imaging/  diagnosis | Presence or absence of HCC on dynamic CT images |
| Medical examination | Symptoms*, signs |
| Subject background | Gender, age, diameter and number of primary tumors before topical medical therapy or surgical resection, underlying disease, previous history |
| Blood pressure | Blood pressure (systolic/diastolic: dorsal position or sitting position) |
| Child-Pugh classification | Liver encephalopathy, ascites, serum bilirubin, serum albumin, prothrombin time |
| HCV-RNA | Quantitative |
| HBs antigen | Qualitative |
| HCG tests | Pregnant or not (premenopausal women only) |
| Hematology tests | Platelet count, hemoglobin level |

*: Menopausal status and whether or not being pregnant will be confirmed for female subjects.

Table 2 Examination, observation and evaluation parameters during the study drug administration period (including the day of the start of administration)

| Examination parameters | Examination, observation and evaluation parameters |
| --- | --- |
| Gene expression analysis | Gene expression profile in the liver and peripheral blood |
| Exploratory biomarker | TGF-α concentration in plasma (biomarker associated with gene expression) |
| Pharmacokinetics | Drug concentrations in the liver, plasma and urine  (unchanged NIK-333 and NIK-333 lipids form) |
| Abdominal imaging/  diagnosis | Presence or absence of HCC on dynamic CT, US, and MRI images |
| Medical examination | Symptoms*, signs |
| Vital signs, etc. | Body temperature (axillary), blood pressure (systolic/diastolic: dorsal position or sitting position), pulse rate (dorsal position or sitting position), body height^#^, body weight |
| HCV-RNA | Quantitative (including genotype analysis^#^) |
| HCG tests | Pregnant or not (premenopausal women only) |
| Hematology tests | White blood cell count, red blood cell count, hemoglobin level, hematocrit, platelet count |
| Blood biochemistry tests | Total protein, albumin, bilirubin, total cholesterol, LDL cholesterol, HDL cholesterol, triglyceride, blood glucose, urea nitrogen, Cl, Na, K, Ca, ALP, AST, ALT, LDH, γ-GTP, choline esterase, serum creatinine, A/G ratio, free fatty acid, β2-microglobulin |
| Blood pressure parameters | Blood biochemistry tests: Renin activity, aldosterone, cortisol, uric acid, FT3, FT4, TSH, angiotensin II, hANP, BNP, catecholamine, growth hormone, dehydroepiandrosterone sufate (DHEAS)  Urinary tests (accumulated urine): creatinine clearance |
| Tumor markers | AFP，AFP-L_3_，PIVKA-II |
| Fibrosis marker | Hyaluronic acid |
| Urinary tests (fresh urine) | Qualitative (glucose, protein, urobilinogen, ketone body, occult blood)  urinary albumin (converted to creatinine), urinary sediments (red blood  cells, white blood cells, epithelial cells, columnar cells, others)  β2-microglobulin |
| Bone densitometry (DXA) | Bone density of lumbar vertebrae (L_2_ to L_4_) |
| Endoscopy | Esophagus, stomach |
| ECG | 12-lead |
| Echocardiography | M mode echocardiogram, two-dimensional echocardiogram, Doppler echocardiogram, etc. |
| Pulse wave examination | Pulse wave velocity (PWV)/ankle brachial index (ABI) |
| Fundus examination | Fundus photographs |

*: Menopausal status and whether or not being pregnant will be confirmed for female subjects.

#:Conducted only on the day of the start of administration

Table 3 Examination, observation and evaluation parameters at the time of follow-up examination

| Examination parameters | Examination, observation and evaluation parameters |
| --- | --- |
| Pharmacokinetics | Drug concentration in plasma (unchanged NIK-333 and NIK-333 lipids form) |
| Abdominal imaging/  diagnosis | Presence or absence of HCC on dynamic CT, US, and MRI images |
| Medical examination | Symptoms*, signs |
| Vital signs, etc. | Body temperature (axillary), blood pressure (systolic/diastolic: dorsal position or sitting position), pulse rate (dorsal position or sitting position), body height^#^, body weight |
| Hematology tests | White blood cell count, red blood cell count, hemoglobin level, hematocrit, platelet count |
| Blood biochemistry tests | Total protein, albumin, bilirubin, total cholesterol, LDL cholesterol, HDL cholesterol, triglyceride, blood glucose, urea nitrogen, Cl, Na, K, Ca, ALP, AST, ALT, LDH, γ-GTP, choline esterase, serum creatinine, A/G ratio, free fatty acid, β2-microglobulin |
| Tumor markers | AFP，AFP-L_3_，PIVKA-II |
| Urinary tests (fresh urine) | Qualitative (glucose, protein, urobilinogen, ketone body, occult blood)  urinary albumin (converted to creatinine), urinary sediments (red blood  cells, white blood cells, epithelial cells, columnar cells, others)  β2-microglobulin |
